# A brain cell atlas integrating single-cell transcriptomes across human brain regions

**DOI:** 10.1038/s41591-024-03150-z

**Published:** 2024-08-02

**Authors:** Xinyue Chen, Yin Huang, Liangfeng Huang, Ziliang Huang, Zhao-Zhe Hao, Lahong Xu, Nana Xu, Zhi Li, Yonggao Mou, Mingli Ye, Renke You, Xuegong Zhang, Sheng Liu, Zhichao Miao

**Affiliations:** 1 Guangzhou National Laboratory, Guangzhou International Bio Island, Guangzhou, China; 2 State Key Laboratory of Ophthalmology, Zhongshan Ophthalmic Center, Sun Yat-sen University, Guangdong Provincial Key Laboratory of Ophthalmology and Visual Science, Guangzhou, China; 3 Department of Neurosurgery/Neuro-oncology, State Key Laboratory of Oncology in South China, Guangdong Provincial Clinical Research Center for Cancer, Sun Yat-sen University Cancer Center, Guangzhou, China; 4 Tsinghua Fuzhou Institute for Data Technology, Fuzhou, China; 5 MOE Key Lab of Bioinformatics, Bioinformatics Division of BNRIST and Department of Automation, Tsinghua University, Beijing, China; 6 School of Medicine, Tsinghua University, Beijing, China; 7 School of Life Sciences, Center for Synthetic and Systems Biology, Tsinghua University, Beijing, China; 8 Guangdong Province Key Laboratory of Brain Function and Disease, Guangzhou, China; 9 Shanghai Key Laboratory of Anesthesiology and Brain Functional Modulation, Clinical Research Center for Anesthesiology and Perioperative Medicine, Translational Research Institute of Brain and Brain-Like Intelligence, Shanghai Fourth People’s Hospital, School of Medicine, Tongji University, Shanghai, China; 10 GMU-GIBH Joint School of Life Sciences, The Guangdong-Hong Kong-Macau Joint Laboratory for Cell Fate Regulation and Diseases, Guangzhou National Laboratory, Guangzhou Medical University, Guangzhou International Bio Island, Guangzhou, China; 11 Bioland Laboratory (Guangzhou Regenerative Medicine and Health Guangdong Laboratory), Guangzhou International Bio Island, Guangzhou, China

**Keywords:** RNA sequencing, Genetic databases

## Abstract

While single-cell technologies have greatly advanced our comprehension of human brain cell types and functions, studies including large numbers of donors and multiple brain regions are needed to extend our understanding of brain cell heterogeneity. Integrating atlas-level single-cell data presents a chance to reveal rare cell types and cellular heterogeneity across brain regions. Here we present the Brain Cell Atlas, a comprehensive reference atlas of brain cells, by assembling single-cell data from 70 human and 103 mouse studies of the brain throughout major developmental stages across brain regions, covering over 26.3 million cells or nuclei from both healthy and diseased tissues. Using machine-learning based algorithms, the Brain Cell Atlas provides a consensus cell type annotation, and it showcases the identification of putative neural progenitor cells and a cell subpopulation of *PCDH9*^high^ microglia in the human brain. We demonstrate the gene regulatory difference of *PCDH9*^high^ microglia between hippocampus and prefrontal cortex and elucidate the cell–cell communication network. The Brain Cell Atlas presents an atlas-level integrative resource for comparing brain cells in different environments and conditions within the Human Cell Atlas.

## Abstract (web summary)

A single-cell transcriptomic study from the Human Cell Atlas integrating over 11 million brain cells from 70 studies uncovers differences across human brain regions and identifies rare progenitor and microglia subtypes.

## Main

Over 170 years ago, the accident of American railroad worker Phineas Gage first revealed that specific brain regions were important for certain functions. Since then, various research work has been performed to understand the brain regions, brain cells and their functions, while the rapid progress of single-cell technologies^1^ in the past decade accelerated the discovery of neuronal cell types^2,3^, providing insights into regional microenvironment and lineage specialization. Especially consortia like Human Cell Atlas^4^, HuBMAP^5^, BICCN^6^ and Allen Brain Atlas^7^ have accumulated extensive datasets, providing large-scale reference atlases of human brain cells^8^. Tasic et al.^9^ found that most glutamatergic neurons are area specific, while nonneuronal and most GABAergic neuron types are shared across mouse cortical areas. Siletti et al.^10^ sampled more than 3 million (M) cells from approximately 100 locations across the adult human forebrain, midbrain and hindbrain. Braun et al.^11^ mapped the differentiation trajectories of over 1.6M cells into 616 clusters in the first-trimester human forebrain and midbrain.

With some exceptions, most existing studies were restricted to a single region, a small portion of the cells or a certain disease and were archived by separated datasets, leaving some cell types or cell states associated with diseases and developmental processes unexplored. Notably, aggregating data from various datasets may enrich information of these cell types, thus rendering vital discoveries, including neurogenesis at different ages^12–14^, rare cell discovery^8^, regional heterogeneity^9,15^ and the contributions of cell types to neurodegenerative disease^16,17^. As an example, the identification of rare neural progenitor cell (NPC) populations in adults remains difficult and controversial^12,18–20^. Additionally, the diversity and plasticity of microglia have intensified the debate on how to accurately define their subtypes^21,22^. Considering most of the current studies focus on a single brain region, understanding the microglia regional heterogeneity and phenotypic differences across brain regions remains difficult. Fortunately, the integration of large-scale published datasets may provide a more complete landscape of the brain cells, thus leading to the exploration of rare cell populations or comparison of cells across brain regions.

In this resource, we present the Brain Cell Atlas, a unified single-cell atlas of the human brain assembled from 70 studies, with 11.3M cells or nuclei that covered nearly all major regions of the brain in health and diseases as well as 103 studies of mouse data of 15M cells. We demonstrate the utilities of the Brain Cell Atlas in the discovery of putative NPCs in adults and in understanding the microenvironment-driven difference of microglia. The atlas will serve as a valuable resource for studying brain cells and functions, enhancing our understanding of neuronal processes and neurodegenerative diseases.

## Results

### Overview of the Brain Cell Atlas

The resource, which is also provided as an interactive web portal, includes 11.3M human cells from 14 main regions and 30 subregions of the brain (Supplementary Fig. 1), while the mouse data include 15M cells. Single-cell RNA sequencing and single-nucleus RNA sequencing data of the brain were searched through literature and the single-cell database^23^, covering over 1,800 published datasets deposited in Gene Expression Omnibus (GEO)^24^, the UCSC browser^25^, ArrayExpress^26^, Allen Brain Map (https://portal.brain-map.org/) and Synapse (https://www.synapse.org/) (Supplementary Fig. 2). The resource covers 70 human studies of 6,577 samples (Supplementary Fig. 3a–c), along with 103 mouse studies of 25,710 samples. The metadata were manually curated and raw counts were collected (Methods, Supplementary Fig. 2 and Supplementary Table 1) in a consistent manner. Two well-established datasets were used as refs. ^10,11^ to infer cell type labels in other datasets using reference-based machine learning algorithms. The adult ref. ^10^ contains 3.3 M nuclei from tissues of four post-mortem healthy adults aged from 29 to 60 years across the whole brain, while the fetal ref. ^11^ contains 1.6 M cells from the first-trimester developing brain tissues.

**Fig. 1:**
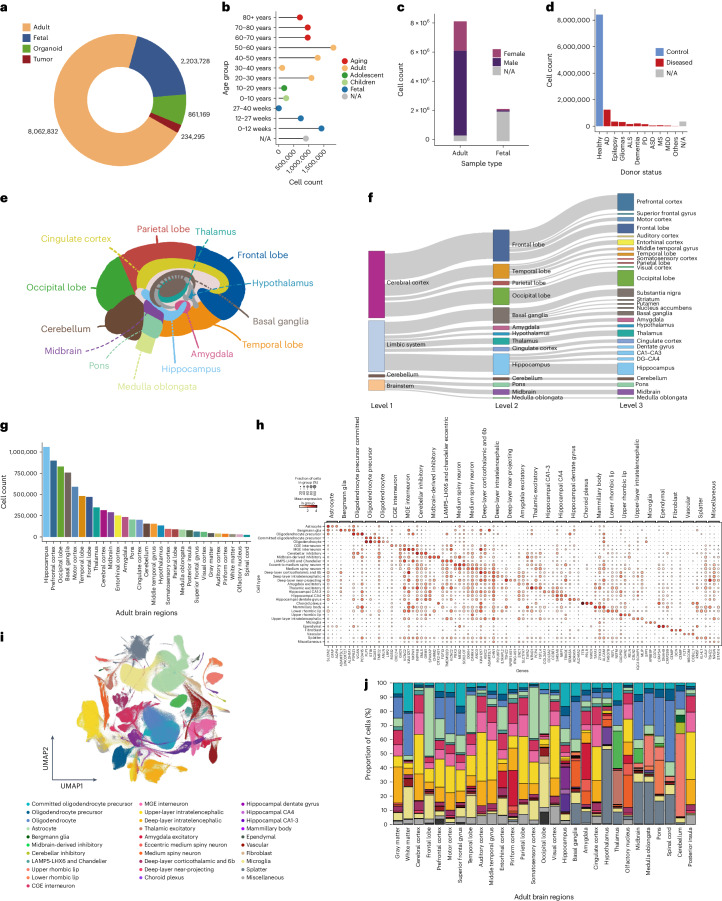
Statistics of the Brain Cell Atlas. **a**, Circular plot showing the proportions of the four primary sample types in the Brain Cell Atlas: adult (*n* = 8,062,832), fetal (*n* = 2,203,728), organoid (*n* = 861,169) and tumor (*n* = 234,295). A fraction of nonadult postnatal samples ranging from 0 to 20 years old were included in the adult brain data (see **b**). **b**, Bar plots showing the distribution of cell numbers across various age groups in human samples, spanning 6 to 39 GW in fetal samples and from 0 to over 80 years of age in adults. N/A indicates that age information is not available in the original publication. **c**, Stacked bar plot showing the proportions of donor sex in adult and fetal samples. N/A denotes samples with unavailable sex information. **d**, Histogram showing the cell counts categorized by donor status in both healthy and diseased conditions. ‘Gliomas’ include glioblastomas, oligodendroglioma, astrocytoma, mixed glioma and so on. ‘Others’ include carcinoma and mild cognitive impairments. N/A represents that medical condition information is not accessible. **e**, Anatomical depiction of the main regions where samples were collected in the Brain Cell Atlas. **f**, Hierarchical representation of anatomical structures in the adult brain, with line thickness reflecting cell proportions in each region. **g**, Histogram showing the cell counts per region in the adult brain data. **h**, Dot plot showing the cell markers derived from adult brain data, along with the top two relevant markers for each region. **i**, UMAP visualization of the adult brain data achieved by label transfer of reference data, showcasing cell type proportions across different brain regions. **j**, Stacked bar plot showing the cell type distribution in different brain regions. The color codes are the same as those listed in **i**. Source data

The human brain datasets were sorted into four types based on the sample source: adult (8,062,832 nuclei and cells), fetal (2,203,728 cells), organoids (861,169 cells) and brain tumor (234,295 cells) (Fig. 1a), while 94.8% cells were sequenced with 10x Chromium (Supplementary Fig. 3a) covering a time span from 6 gestational weeks (GW) to over 80 years old (Fig. 1b). In total, 46.4% of the fetal cells were from the first trimesters (0–12 GW), while postnatal cells were mainly (68.3%) from 40–80-year-old donors. As for sex in adult data, cells from female, male and unknown sex take up 24.6%, 70.7% and <5%, respectively. The sex for most (91.7%) cells from the fetal samples were undetermined, leaving a female-to-male ratio of 1.3:1 in the rest (Fig. 1c). For disease status, 74.1% were healthy samples and 3.1% were unspecified, while disease samples were dominated by Alzheimer’s disease (AD) followed by epilepsy, gliomas (glioblastomas, oligodendroglioma, astrocytoma, mixed glioma and so on), amyotrophic lateral sclerosis (ALS), major depressive disorder (MDD), autism spectrum disorder (ASD), dementia, Parkinson’s disease (PD) and multiple sclerosis (MS) (Fig. 1d). As for brain regions, the resource covers major cerebral cortex regions (frontal lobe, parietal lobe, occipital lobe and temporal lobe), cerebellum, brain stem (midbrain, pons and medulla oblongata) and the limbic system (hippocampus, thalamus, hypothalamus and amygdala) (Fig. 1e,f). Most cells or nuclei were collected from the hippocampus (13.1%), followed by prefrontal cortex (11.1%), occipital lobe (10.3%) and basal ganglia (9.4%) (Fig. 1g).

As an integrative resource, a consensus cell type annotation of all the adult data was derived from seven well-established reference-based machine learning methods (Methods) as well as an in-house built hierarchical annotation workflow (scAnnot). This general cell type annotation based on the eight machine learning methods may help with the selection of target data for specific analysis. The consensus cell type annotation resulted in 32 primary clusters on the Uniform Manifold Approximation and Projection (UMAP) visualization (Fig. 1i), while cell type-specific differentially expressed genes (DEGs) can be derived (Methods and Fig. 1h). The cell type composition across brain regions indicates the regional specificity and heterogeneity (Fig. 1j). For instance, upper-layer and deep-layer intratelencephahlic neurons are more abundant in cortex regions than hippocampus.

### Atlas-level hierarchical cell type annotation with scAnnot

**Fig. 2:**
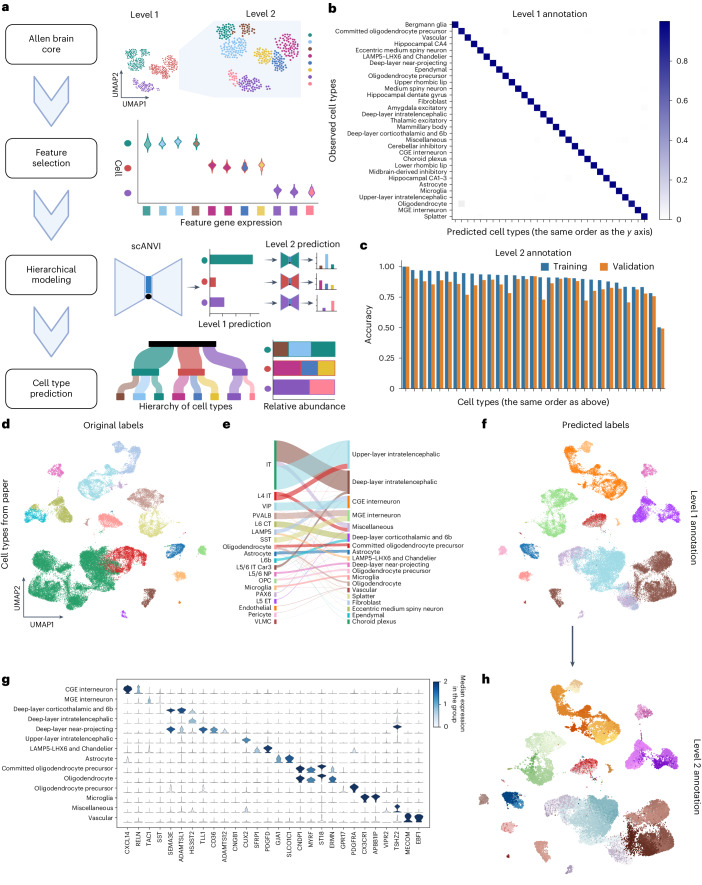
Atlas-level hierarchical cell type annotation. **a**, Schematic diagram illustrating the scAnnot tool (created with BioRender.com). This hierarchical classification model, based on scANVI, categorizes cells into cell types. The first level of classification groups cells into broad cell types, while the second level further classifies cells into more specific types within each broad category. The algorithm can be performed iteratively in classifying the cell types. **b**, Heatmap demonstrating the prediction accuracy for the first-level cell types. The rows represent the cell types reported in the publications, while the columns represent the scAnnot-predicted cell types. The color intensity represents the accuracy of the predictions, with darker colors indicating higher accuracy. **c**, Bar plot showing training and validation accuracies for the second layer of cell types. The *x* axis represents the broad cell types, while the *y* axis indicates the prediction accuracy. The blue and orange bars represent the training and validation accuracies, respectively. **d**, UMAP visualizing the reported cell types in the published data, with colors indicating the reported cell types. **e**, River plot illustrating the transition between reported and predicted first-level cell types. The left side represents the reported cell types, while the right side displays the scAnnot-predicted first-level cell types. **f**, UMAP visualization of the first-level scAnnot-predicted cell types. **g**, Stacked violin plot depicting the expression levels of select feature genes in the published data used by the scAnnot tool. The *y* axis represents the expression level, while the *x* axis denotes the gene names. The rows represent different cell types. **h**, UMAP visualization of the second-level scAnnot-predicted cell types. Source data

To achieve a multigranularity cell type annotation, we present scAnnot, a hierarchical cell annotation workflow based on the Variational Autoencoder model from single‐cell ANnotation using Variational Inference (scANVI^27^) (Methods and Fig. 2a). Although 45 out of the 70 datasets have their cell type annotations available (Supplementary Fig. 3b), the lack of consensus annotations hinders data integration of the resource. The cell types in the brain appear in a hierarchical manner of different granularities, which cannot be considered in the well-established reference-based machine learning methods. scAnnot trains machine learning models at different resolutions (granularities) and applies these models in a hierarchical structure. Using the adult reference of 31 primary cell types at the first-level of annotation, scAnnot selects 200 feature genes for each cell type-trained machine learning model with different hyperparameters (Supplementary Fig. 4). Then, it predicts the harmonized latent space of the cells, based on which the cell type labels can be inferred.

The annotation accuracy can be assessed by the confusion matrix^28^ between the reported cell type labels and the scAnnot-predicted labels (Fig. 2b,c). Most cell types can be predicted with high accuracy (above 93%), and the average accuracy is 98%. The second-level (with a finer granularity) cell type annotation achieves accuracies of 90% and 83% on the training and validation sets, respectively. Both visual inspection on UMAP and quantitative evaluation of Silhouette score^29^ indicate that the integrated data are not much affected by the batch effect (Supplementary Fig. 5). The classification accuracy of the subpopulations in each cell type ranged from 50% to 100%, with Splatter cluster being the least discriminative, as expected (Fig. 2c).

The primary cell types labels inferred by scAnnot are consistent with published annotation (Fig. 2d), while scAnnot further divides the intratelencephalic (IT) population into upper-layer intratelencephalic, deep-layer intratelencephalic and some miscellaneous (Fig. 2e,f). These cell clusters annotated by scAnnot can be confirmed by the feature gene expression (Fig. 2g). The hierarchical classification approach can further identify subpopulations at the second-level annotation with finer granularity (Fig. 2h).

### Potential NPCs in adult hippocampus

**Fig. 3:**
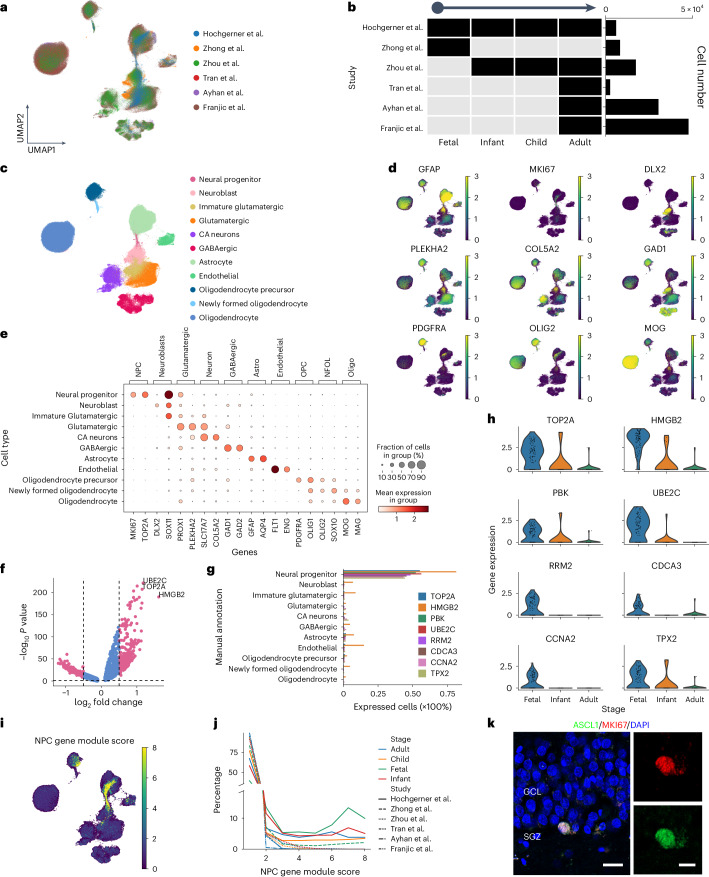
Integrating Brain Cell Atlas data to explore the existence of AHN. **a**, UMAP visualization of the integrated data after Harmony integration, colored by different studies. The Hochgerner dataset is a mouse dataset. **b**, Overview of the age distribution and cell count of the samples included in the studies used in **a**. Left: the distribution of sample ages. Right: the number of cells for each dataset. **c**, UMAP plot colored by the annotated cell types determined through marker gene expression. **d**, UMAP plots illustrating the expression levels of the key marker genes. The color from dark to bright represents the expression level from low to high. **e**, Dot plot showing the marker gene expression across different cell types. **f**, Volcano plot displaying the DEG results (two-sided Wilcoxon test) in putative NPCs compared to all others. The *x* axis indicates the log_2_ fold change in gene expression, while the *y* axis represents the negative logarithm (base 10) of the adjusted *P* values. The red dots represent significant DEGs (Benjamini–Hochberg-adjusted *P* value <0.05 and |logFC| >0.5), while the blue dots represent nonsignificance. The horizontal dashed line is the cutoff of the *P* value. The vertical dashed lines are the cutoff of the logFC. **g**, Bar plot showing the proportion of cells expressing the conserved cross-species NPC marker genes^12^ in different cell types. **h**, Violin plot showing the expression of the conserved cross-species NPC markers across different age groups in the NPCs in human datasets. The child group was removed due to only three cells annotated as NPCs. **i**, UMAP visualization of NPC gene module score, where brighter colors represents higher scores. **j**, Line chart showing the relative percentages of cells that change with the NPC gene module score. The *x* axis shows the NPC gene module score (Methods). For each NPC gene module score, the *y* axis shows the relative percentages of cells, which is the number of cells divided by the total number of putative NPCs. **k**, Confocal image of colocalization of NPC marker ASCL1 (green) and proliferative marker MKI67 (red) within the DG of healthy adult humans (*n* = 2 specimens). Scale bars, 20 μm (overview) and 10 μm (magnification). GCL, granule cell layer. Source data

Most single-cell data of the adult human brain generated from previous studies involve only a few samples on a specific experimental protocol or technology, resulting in disagreement over neurogenesis cell type definitions^14,18–20^. Taking advantage of the large-scale data in the Brain Cell Atlas, we investigated the potential existence of rare NPCs in the adult hippocampus. According to the machine-learning-based annotation, we selected data from five independent human studies including adults^14,15,18,30^, children^14^, infants^14^ and fetuses^13^. To facilitate the cell type annotation by cross-species comparison, the data were integrated with mouse data across all development stages^31^ (Fig. 3a,b) according to orthologous genes. Considering that the adult human data are dominated by mature neurons, we integrated adult human data with fetal and mouse data, which cover the complete neurogenic trajectory.

After data integration using the Harmony^32^ program (Methods), the UMAP visualization shows well-mixed data from the six studies, while the data distribution shows a complete landscape of neurogenesis as well as the enriched mature neurons in the adult samples (Supplementary Fig. 6). The cell clusters were annotated according to the well-established cell type marker genes (Methods and Fig. 3c), including (1) *MKI67* and *TOP2A* for NPCs, (2) *DLX2* and *SOX11* for neuroblast cells, (3) *SOX11* and *PROX1* for immature glutamatergic cells, (4) *PROX1* and *PLEKHA2* for glutamatergic neurons, (5) *SLC17A7* and *COL5A2* for CA neurons, (6) *GAD1* and *GAD2* for GABAergic neurons, (7) *GFAP* and *AQP4* for astrocytes, (8) *FLT1* and *ENG* for endothelial cells, (9) *PDGFRA* and *OLIG1* for oligodendrocyte precursor cells (OPCs), (10) *OLIG2* and *SOX10* for newly formed oligodendrocytes (NFOLs) and (11) *MOG* and *MAG* for oligodendrocytes^12,19^ (Fig. 3d,e). According to proliferative markers (*MKI67* and *TOP2A*) (Fig. 3d,e and Supplementary Fig. 7a,b), only a small proportion of cells (33 cells) in the adult hippocampus as well as 95 fetal and 494 mouse cells can be defined as putative NPCs. When hippocampal tissue sections from male adult macaques aged 7 and 15 years were stained (Supplementary Fig. 7c), we observed coexpression of progenitor cell markers (ASCL1 and SOX2) and glial fibrillary acidic protein (GFAP) in the dentate gyrus (DG). Furthermore, immunostaining of brain sections from individuals aged 6, 7 and 15 years revealed that SOX2^+^MKI67^+^ cells were detected in the subgranular zone (SGZ) of adult macaques (Supplementary Fig. 7d). The DEGs (Methods and Supplementary Table 2) of putative NPCs show some well-established neural progenitor markers^12^, including *TOP2A*, *HMGB2*, *PBK* and *UBE2C* (Fig. 3f and Supplementary Fig. 7e–h). Furthermore, reference-based machine learning methods based on the mouse data^31^ as the reference (Methods) also confirmed these putative NPCs (Supplementary Fig. 8).

**Extended Data Fig. 1:**
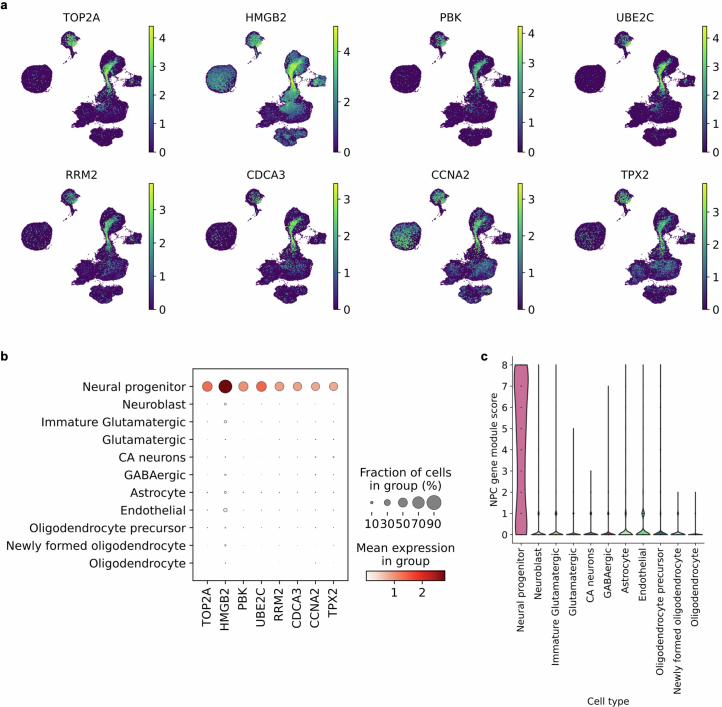
The expression profile of cross-species conserved NPC marker genes. **a**. The UMAP plots show the distribution of normalised expression for each gene. The intensity of the colour indicates the expression level. **b**. The dot plot shows the expression levels of these genes in different cell types. The intensity of the colour indicates the expression level. Size represents the proportion of cells expressing the gene. **c**. The violin plot shows the distribution of NPC gene module scores in different cell types. Source data

Additionally, we investigated NPC gene module score (Methods) analysis to validate putative NPCs on the basis of conserved cross-species NPC markers (*TOP2A*, *HMGB2*, *PBK*, *UBE2C*, *RRM2*, *CDCA3*, *CCNA2* and *TPX*)^12^. Each gene in the NPC gene module score is expressed in approximately half of the putative NPCs but not in the other cell types (Fig. 3g and Extended Data Fig. 1a,b). These genes are lower expressed in adult and infant hippocampus than in fetuses (Fig. 3h). Putative NPCs attained the highest NPC gene module scores against other cell types, indicating that they exhibit the strongest signal of coexpression of conserved cross-species NPC markers (Fig. 3i and Extended Data Fig. 1c). The number of cells coexpressing two or more of these genes decreased sharply in adults, suggesting that putative NPCs in adults may exhibit distinct transcriptional signatures than the ones in fetuses (Fig. 3j and Supplementary Table 3).

**Extended Data Fig. 2:**
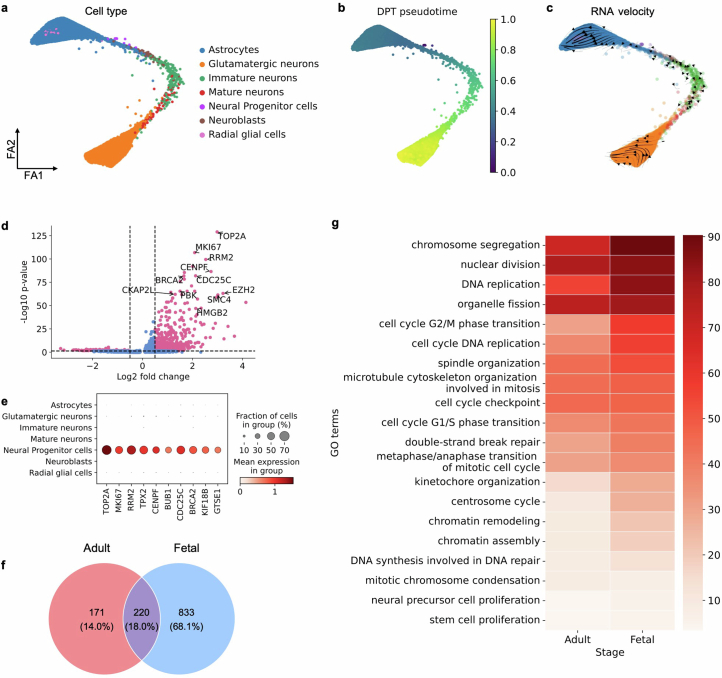
Analysing the putative NPCs in adult humans. **a**. The diffusion map plot shows the trajectory bifurcating to astrocytes and to mature neurons on the diffusion map space, coloured by the annotated cell types. **b**. The diffusion map plot shows the pseudotime calculated by DPT, with the start point at one of the putative NPCs. The intensity of the colour indicates the pseudotime. **c**. RNA splicing velocity changes on the plot of a. shows the inferred developmental trajectory on the plot of the diffusion map, coloured by the annotated cell types. **d**. The volcano plot shows two-sided Wilcoxon test differential genes between putative NPCs and other cells in the adult human hippocampus. The x-axis indicates the log2 fold change in gene expression, while the y-axis represents the negative logarithm (base 10) of the adjusted p value. Red dots represent genes that exhibit a statistically significant differential expression (Benjamini-Hochberg adjusted p-value < 0.05 and |LogFC | > 0.5), while blue dots represent non-significant genes. **e**. The dot plot shows the expression distribution of the top 10 differential genes among different cell types. **f**. The Venn diagram shows the overlap between differentially expressed genes in the putative NPCs from adult and from fetal. **g**. The heatmap compares enriched GO terms in the putative NPCs from adult and from fetal. Source data

Trajectory analysis can also facilitate progenitor identification according to the developmental order of cells inferred from gene expression^33^ or RNA splicing status^34^. We extracted these putative NPCs together with cells in the two bifurcating directions, which are astrocytes and glutamatergic neurons, for trajectory inference (Extended Data Fig. 2a). Both pseudotime analysis and RNA velocity (Extended Data Fig. 2b,c and Supplementary Fig. 9) confirmed the trajectories starting from putative NPCs bifurcating to astrocytes and mature neurons. In adult humans, the DEGs (Extended Data Fig. 2d) of these putative NPCs against other mature cell types validate their cell identity, consistent with the DEGs of putative NPCs derived from fetal humans (Extended Data Fig. 2e,f). Gene Ontology (GO) enrichment analyses showed that putative NPCs mainly participate in neural precursor cell proliferation (*NES*, *FABP7*, *ASCL1* and *KDM1A*), cell cycle regulation (*TOP2A*, *MKI67*, *UBE2C*, *CENPF* and *TPX2*), DNA replication (*CCNA2*, *BRCA2* and *CDK2*), nuclear division (*CENPF*, *SMC4* and *CDC25C*) and chromosome segregation (*SMC1A*, *PLK1*, *MAD2L1* and *AURKB*) (Extended Data Fig. 2g).

For experimental validation, we performed multiple immunostaining assays using antibodies against proliferating neural progenitor marker ASCL1 (ref. ^35^), along with the proliferative marker MKI67. Immunostaining shows that MKI67 colocalized with ASCL1 in the hippocampal DG of healthy adult humans, suggesting the existence of proliferative NPCs (Fig. 3k).

### Identification of *PCDH9*^high^ microglia across brain regions

**Fig. 4:**
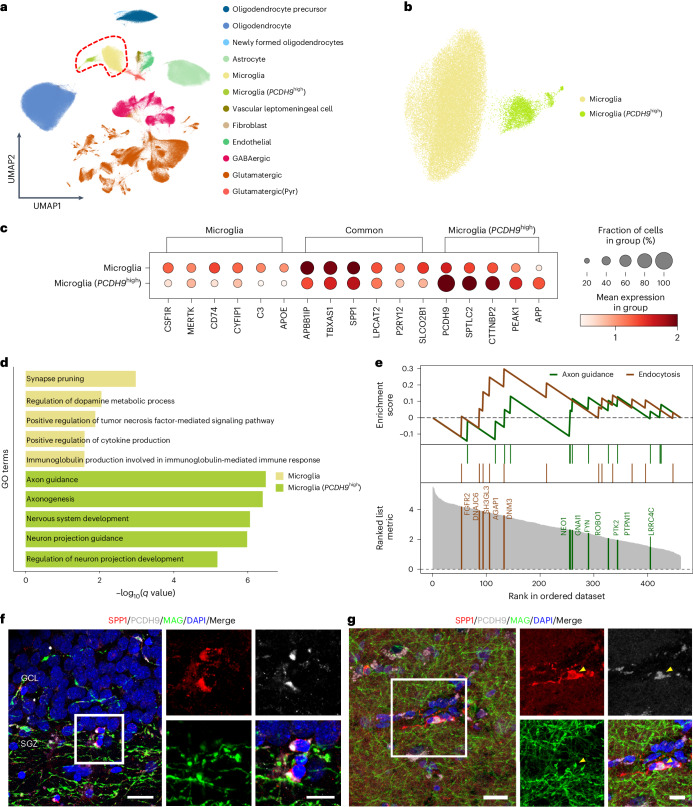
Integrated datasets across brain regions yield a subtype of *PCDH9*-high expressing microglia. **a**, UMAP plot showing the four datasets integrated by harmony integration, with the colors representing the annotated cell type. The red dashed outline indicates microglia and microglia (*PCDH9*^high^) populations. **b**, UMAP plot illustrating a subset highlighted in red circles within **a**, comprising microglia and microglia (*PCDH9*^high^) cells. **c**, Dot plot showing the expression marker genes for microglia and microglia (*PCDH9*^high^). **d**, Bar plot showing the enriched GO terms in microglia and microglia (*PCDH9*^high^) clusters. The analysis was based on Enrichr, using two-sided Fisher’s exact test with Benjamini–Hochberg correction for multiple comparisons. **e**, GSEA visualization of microglia (*PCDH9*^high^) participating in axon guidance and endocytosis pathways. The analysis was based on clusterProfiler, using a two-sided hypergeometric test with Benjamini–Hochberg correction for multiple comparisons. **f**, Triple immunostaining of SPP1 (red), PCDH9 (gray) and MAG (green) confirmed the phagocytosis of myelin debris by microglia (*PCDH9*^high^) in the hippocampal DG (*n* = 2 specimens). Scale bars, 20 μm (overview) and 10 μm (magnification). **g**, Triple immunostaining of SPP1 (red), PCDH9 (gray) and MAG (green) confirming the phagocytosis of myelin debris by microglia (*PCDH9*^high^) in the prefrontal cortex (*n* = 2 specimens). The yellow arrowheads indicate SPP1^+^ PCDH9^+^ MAG^+^ microglia. Scale bars, 20 μm (overview) and 10 μm (magnification). Source data

**Extended Data Fig. 3:**
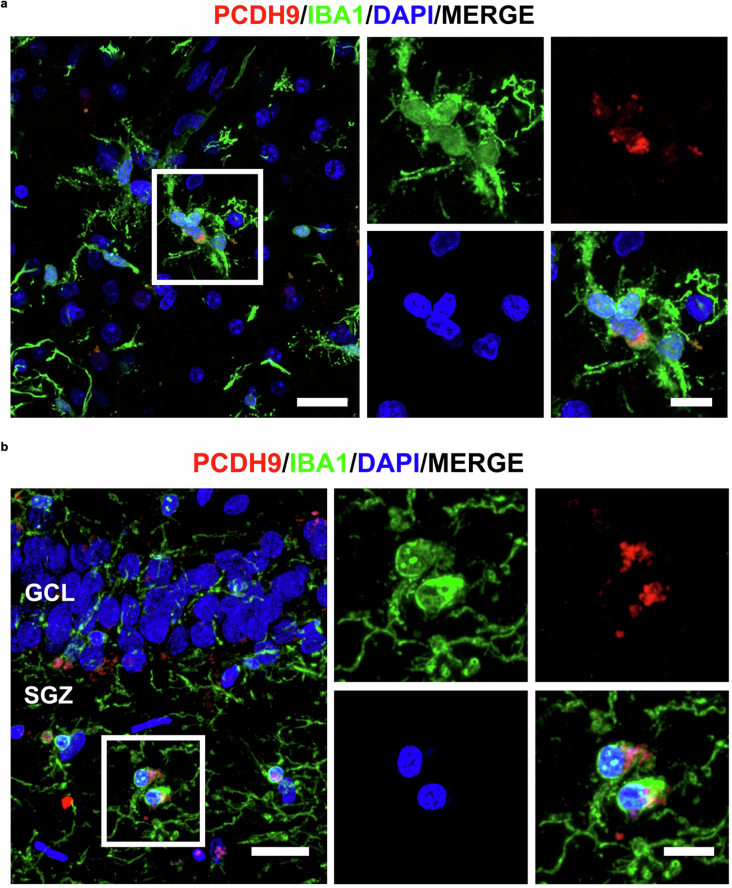
Immunostaining of PCDH9+ microglia in adult human. **a**. Representative immunofluorescence images for PCDH9 and IBA1 in the adult human prefrontal cortex (n = 4 specimens). Scale bars, 20 μm (overview) and 10 μm (magnification). **b**. Representative immunofluorescence images for PCDH9 and IBA1 in the adult human hippocampus (n = 3 specimens). Scale bars, 20 μm (overview) and 10 μm (magnification).

**Extended Data Fig. 4:**
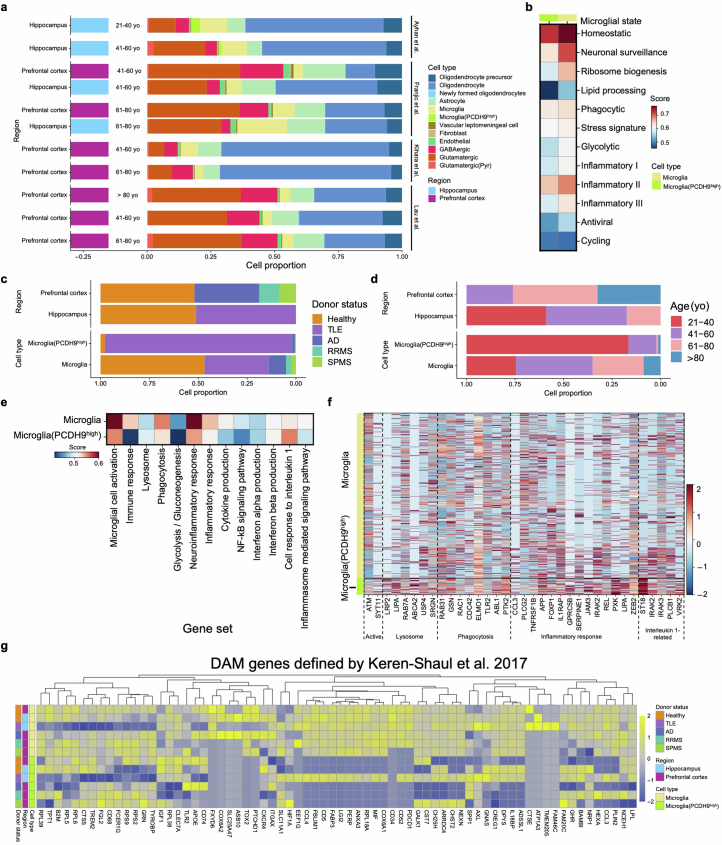
Transcriptional states of microglia (*PCDH9*^high^). **a**. The stacked bar plot shows the metadata of integrated data sets across brain regions. **b**. The heatmap shows gene set scoring of 12 distinct microglial states from Sun et al.^37^. **c**. The stacked bar plot shows the donor status statistics of microglia and microglia (*PCDH9*^high^) clusters. TLE: Temporal lobe epilepsy (Note: Donors exhibited no hippocampal lesion). AD: Alzheimer’s disease. RRMS: Relapsing-remitting multiple sclerosis. SPMS: Secondary progressive multiple sclerosis. **d**. The stacked bar plot shows the cell proportion of microglia and microglia (*PCDH9*^high^) clusters at different ages. **e**. The heatmap shows gene set scoring of predicted biological function. **f**. The heatmap shows the expression of specific gene lists within microglia and microglia (*PCDH9*^high^) clusters. **g**. The heatmap shows the expression of DAM genes (defined by Keren-Shaul et al.^45^) in microglia and microglia (*PCDH9*^high^) across distinct brain regions and diverse disease conditions. The heatmap illustrates the log-transformed average expression of cell types across donor statuses and brain regions.

The unprecedented scale of the resource also facilitates the exploration of cell type diversity. A microglia population with a high level of *PCDH9* expression was identified from the integrated data of 43 samples, covering 511,872 cells. These samples were obtained from four studies of adult human prefrontal cortex and hippocampal regions^17,18,30,36^, providing 12 well-annotated primary cell types (Fig. 4a). Zooming in the microglia cells from the primary cell types, we characterized a novel population of microglia with high *PCDH9* expression (Fig. 4b). We next confirmed the existence of PCDH9^+^IBA1^+^ microglia in healthy adult brains across the prefrontal cortex and hippocampus by double immunofluorescence staining of the corresponding proteins (Extended Data Fig. 3a,b). Furthermore, leveraging DEGs identified in the 12 distinct microglial states by Sun et al.^37^ as a point of reference, our gene set scoring analysis demonstrated that microglia (*PCDH9*^high^) were positioned between a state of homeostasis and inflammatory II (Extended Data Fig. 4a,b). In addition to the microglial markers (*APBB1IP* (ref. ^30^), *TBXAS1*, *SPP1* (ref. ^38^), *LPCAT2* (ref. ^39^), *P2RY12* (ref. ^40^) and *SLCO2B1* (ref. ^41^)), the microglia (*PCDH9*^high^) population also exhibits high expression of immune-related genes (*SPTLC2*, *CTTNBP2* (ref. ^42^), *PEAK1* and *APP*) (Fig. 4c and Extended Data Fig. 4c,d), indicating a functional discrimination in modulating immune responses against other microglia cells. The microglia cluster highly expresses nonhomeostatic marker *APOE*^43^, colony-stimulating factor 1 receptor (*CSF1R*) and phagocytosis receptor *MERTK*^43^ (Supplementary Table 4).

**Extended Data Fig. 5:**
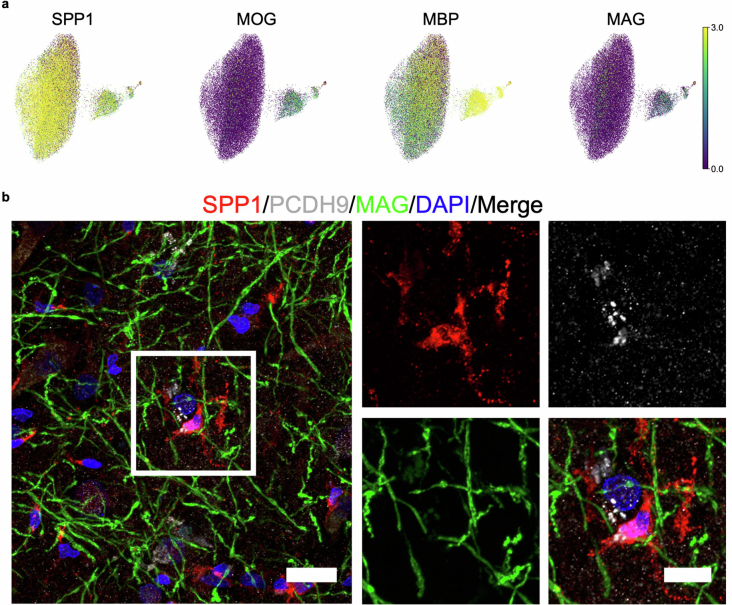
The transcription and protein expression levels for SPP1 and myelin-specific genes. **a**. UMAP plots show the expression of SPP1 and myelin proteins (MBP, MAG, and MOG) in microglia (*PCDH9*^high^). **b**. Triple immunostaining of SPP1 (red), PCDH9 (grey), and MAG (green) confirmed the phagocytosis of myelin debris by microglia (*PCDH9*^high^) in the cornu ammonis of the hippocampus (n = 1 specimen). Scale bars, 20 μm (overview) and 10 μm (magnification).

The GO terms of microglia (*PCDH9*^high^) against microglia (Supplementary Table 5) indicated that microglia might be engaged in synapse pruning, regulation of dopamine metabolic process and positive regulation of cytokine production. Yet, microglia (*PCDH9*^high^) might be involved in axon guidance, axonogenesis, nervous system development, neuron projection guidance and regulation of neuron projection development (Fig. 4d). Gene set enrichment analysis (GSEA) confirmed the involvement of microglia (*PCDH9*^high^) in axon guidance and endocytosis pathways (Fig. 4e). *SPP1*, defined as a molecular signature in axonal tract-associated microglia^21,44^, exhibits notable expression level in microglia (*PCDH9*^high^), suggesting that they may congregate around axon tracts. Surprisingly, myelin proteins (MBP, MAG and MOG) were detected in microglia (*PCDH9*^high^) (Extended Data Fig. 5a). Multiple immunostaining showed that PCDH9 colocalizes with the typical microglial activation gene *SPP1* in the prefrontal cortex and hippocampus region of healthy adult human brains (Extended Data Fig. 5b). Additionally, immunostaining showed that PCDH9^*+*^SPP1^*+*^ microglia are intermingled with myelin-associated glycoprotein (MAG) in the prefrontal cortex and hippocampus region (Fig. 4f,g and Extended Data Fig. 5b), suggesting that microglia (*PCDH9*^high^) may engulf myelin debris, potentially contributing to the maintenance of physiological axon myelination.

As *SPP1* is known to be related to disease-associated microglia (DAM)^22,45^, we investigated the relationship between this microglia (*PCDH9*^high^) population and DAM. Although microglia (*PCDH9*^high^) cells express high *SPP1*, they show different expression patterns for the DAM activation genes (*TREM2*, *APOE*, *TYROBP*, *CST7* and *LPL*). Activated microglia (*PCDH9*^high^) cells exhibit elevated expression of lysosomal-associated genes, along with phagocytic phenotypes (Extended Data Fig. 4e,f), but demonstrate a limited association with DAM signatures (Extended Data Fig. 4g). Taken together, microglia (*PCDH9*^high^) might concentrate around the axon, correlating with immune cell activation, lysosomal activity and phagocytic processes.

### Regional microenvironment drives microglial heterogeneity

**Fig. 5:**
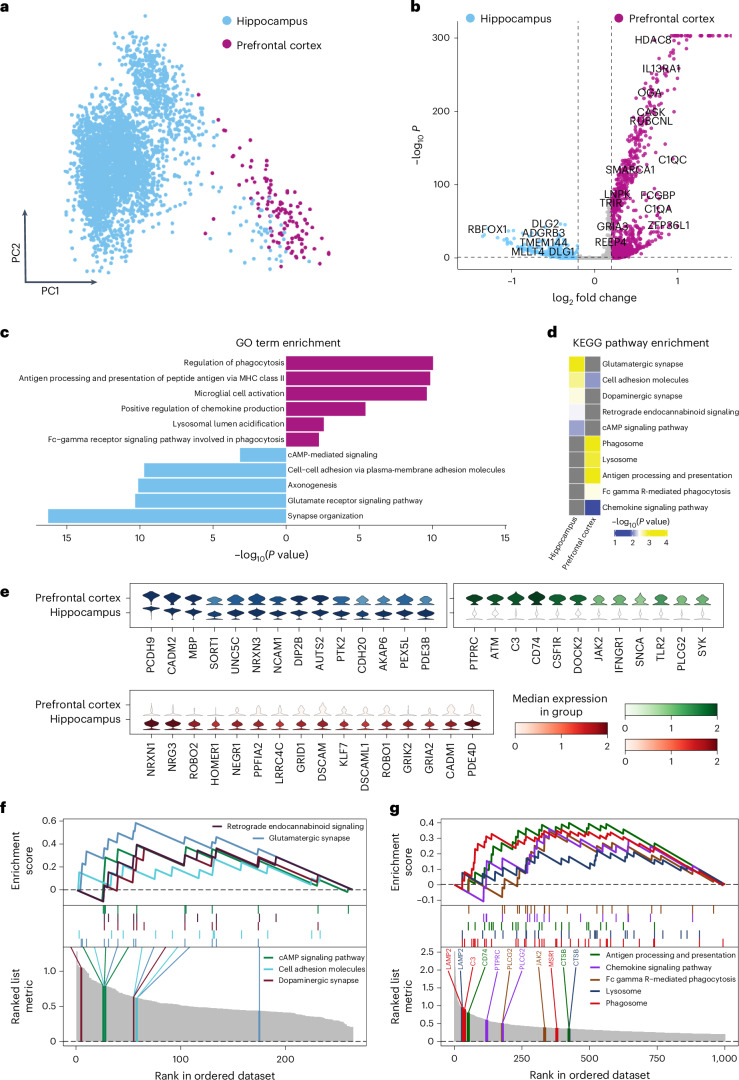
Regional transcriptional identities of microglia (*PCDH9*^high^). **a**, PCA plot showing microglia (*PCDH9*^high^) cells according to the hippocampus and prefrontal cortex. The cells were colored by brain region. **b**, Volcano plot showing that there were 1,469 DEGs between hippocampus and prefrontal cortex (adjusted *P* value <0.05). The horizontal dashed line is the cutoff of the *P* value. The vertical dashed lines are the cutoff of the logFC. **c**, Bar plot showing enriched GO terms in the microglia (*PCDH9*^high^) cells from hippocampus and prefrontal cortex. **d**, KEGG pathway enrichment analysis of microglia (*PCDH9*^high^) cells from hippocampus and prefrontal cortex. **e**, Common and specific transcriptional features of microglia (*PCDH9*^high^) in the hippocampus and prefrontal cortex. **f**, GSEA result of gene sets from hippocampal microglia (*PCDH9*^high^). **g**, GSEA result of gene sets from microglia (*PCDH9*^high^) in the prefrontal cortex. The analysis in **c** and **d** was based on clusterProfiler, using a two-sided hypergeometric test with Benjamini–Hochberg correction for multiple comparisons. Source data

The same cell population may demonstrate different gene regulatory patterns in different microenvironments, and understanding such a niche difference may help the development of in vitro cell culture protocols or technologies^46^. Single research group datasets may be limited in size or confounded in experimental design, hindering the understanding of microenvironment-related differences, especially cross-brain region effects. In large-scale atlas data, a gene covarying exclusively with brain regions, not sequencing batches or studies, is likely to be region specific rather than batch specific (Supplementary Fig. 10). Under this assumption, we performed differential expression analysis for the above-mentioned microglia (*PCDH9*^high^) population across two brain regions, prefrontal cortex and hippocampus (Fig. 5a and Supplementary Table 4).

**Extended Data Fig. 6:**
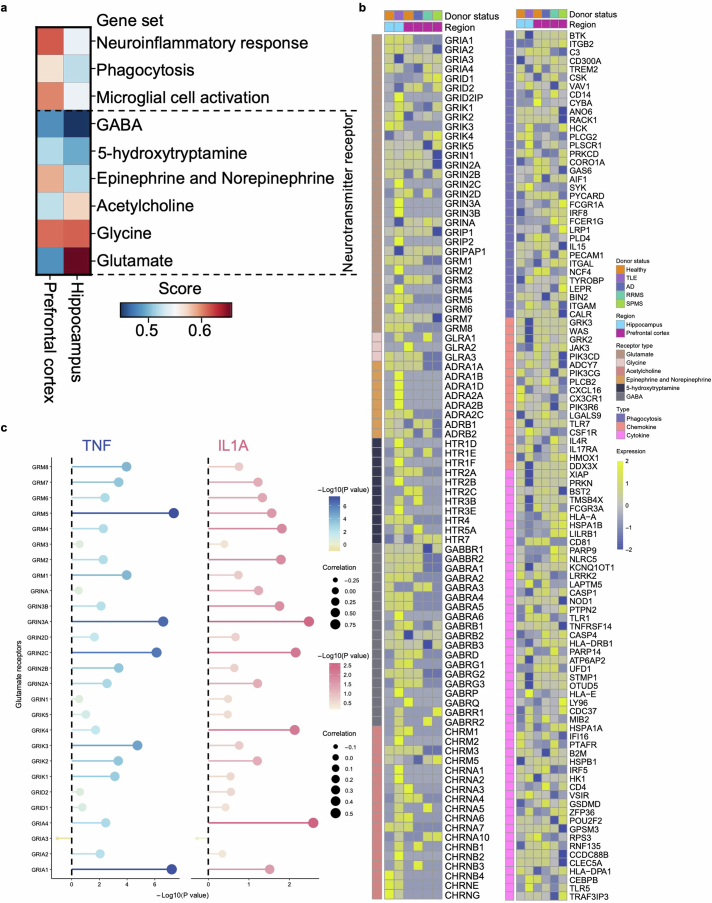
Microglia (*PCDH9*^high^) regional heterogeneity at activated state. **a**. The heatmap shows gene set scoring of regional microglia (*PCDH9*^high^) from MSigDB. **b**. The average expression levels of neurotransmitter receptors (left), phagocytic, chemotactic, and cytokine-related genes (right) in regional microglia (*PCDH9*^high^) across healthy and diverse disease conditions. **c**. Result of two-sided Pearson correlation analysis between TNF (left) and IL1A (right) with glutamatergic microglial receptors. Pearson correlation coefficients and corresponding p-values are shown. Source data

Regional characteristics of the microglia (*PCDH9*^high^) show that complement components (*C1QA* and *C1QC*) are highly expressed in the prefrontal cortex, while microglial cell activation gene *DLG1* (ref. ^47^) is highly expressed in the hippocampal region (Fig. 5b). Enriched GO analysis revealed advanced phagocytic scavenging capacity (*C3* and *PLCG2*), antigen presentation (major histocompatibility complex (MHC) class II genes) and immune response (*CD74*, *TLR2* and *SYK*) in microglia (*PCDH9*^high^) of the prefrontal cortex, while hippocampal microglia (*PCDH9*^high^) exhibits associations with the regulation of excitatory synapse plasticity (*NRXN1*, *GRIK2* and *HOMER1*) (Fig. 5c). Kyoto Encyclopedia of Genes and Genomes (KEGG) pathway enrichment analysis validates the biological process of microglia (*PCDH9*^high^) regional heterogeneity (Fig. 5d), with genes involved in phagocytosis and modulating synaptic plasticity enriched specifically in the prefrontal cortex and hippocampus regions, respectively (Fig. 5e and Supplementary Table 5). Consistently aligning with the GO term, KEGG enrichment and GSEA (Fig. 5f), hippocampal microglia (*PCDH9*^high^) exhibit a high-level expression of glutamate receptors (*GRIA2*, *GRIK2* and *GRIK3*), suggesting its potential involvement in bidirectional interactions with excitatory neurons (Extended Data Fig. 6a,b). Compared to the hippocampus, these analytical outcomes substantiate a heightened proinflammatory and phagocytic state of microglia (*PCDH9*^high^) in the prefrontal cortex (Fig. 5g). Pearson correlation demonstrates a positive trend between the inflammatory cytokines tumor necrosis factor (*TNF*), interleukin-1α (*IL1A*) and glutamate ionotropic/metabotropic receptors (excluding *GRIA3*) (Extended Data Fig. 6c), indicating a potential regulation by glutamatergic neurons on the activation, phagocytic activity and phenotypic differentiation of microglia (*PCDH9*^high^).

**Fig. 6:**
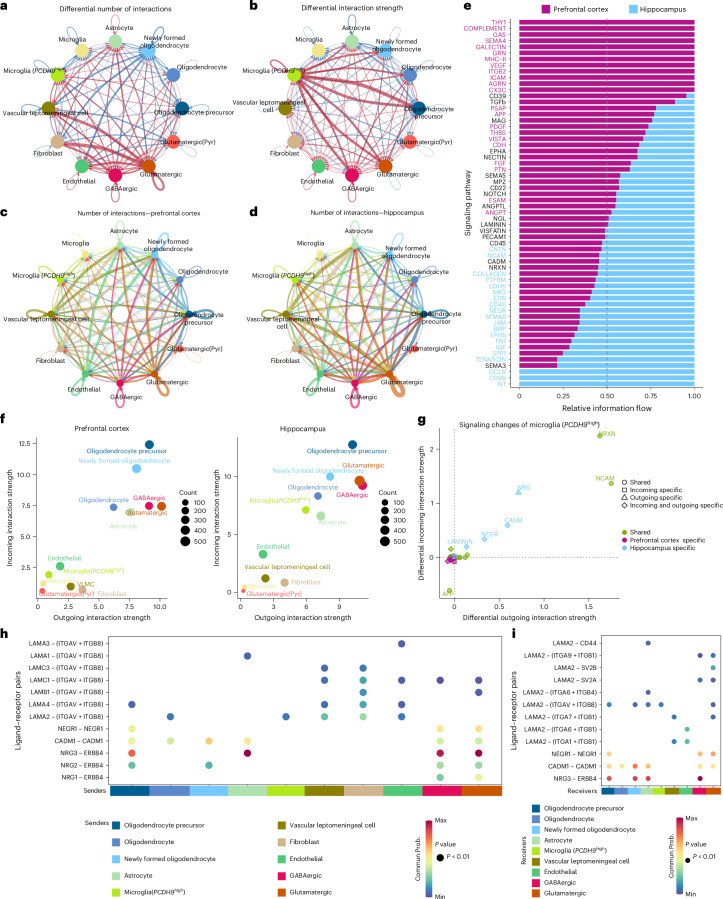
Cell–cell communication of the neurogenic niches in the prefrontal cortex and hippocampus. **a**, Circle plot showing the number of interactions and the strength of interactions among different cell types. The red (blue) colored edges represent increased (decreased) signaling in the hippocampus compared to the prefrontal cortex region. **b**, Circle plot showing the differential interaction strength among different cell types. The red (blue) colored edges represent increased (decreased) interaction strength in the hippocampus compared to the prefrontal cortex region. **c**, Circle plot displaying the number of interactions and the strength of interactions between any two cell groups in the prefrontal cortex region. The number of lines represents the number of interactions, and the thickness of the lines is proportional to the strength of the interactions. **d**, Circle plot displaying the number of interactions in the hippocampus. The number of lines represents the number of interactions, and the thickness of the lines is proportional to the strength of the interactions. **e**, Stacked bar plot showing the overall information flow of each signaling pathway. The vertical dashed line indicates the position where the sample accounts for 50% of the overall information flow. **f**, Scatter plot showing dominant senders and receivers in a 2D space, showing the prefrontal cortex (left) and the hippocampus region (right). **g**, Scatter plot demonstrating the signaling changes associated with microglia (*PCDH9*^high^) cell groups in the prefrontal cortex and hippocampus. **h**, Dot plot displaying the expression of significant ligand–receptor pairs in the NRG, CADM, NEGR and laminin pathways from all senders to hippocampal microglia (*PCDH9*^high^). *P* values are computed from a one-sided permutation test according to CellChat. **i**, Dot plot showing the expression of significant ligand–receptor pairs in the NRG, CADM, NEGR and laminin pathways from hippocampal microglia (*PCDH9*^high^) to cell receivers. *P* values are computed from a one-sided permutation test according to CellChat. Commun.Prob., communication probability. Source data

**Extended Data Fig. 7:**
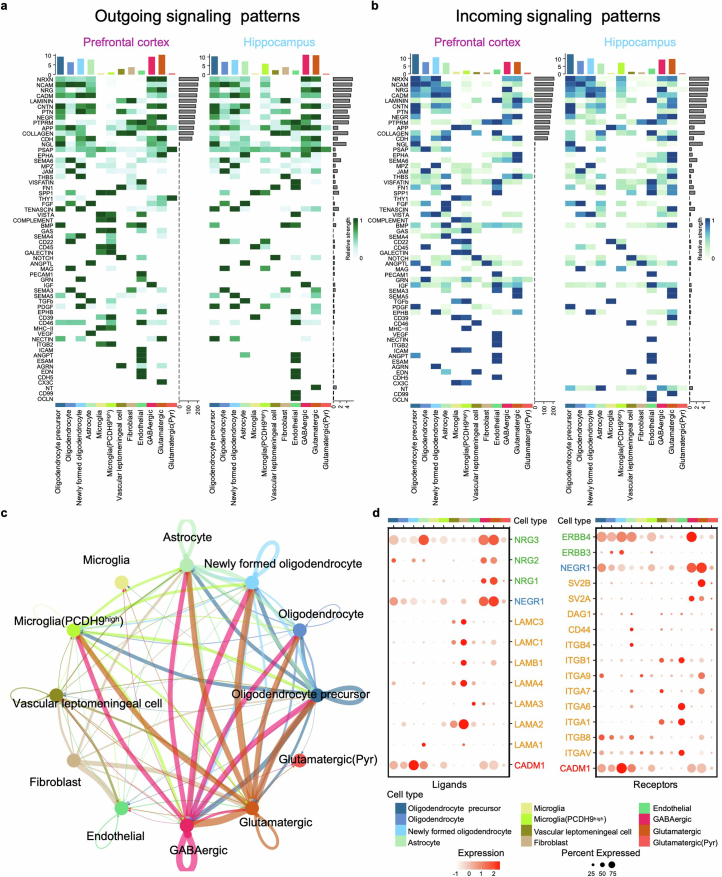
Cell-cell communication network in the prefrontal cortex and hippocampus region. **a**. The comparison heatmap shows the patterns of outgoing communication. **b**. The comparison heatmap shows the patterns of incoming communication. **c**. The circle plot displays the interaction network of NRG, CADM, NEGR, and LAMININ pathways in the hippocampus region. **d**. The dot plot highlights the expression levels of significant ligand-receptor pairs associated with the NRG, CADM, NEGR, and LAMININ pathways in different hippocampal cell types. Green, blue, yellow, and red fonts denote genes from the NRG, CADM, NEGR, and LAMININ pathways, respectively.

To further elucidate the bidirectional neuronal–microglial (*PCDH9*^high^) communication, we employed the CellChat program^48^ to explore potential ligand–receptor interactions (Fig. 6a–d and Supplementary Table 6). Overall, 60 pathways (980 genes) were involved in building the cell–cell communication network of the neural cell niches, including 45 conserved pathways, 12 prefrontal cortex-specific pathways and 3 hippocampal-specific pathways (Fig. 6e). As shown in Fig. 6f and Extended Data Fig. 7a,b, the distribution of cells in two-dimensional (2D) space shows changes in the interaction strength of outgoing and incoming signaling between the microglia (*PCDH9*^high^) cells in prefrontal cortex and hippocampus. Furthermore, the hippocampal microglia (*PCDH9*^high^) shows distinctive signaling alterations, characterized by the specific changes in the neuregulin (NRG), cell adhesion molecule (CADM), neuronal growth regulator (NEGR) and laminin pathways (Fig. 6g).

In the hippocampus, ten cell senders, which secrete ligands, interact with the microglia (*PCDH9*^high^) cell population via the NRG, CADM, NEGR and laminin pathways mediated by multiple ligand–receptor pairs (Fig. 6h). Nine cell receivers interact with microglia (*PCDH9*^high^) when it acts as a signal sender: oligodendrocyte precursor cells, oligodendrocytes, NFOLs, astrocytes, vascular leptomeningeal cells, fibroblasts, endothelial cells, GABAergic neurons and glutamatergic neurons (Fig. 6i and Extended Data Fig. 7c). Although the functional role of most ligand–receptor pairs in microglia remains elusive, some ligand–receptor pairs expressed on hippocampus-enriched cell types (Extended Data Fig. 7d). LAMB1–ITGB8 may serve as a specific ligand–receptor pair for glutamatergic neuronal-to-microglial (*PCDH9*^high^) communication, whereas LAMA1–SV2B, LAMA2–(ITGAV + ITGB8) and LAMA2–(ITGA7 + ITGB1) might function as specific ligand–receptor pairs for microglial (*PCDH9*^high^)-to-neuronal communication. These differential ligand–receptor pairs suggest that the microglia (*PCDH9*^high^) population selectively prunes glutamatergic neurons. Collectively, we present a cell–cell communication network in the prefrontal cortex and hippocampus, enhancing the understanding of the neuronal–microglial crosstalk pathways.

## Discussion

We present here the Brain Cell Atlas including 26.3M cells or nuclei from human and mouse tissues covering 173 studies. A large-scale integrated atlas from diverse sources can effectively address the limitations of individual datasets, enabling the discovery of rare cell types and regional variations. For example, we demonstrated the identification of putative NPCs in the adult human hippocampus and microglia regional heterogeneity using our data resource.

There have been an ongoing debate and conflicting findings regarding adult hippocampal neurogenesis (AHN)^14,20,49–51^. We used several approaches to infer putative NPCs, including marker gene identification, experimental immunostaining, gene module scoring, trajectory inference and cross-species comparison based on well-annotated mouse data. Putative NPCs express transcripts related to molecular hallmarks of proliferating neural progenitors, such as acknowledged NPC markers (*SOX2*, *NES*, *ASCL1*, *EOMES*, *FABP7*, *PBK* and *PAX6*)^14,35,49,52^, and proliferative genes (*MKI67*, *TOP2A*, *PCNA* and *CCND2*)^20,50^ and adult granule cell lineage marker *PROX1* (ref. ^53^). Integrated cross-species analysis, unsupervised clustering and trajectory inference highlighted a track of putative NPCs from the neurogenic lineage. MKI67 was validated as a proliferating marker for NPCs in human adults by immunostaining. Increasing evidence supports the existence of AHN^14,20,49,50^, and still more experimental evidence is required to assess the extent to which similarities exist between mouse and fetal NPCs. By pooling data from multiple studies, the discovery of adult putative NPCs provides a preliminary molecular foundation for the development of novel therapies targeting neurological injuries and neurodegenerative diseases.

We have identified microglia (*PCDH9*^high^) within the adult prefrontal cortex and hippocampal region exhibiting a proinflammatory phenotype and the potential engulfment of myelin debris. Previous works have demonstrated that the TREM2–APOE pathway initiates the transformation of DAM in neurodegeneration models^54,55^. Contrary to DAM, the phagocytic capacity of microglia (*PCDH9*^high^) toward myelin debris appears independent of *TREM2* and *APOE* expression. We observed that *SPP1* (associated with immune cell activation, lysosomal activity and phagocytosis^38,47^) is also expressed in microglia (*PCDH9*^high^). So far, several studies have reported the function of SPP1-positive microglia in engulfing myelin debris^38,44,56,57^, with the difference that microglia (*PCDH9*^high^) seem to be independent of lipid metabolism and proliferation. Li et al.^38^ discovered Spp1^+^ proliferative-region-associated microglia interspersed with Mbp^+^ oligodendrocytes. Microglia (*PCDH9*^high^), as well as CD11c^+^Spp1^+^ microglia^57^ and Spp1^+^ axon tract-associated microglia^44^, exhibit transcriptional features associated with immune cell activation, lysosomal activity and phagocytosis highly similar to Spp1^+^ proliferative-region-associated microglia. Growing evidence underscores regional microglial heterogeneity, implicating microenvironmental disparities as primary contributors^22^, and we indicate a potential bidirectional communication mechanism between hippocampal microglia (*PCDH9*^high^) and glutamatergic neurons. These findings present an exciting possibility that regional differences in synaptic plasticity, myelination and the activity of diverse neuronal subtypes (excitatory or inhibitory) might require distinct microglial functions.

However, integrating large-scale atlases still presents computational challenges, such as modeling the batch effects and reducing technical noise. Batch correction for large-scale data in the expression matrix is computationally expensive, and performing differential expression analysis without accounting for batch effects may lead to bias. A more efficient approach reported recently^58,59^ is to model both biology and batch effects in differential expression analysis after data integration. Yet, certain biases for batch effects may still be difficult to avoid in differential expression analysis when the experimental design is confounded. Besides, high dropout rates and ambient RNA contamination may be sources of technical noise. Although the single-cell technology is at single-cell resolution, some gene expressions are not expected in all cells of the cluster due to the dropouts. As the hippocampal microglia (*PCDH9*^high^) express phagocytosis- and lysosome-related genes and some neuronal genes, more validations could be required to discriminate potential ambient RNA contamination from cellular functions (for example, phagocytosis activity and activation of endogenous gene expression).

As a data resource in the Human Cell Atlas, the Brain Cell Atlas is provided as a web portal with interactive data visualizations and explorations. It provides a unified single-cell reference atlas and data resource at scale, which will facilitate the exploration of unsolved problems in neuroscience and brain disease.

## Methods

### Ethics approval and consent

All public datasets in this manuscript have published ethics approvals. The ethics approval information per study has been summarized in Supplementary Table 7. The deidentified human tissue collection and protocols for the immunostaining assays were approved by the Ethics Committee of the Sun Yat-sen University Cancer Center (SL-B2023-003-02). Written informed consent was obtained from all participants. For macaques, ethical compliance was ensured and all experimental procedures were approved by the Animal Care and Use Committee of Zhongshan Ophthalmic Center at Sun Yat-sen University. The study was performed in accordance with the Principles for the Ethical Treatment of Non-Human Primates. Adult macaques were obtained from Blooming-Spring Biotechnology or were generously gifted from nearby laboratories at Sun Yat-sen University for terminal experiments.

### Statistics and reproducibility

No statistical methods were used to predetermine sample sizes. No data were excluded from the analyses. No randomization was used in our study. The Investigators were not blinded to allocation during experiments and outcome assessment.

### Data collection and curation

We collected single-cell transcriptomic data of 70 human brain studies and 103 mouse brain studies. Data were downloaded from GEO (https://www.ncbi.nlm.nih.gov/geo/), CELLxGENE (https://cellxgene.cziscience.com/), the UCSC genome browser (https://genome.ucsc.edu/), ArrayExpress (https://www.ebi.ac.uk/arrayexpress/) and others. Raw counts were converted into the h5ad format defined Anndata (v0.8.0), using SCANPY (v1.9.1)^60^ and in Python (v3.10.6). Seurat (v4.1.1)^61^ objects were converted into the h5ad format via Sceasy (v0.0.7) (https://github.com/cellgeni/sceasy) in R (v4.0.2). All datasets were saved as sparse matrices, while a few processed datasets were converted into raw counts using scDenorm (v0.0.9) (https://pypi.org/project/scDenorm/).

All metadata were manually curated into a consistent naming (Supplementary Table 1). The metadata, if available in the original publication, include information such as cell properties, source information, brain regions of sampling, sequencing technology, original cell type annotation, demographic information of donors (including disease status), the identifier of the original data source, and the project code. Terms in metadata are defined as fields.

In metadata, ‘cell_ID’ is defined as the index of the sequencing files. The ‘donor_ID’ provides information that uniquely identifies the donors. The ‘donor_sex’ field contains self-reported sex for postnatal or adult donors, while for fetal samples, sex was classified on the basis of information from the original data (Supplementary Table 8). The ‘donor_age’ is classified as months for donors younger than 1 year and as years for postnatal donors older than 1 year. Fetal samples are defined by GW, and organoids are defined by culture days. The ‘donor_status’ and ‘sample_status’ fields indicate the disease status of the donors and the disease status of a cell, respectively. The disease names follow the common names in MONDO Diseased Ontology^62^, while the ‘if_patient’ field states if the donor is healthy or not. The ‘original_name’ field contains the cell types provided in the source, while ‘original_name2’ represents the finer-level cell types. The ‘region’ field denotes the brain region from which the cells were collected. The naming of regions follows a hierarchical structure based on the anatomical regions. The names were curated in a consistent format considering the frequency of occurrence. Typically, the region names are at level 1 (Supplementary Fig. 3). The ‘subregion’ field specifies the finer-level region based on the smallest region stated in the original source. The ‘treatment’ field describes the personal medical treatment, or experimental treatment for organoids. The ‘ethnicity’ field indicates the self-reported donor ethnicity. The ‘seq_method’ field describes the sequencing method. The project codes refer to the data retrieval code of GEO or ArrayExpress. The ‘sample_ID’ field contains GSM IDs from GEO, or the samples are named by ‘author_year’ plus the batch key from the publication. The ‘reference’ field contains the DOI of the publication or a link to the data.

### Quality control

All cells with available cell type annotations from the original publications were retained and skipped for quality control, except those labeled as doublets. Expression profiles sequenced by 10x Genomics without available cell type information went through quality control by removing cells with fewer than 200 counts. Scrublet (v0.2.3)^63^ was used to predict doublets, while cells with doublet scores >0.3 were excluded. Additionally, cells with mitochondrial contents greater than 10% were excluded.

### Reference-based cell type annotation at atlas scale

The collected datasets are categorized into four sample types: adult, fetal, organoids and tumor. These datasets were merged on the basis of sample types. Subsequently, the cell types were reannotated using seven supervised machine learning methods: ACTINN (v1.0.0)^64^, scArches (v0.5.5)^65^, CHETAH (v1.9.0)^66^, scmap (v1.16.0)^67^, SingleCellNet (v0.4.1)^68^, SingleR (v1.8.1)^69^ and scPred (v1.9.2)^70^, as well as an in-house built tools scAnnot (https://github.com/rnacentre/scAnnot). Two well-established datasets were used as references. The Siletti et al.^10^ dataset (of 3M cells) represents the adult brain cells and was used to annotate adult, organoid and tumor datasets. The Braun et al.^11^ dataset was used to annotate fetal data. To account for batch effects in machine learning methods, the ‘sample_ID’ field was used as the batch key. The most frequently annotated cell types from these eight methods were designated as the consensus cell types (labeled as ‘cell_type’). If no single label is predicted by more than half the methods, the cell is labeled as ‘unannotated’. Differential expression analysis was conducted on the ‘cell_type’ parameter using the FindMarker function from Seurat (v4.1.1)^61^ using the Wilcoxon test.

### Hierarchical cell type annotation based on scANVI

To annotate cell types at different resolutions, we developed scAnnot by applying a hierarchical structure to train machine learning models of scANVI (scvi-tools v0.20.3). It first annotates the primary cell types (or cell classes), which can be discriminated by well-defined cell type markers. Subsequently, it identifies specific cell types in each cell class using scANVI trained on reference data of the class. We selected 1,841 representative genes derived from differential expression analysis. scAnnot was trained using raw counts of these genes.

For the hierarchical annotation in scAnnot, an scVI model^71^ was first trained on the training data with five epochs. The reference dataset was split into training and validation sets (5:1). Transfer learning was then employed to fine-tune the scANVI model using the parameters of the pretrained scVI model in cell class annotation. The best model was selected by exploring different hyperparameters, including the latent space dimension (10–100), network layers (1–10) and initializations (10 different seeds). Thirty-one cell class models were trained for the second-level annotation. This approach can be applied to the third level if necessary.

### Atlas-level data integration of all datasets

To integrate datasets (for example, integrating adult datasets), we used scANVI to infer the latent space. So, the datasets can be integrated in this latent space and visualized in UMAP.

The scANVI model training included two layers with a latent space of 50 dimensions and employed negative binomial likelihood for gene expression modeling. To prevent overfitting during unsupervised training, an early-stopping strategy based on the evidence lower bound metric was implemented. The model’s best state, determined by the evidence lower bound metric, was saved. Training is stopped if the metric does not improve for five epochs (threshold 0 for triggering a stop). Additionally, a learning rate reduction strategy was implemented when the loss function plateaued, with a patience of eight epochs and a reduction factor of 0.1. In semi-supervised training, we applied the early-stopping approach focusing on classification accuracy, with the best-accuracy model saved. The scANVI model was trained on the whole reference dataset, with the patience and threshold for early-stopping set to five epochs and 0.001, respectively. Learning rate reduction on plateau was enforced with a patience of eight epochs and a reduction factor of 0.1. scANVI was then applied to datasets for integration and obtained their latent embeddings, which were utilized for downstream analyses.

### Silhouette score evaluating data integration

We used the Silhouette score^29^ to measure the discrimination of covariates, including sequencing technology, donor sex and donor status, in each cell type. For each cell, sklearn.metrics.silhouette_score function from sklearn was used to calculate the silhouette coefficient based on the batch-corrected latent embeddings. The silhouette coefficient is calculated using the mean intracluster euclidean distance (*a*) and the mean nearest-cluster distance (*b*) for each cell. The silhouette coefficient for a sample is (*b* − *a*)/max(*a*, *b*). The silhouette score for a cluster is the average score of the cells. A low silhouette score indicates that the data are unlikely to be driven by the covariate.

### Identification of putative NPCs in the adult human hippocampus

For human hippocampus data, we included single-nucleus or single-cell data from adult human^14,15,18,30^, children^14^, infancy^14^ and fetuses^13^. These samples involved 12 females and 20 males, with their sex assigned either by the medics or by self-report (Supplementary Table 7). These datasets were selected according to machine learning-based annotation.

A cross-species comparison approach was used to annotate human data based on mouse data^31^. Here, human and mouse data were first integrated in the latent space, and the human data are annotated using the logistic regression model^28^ trained on the mouse data in the latent space. A total of 13,596 orthologous genes between human and mouse according to the Ensembl database were selected to combine both datasets, while SCANPY^60^ (v1.9.1) was used for analysis. To focus on neurogenesis, Astro-adult, Astro-juv, Immature-Astro, radial glia-like cell (RGL), RGL_young, neural intermediate progenitor cell (nIPC), nIPC-perin, neuroblast, immature-GC (where ‘GC’ is granule cell), GC-juv and GC-adult from mouse fetal hippocampus data^31^ were used, while nIPC-perin and nIPC were considered as NPC. Principal component analysis (PCA) was performed on the 2,000 highly variable genes selected from the fetal mouse hippocampus single-cell RNA sequencing. Then, the latent PCA space was corrected by Harmony^32^ using ‘species’ and ‘sample ID’ as batch keys. Using the batch-corrected latent space and cell type annotation, a logistic regression model was trained, and this model was applied to infer the cell types of the human data based on batch-corrected latent space.

To optimize the cell type annotation inferred from cross-species comparison, these cell clusters were validated by marker genes. The cells were clustered by Leiden clustering and visualized on UMAP. The well-established cell type markers^12,19^, known in both human and mouse, include *MKI67* and *TOP2A* for neural progenitor, *DLX2* and *SOX11* for neuroblasts, *PROX1* and *PLEKHA2* for glutamatergic, *SLC17A7* and *COL5A2* for neuron, *GAD1* and *GAD2* for GABAergic, *GFAP* and *AQP4* for Astro, *FLT1* and *ENG* for endothelial, *PDGFRA* and *OLIG1* for oligodendrocyte precursor, *OLIG2* and *SOX10* for NFOLs and *MOG* and *MAG* for oligodendrocyte.

NPC gene module score is defined by the coexpression of cross-species-conserved NPC markers (*TOP2A*, *HMGB2*, *PBK*, *UBE2C*, *RRM2*, *CDCA3*, *CCNA2* and *TPX*) in cells as provided by Tosoni et al.^12^. This score indicates the number of markers detected in a cell with at least one unique molecular identifier. The cell number decreased quickly when the NPC gene module score was less than 3, and then the change tended to be slow. Therefore, putative NPCs are defined with an NPC gene module score of ≥3.

To validate the putative NPCs, lineage tracing approaches, including pseudotime analysis and RNA velocity, were used to infer the development trajectories. In pseudotime analysis, the standard SCANPY workflow was used. Specifically, the scanpy.tl.draw_graph function in SCANPY built the graph for visualization and the scanpy.tl.dpt function infers the pseudotime, with one of the putative NPCs set as the root. RNA velocity analysis focused on the neurogenesis-related populations, including cells from putative NPCs to mature neurons. The default scVelo^34^ analysis workflow was applied on the adult human data. RNA velocity (v0.17.17)^72^ was run on the 10x Cell Ranger result using the ‘run10x’ option. The resulting loom files were merged with the AnnData in SCANPY and analyzed with scVelo (v0.2.5). The scvelo.tl.recover_dynamics function in scVelo with default setting was used to recover the full splicing kinetics of the genes. The velocities were estimated by the scvelo.tl.velocity function with dynamical mode. The velocity graph was calculated by the scvelo.tl.velocity_graph function with default parameters.

### Differential expression analysis across regions and cell types

Considering that data integration is performed only in the latent space leaving the raw expression profiles (the log-normalized counts) untouched, differential expression analysis needs to account for both biological variance and unwanted covariates such as batch effects. Considering the necessity to model technical effects along with biological essence, differential expression analysis is performed on aggregating counts data^58,59^, while covariates are modeled via the edgeR (v4.0.1)^73^ program. Similar approaches, which can effectively model batch effects, have been reported and benchmarked before^58,59^. Aggregating the counts can alleviate the dropout issue in single-cell experiments while modeling the covariates can regress out the batch effects and highlight the biological difference. Specifically, counts first are aggregated within each sample (as pseudobulk) using the Libra R package (v1.0.0)^58^. Subsequently, the edgeR’s generalized linear model with likelihood ratio test is employed to model both biological factors (for example, cross-brain region effects) and covariates (for example, batch effects). Donor ID encompasses multiple donor characteristics, including brain regions, sequencing techniques, sex and so on, since each donor typically corresponds to only one brain region, sequencing technique and sex. We use donor ID as covariates to represent batch effects in differential expression analysis (for example, setting the experimental design parameter in edgeR as design = model.matrix(~donor_ID + group), where donor_ID is used to represent the batch effects and group represents the biology design), except when it is confounded^74^ (colinear) with the biology design (for example, setting the experimental design parameter in edgeR as design = model.matrix(~donor_age + donor_sex + group), where donor_age and donor_sex are used to represent the batch effects). When comparing putative NPCs with other cells and comparing microglia (*PCDH9*^high^) with other microglia populations, donor ID was used to represent the batch effect. For cross-brain region comparisons of microglia (*PCDH9*^high^), donor age and sex were used as covariates owing to confounding of donor ID with biological design (brain region). In the differential expression analysis between microglia and microglia (*PCDH9*^high^), 18 female and 25 male samples were included as in Supplementary Table 7. For the visualization of the differential expression results, infinite values were removed from the results, and volcano plots were made with the EnhancedVolcano package (v1.12.0)^75^.

### Gene functional enrichment analysis

The analyses of gene functional enrichment encompassing GO terms and KEGG pathways were executed utilizing the ClusterProfiler package (v4.10.0)^76^. To unravel the biological processes involved in microglia and microglia (*PCDH9*^high^), we employed a gene list comprising the top 500 significantly DEGs for GO term enrichment analysis using Enrichr tool^77^. The identification of microglial states and biological function was performed through gene set scoring using the gssnng toolkit (v0.4.2)^78^. The annotation information of gene sets was downloaded from Molecular Signature Database (MSigDB) (v2023.2.Hs) (https://www.gsea-msigdb.org/gsea/msigdb/).

### Predicted cell–cell communications analysis

For interacting cell prediction, cell–cell communication networks in the prefrontal cortex and hippocampus region were calculated using the CellChat R package (v1.6.1)^48^. First, the SCANPY format data were converted into the Seurat format. Next, the prefrontal cortex and hippocampus data were extracted and formatted into CellChat format. Finally, data processing and visualization of CellChat analysis were performed with default settings.

### Multiplex immunofluorescence staining

Four adult male macaque monkeys aged 6, 7 and 15 years were collected for immunostaining. Human brain tissue samples were collected from donors comprising three females aged 37, 47 and 65 years and one male aged 72 years. The brain tissues were fixed with 4% paraformaldehyde for up to 24 h and then cryoprotected in 30% sucrose at 4 °C for 72 h. The tissue samples were frozen in optimal cutting temperature compound (Tissue-Tek) at −80 °C and sectioned at 30 μm on a cryostat microtome (Leica CM1950). Sections were rinsed in phosphate-buffered saline and incubated for 30 min in 0.3% Triton X-100 (Sigma-Aldrich) and then for 2 h in 5% donkey serum (Vector Laboratories). Subsequently, sections were incubated overnight at 4 °C with the primary antibodies and for 2 h at room temperature with the secondary antibodies. The antibodies used included mouse anti-SOX-2, 1:1,000 dilution; rabbit anti-Ki67, 1:200; mouse anti-Ki67, 1:100; chicken anti-GFAP, 1:2,000; goat doublecortin (C-18), 1:1,000; rabbit anti-MASH1 (ASCL1), 1:100; rabbit PCDH9, 1:500; goat SPP1, 1:1,000; mouse anti-MAG, 1:500; and goat anti-IBA1, 1:200. Sections were mounted with 4′,6-diamidino-2-phenylindole (Abcam) and then coverslipped. Images were obtained with an LSM880 Zeiss confocal microscope.

### Web portal development

The website (Supplementary Fig. 11) was developed on the Nginx (v1.18.0) server of Ubuntu 22.04.2 LTS. The front end of the server was developed with VueJS (v2.0) (https://vuejs.org/), and the back end was built in Java using the SpringBoot web framework (v2.1.6). The ‘Data Viewer’ page and other visualization modules were built with Plotly (https://plot.ly).

### Reporting summary

Further information on research design is available in the Nature Portfolio Reporting Summary linked to this article.

## Online content

Any methods, additional references, Nature Portfolio reporting summaries, source data, extended data, supplementary information, acknowledgements, peer review information; details of author contributions and competing interests; and statements of data and code availability are available at 10.1038/s41591-024-03150-z.

## Supplementary information

Supplementary Information Supplementary Figs. 1–11 and Tables 1–8 captions.

Reporting Summary

Supplementary Tables Supplementary Table 1. Description of the metadata fields in the Brain Cell Atlas. Table 2. The table of the DEGs between NPCs and other cell types. Table 3. The number of cells of different NPC gene module scores. Table 4. The DEGs associated with microglia (*PCDH9*^high^). Table 5. Gene function enrichment pathway list for microglia and microglia (*PCDH9*^high^) clusters. Table 6. Cell–cell communications analysis results between the prefrontal cortex and hippocampus. Table 7. Metadata of all datasets in the Brain Cell Atlas. Table 8. Donor information of all datasets in the Brain Cell Atlas.

## Source data

Source Data Fig. 1 Statistical source data.

Source Data Fig. 2 Statistical source data.

Source Data Fig. 3 Statistical source data.

Source Data Fig. 4 Statistical source data.

Source Data Fig. 5 Statistical source data.

Source Data Fig. 6 Statistical source data.

Source Data Extended Data Fig. 1 Statistical source data.

Source Data Extended Data Fig. 2 Statistical source data.

Source Data Extended Data Fig. 6 Statistical source data.

## Data Availability

The web portal of Brain Cell Atlas together with all the datasets is publicly available at https://www.braincellatlas.org. Data can be downloaded at https://www.braincellatlas.org/dataSet. All the originally published data are also available from the GEO repository with the following codes: GSE100394, GSE101601, GSE101901, GSE102130, GSE102827, GSE103224, GSE103723, GSE103976, GSE104158, GSE104276, GSE104323, GSE106678, GSE107122, GSE108761, GSE109447, GSE109796, GSE110823, GSE111527, GSE113576, GSE114000, GSE115600, GSE115622, GSE115746, GSE116470, GSE117295, GSE117891, GSE118020, GSE118068, GSE118257, GSE118403, GSE118918, GSE118948, GSE118953, GSE120372, GSE121654, GSE121891, GSE122012, GSE122357, GSE123022, GSE123024, GSE123025, GSE123335, GSE124952, GSE125065, GSE126480, GSE126836, GSE128855, GSE129114, GSE129150, GSE129308, GSE129788, GSE130105, GSE130597, GSE130708, GSE131258, GSE131928, GSE132044, GSE132355, GSE132608, GSE132672, GSE132730, GSE134285, GSE134918, GSE135326, GSE135437, GSE135827, GSE136455, GSE138852, GSE138903, GSE139448, GSE140231, GSE140817, GSE140883, GSE141044, GSE141856, GSE141862, GSE142245, GSE142267, GSE142653, GSE143758, GSE143949, GSE144136, GSE144462, GSE145708, GSE146298, GSE146639, GSE147247, GSE147528, GSE148127, GSE148611, GSE148822, GSE148842, GSE149897, GSE153164, GSE154048, GSE155622, GSE157783, GSE157827, GSE157977, GSE158450, GSE160189, GSE160486, GSE160936, GSE161936, GSE162170, GSE163018, GSE163122, GSE163480, GSE164401, GSE165233, GSE165371, GSE165388, GSE167494, GSE168323, GSE168704, GSE173278, GSE173279, GSE174332, GSE174574, GSE176063, GSE178217, GSE178265, GSE178957, GSE179590, GSE180345, GSE181363, GSE182211, GSE185277, GSE185553, GSE186538, GSE187875, GSE188528, GSE190815, GSE193884, GSE198323, GSE199243, GSE200642, GSE212199, GSE231790, GSE70630, GSE71585, GSE75330, GSE76381, GSE84465, GSE87544, GSE89567, GSE93374, GSE93421, GSE95133, GSE95315, GSE95753, GSE97930, GSE98816, GSE98969, PRJNA434002, PRJNA544731 and PRJNA637987; from the ArrayExpress E-MTAB-12001, E-MTAB-8230 and E-MTAB-10974; from Single-Cell Portal SCP354, SCP815; from Neuroscience Multi-omic Archive (NeMO Archive) SCR_015820, SCR_016152; from Human Cell Atlas SRP135960; from Allen Brain Map (https://portal.brain-map.org/atlases-and-data/rnaseq#Human_Cortex); and from URLs http://bit.ly/cortexSingleCell, http://compbio.mit.edu/scADbbb/, http://development.psychencode.org/, https://cells.ucsc.edu/?ds=adult-brain-vasc, https://figshare.com/articles/dataset/EEL_Mouse_440_genes_single_cell_data/20310771?file=37550806 (ref. ^79^), https://github.com/linnarsson-lab/developing-human-brain/, https://portal.brain-map.org/atlases-and-data/rnaseq/mouse-whole-cortex-and-hippocampus-10x, https://portal.brain-map.org/explore/classes/multimodal-characterization and https://www.covid19cellatlas.org/aldinger20/, https://prod-dcd-datasets-cache-zipfiles.s3.eu-west-1.amazonaws.com/ypx3sw2f7c-1.zip. Gene sets are downloaded from MSigDB (https://www.gsea-msigdb.org/gsea/msigdb/). Source data are provided with this paper.

## Code availability

Codes for reproducing this work are available via GitHub at https://github.com/rnacentre/BrainCellAtlas-reproducibility.

## References

[1] Zheng, G. X. Y. et al. Massively parallel digital transcriptional profiling of single cells. *Nat. Commun.* **8**, 14049 (2017). 28091601 10.1038/ncomms14049 PMC5241818

[2] Ecker, J. R. et al. The BRAIN initiative cell census consortium: lessons learned toward generating a comprehensive brain cell atlas. *Neuron* **96**, 542–557 (2017). 29096072 10.1016/j.neuron.2017.10.007 PMC5689454

[3] Ding, J. et al. Systematic comparison of single-cell and single-nucleus RNA-sequencing methods. *Nat. Biotechnol.* **38**, 737–746 (2020). 32341560 10.1038/s41587-020-0465-8 PMC7289686

[4] Regev, A. et al. The Human Cell Atlas. *eLife* **6**, e27041 (2017). 29206104 10.7554/eLife.27041 PMC5762154

[5] HuBMAP Consortium. The human body at cellular resolution: the NIH Human Biomolecular Atlas Program. *Nature* **574**, 187–192 (2019). 31597973 10.1038/s41586-019-1629-x PMC6800388

[6] BRAIN Initiative Cell Census Network (BICCN). A multimodal cell census and atlas of the mammalian primary motor cortex. *Nature* **598**, 86–102 (2021). 34616075 10.1038/s41586-021-03950-0 PMC8494634

[7] Bakken, T. E. et al. Comparative cellular analysis of motor cortex in human, marmoset and mouse. *Nature* **598**, 111–119 (2021). 34616062 10.1038/s41586-021-03465-8 PMC8494640

[8] Habib, N. et al. Div-Seq: single-nucleus RNA-seq reveals dynamics of rare adult newborn neurons. *Science* **353**, 925–928 (2016). 27471252 10.1126/science.aad7038 PMC5480621

[9] Tasic, B. et al. Shared and distinct transcriptomic cell types across neocortical areas. *Nature* **563**, 72–78 (2018). 30382198 10.1038/s41586-018-0654-5 PMC6456269

[10] Siletti, K. et al. Transcriptomic diversity of cell types across the adult human brain. *Science* **382**, eadd7046 (2023). 37824663 10.1126/science.add7046

[11] Braun, E. et al. Comprehensive cell atlas of the first-trimester developing human brain. *Science* **382**, eadf1226 (2023). 37824650 10.1126/science.adf1226

[12] Tosoni, G. et al. Mapping human adult hippocampal neurogenesis with single-cell transcriptomics: reconciling controversy or fueling the debate? *Neuron* **111**, 1714–1731.e3 (2023). 37015226 10.1016/j.neuron.2023.03.010

[13] Zhong, S. et al. Decoding the development of the human hippocampus. *Nature* **577**, 531–536 (2020). 31942070 10.1038/s41586-019-1917-5

[14] Zhou, Y. et al. Molecular landscapes of human hippocampal immature neurons across lifespan. *Nature* **607**, 527–533 (2022). 35794479 10.1038/s41586-022-04912-w PMC9316413

[15] Tran, M. N. et al. Single-nucleus transcriptome analysis reveals cell-type-specific molecular signatures across reward circuitry in the human brain. *Neuron* **109**, 3088–3103.e5 (2021). 34582785 10.1016/j.neuron.2021.09.001 PMC8564763

[16] Grubman, A. et al. A single-cell atlas of entorhinal cortex from individuals with Alzheimer’s disease reveals cell-type-specific gene expression regulation. *Nat. Neurosci.* **22**, 2087–2097 (2019). 31768052 10.1038/s41593-019-0539-4

[17] Kihara, Y. et al. Single-nucleus RNA-seq of normal-appearing brain regions in relapsing-remitting vs. secondary progressive multiple sclerosis: implications for the efficacy of Fingolimod. *Front. Cell. Neurosci.* **16**, 918041 (2022). 35783097 10.3389/fncel.2022.918041 PMC9247150

[18] Franjic, D. et al. Transcriptomic taxonomy and neurogenic trajectories of adult human, macaque, and pig hippocampal and entorhinal cells. *Neuron* **110**, 452–469.e14 (2022). 34798047 10.1016/j.neuron.2021.10.036 PMC8813897

[19] Hao, Z.-Z. et al. Single-cell transcriptomics of adult macaque hippocampus reveals neural precursor cell populations. *Nat. Neurosci.* **25**, 805–817 (2022). 35637371 10.1038/s41593-022-01073-x

[20] Wang, W. et al. Transcriptome dynamics of hippocampal neurogenesis in macaques across the lifespan and aged humans. *Cell Res.* **32**, 729–743 (2022). 35750757 10.1038/s41422-022-00678-y PMC9343414

[21] Stratoulias, V., Venero, J. L., Tremblay, M.-È. & Joseph, B. Microglial subtypes: diversity within the microglial community. *EMBO J.* **38**, e101997 (2019). 31373067 10.15252/embj.2019101997 PMC6717890

[22] Boche, D. & Gordon, M. N. Diversity of transcriptomic microglial phenotypes in aging and Alzheimer’s disease. *Alzheimers Dement.* **18**, 360–376 (2022). 34223696 10.1002/alz.12389 PMC9059230

[23] Svensson, V., da Veiga Beltrame, E. & Pachter, L. A curated database reveals trends in single-cell transcriptomics. *Database* **2020**, baaa073 (2020). 33247933 10.1093/database/baaa073 PMC7698659

[24] Clough, E. & Barrett, T. The Gene Expression Omnibus database. *Methods Mol. Biol.* **1418**, 93–110 (2016). 27008011 10.1007/978-1-4939-3578-9_5 PMC4944384

[25] Speir, M. L. et al. UCSC Cell Browser: visualize your single-cell data. *Bioinformatics* **37**, 4578–4580 (2021). 34244710 10.1093/bioinformatics/btab503 PMC8652023

[26] Parkinson, H. et al. ArrayExpress—a public database of microarray experiments and gene expression profiles. *Nucleic Acids Res.* **35**, D747–D750 (2007). 17132828 10.1093/nar/gkl995 PMC1716725

[27] Xu, C. et al. Probabilistic harmonization and annotation of single-cell transcriptomics data with deep generative models. *Mol. Syst. Biol.* **17**, e9620 (2021). 33491336 10.15252/msb.20209620 PMC7829634

[28] Miao, Z. et al. Putative cell type discovery from single-cell gene expression data. *Nat. Methods* **17**, 621–628 (2020). 32424270 10.1038/s41592-020-0825-9

[29] Rousseeuw, P. J. Silhouettes: a graphical aid to the interpretation and validation of cluster analysis. *J. Comput. Appl. Math.* **20**, 53–65 (1987). 10.1016/0377-0427(87)90125-7

[30] Ayhan, F. et al. Resolving cellular and molecular diversity along the hippocampal anterior-to-posterior axis in humans. *Neuron* **109**, 2091–2105.e6 (2021). 34051145 10.1016/j.neuron.2021.05.003 PMC8273123

[31] Hochgerner, H., Zeisel, A., Lönnerberg, P. & Linnarsson, S. Conserved properties of dentate gyrus neurogenesis across postnatal development revealed by single-cell RNA sequencing. *Nat. Neurosci.* **21**, 290–299 (2018). 29335606 10.1038/s41593-017-0056-2

[32] Korsunsky, I. et al. Fast, sensitive and accurate integration of single-cell data with Harmony. *Nat. Methods* **16**, 1289–1296 (2019). 31740819 10.1038/s41592-019-0619-0 PMC6884693

[33] Haghverdi, L., Büttner, M., Wolf, F. A., Buettner, F. & Theis, F. J. Diffusion pseudotime robustly reconstructs lineage branching. *Nat. Methods* **13**, 845–848 (2016). 27571553 10.1038/nmeth.3971

[34] Bergen, V., Lange, M., Peidli, S., Wolf, F. A. & Theis, F. J. Generalizing RNA velocity to transient cell states through dynamical modeling. *Nat. Biotechnol.* **38**, 1408–1414 (2020). 32747759 10.1038/s41587-020-0591-3

[35] Lugert, S. et al. Quiescent and active hippocampal neural stem cells with distinct morphologies respond selectively to physiological and pathological stimuli and aging. *Cell Stem Cell* **6**, 445–456 (2010). 20452319 10.1016/j.stem.2010.03.017

[36] Lau, S.-F., Cao, H., Fu, A. K. Y. & Ip, N. Y. Single-nucleus transcriptome analysis reveals dysregulation of angiogenic endothelial cells and neuroprotective glia in Alzheimer’s disease. *Proc. Natl Acad. Sci. USA* **117**, 25800–25809 (2020). 32989152 10.1073/pnas.2008762117 PMC7568283

[37] Sun, N. et al. Human microglial state dynamics in Alzheimer’s disease progression. *Cell* **186**, 4386–4403.e29 (2023). 37774678 10.1016/j.cell.2023.08.037 PMC10644954

[38] Li, Q. et al. Developmental heterogeneity of microglia and brain myeloid cells revealed by deep single-cell RNA sequencing. *Neuron* **101**, 207–223.e10 (2019). 30606613 10.1016/j.neuron.2018.12.006 PMC6336504

[39] Shindou, H. et al. Relief from neuropathic pain by blocking of the platelet-activating factor-pain loop. *FASEB J.* **31**, 2973–2980 (2017). 28341636 10.1096/fj.201601183R PMC5471516

[40] Butovsky, O. et al. Identification of a unique TGF-β-dependent molecular and functional signature in microglia. *Nat. Neurosci.* **17**, 131–143 (2014). 24316888 10.1038/nn.3599 PMC4066672

[41] Unlu, G. et al. Metabolic-scale gene activation screens identify SLCO2B1 as a heme transporter that enhances cellular iron availability. *Mol. Cell* **82**, 3750 (2022). 36206741 10.1016/j.molcel.2022.09.004 PMC10334813

[42] Gosselin, D. et al. An environment-dependent transcriptional network specifies human microglia identity. *Science* **356**, eaal3222 (2017). 28546318 10.1126/science.aal3222 PMC5858585

[43] Stogsdill, J. A. et al. Pyramidal neuron subtype diversity governs microglia states in the neocortex. *Nature* **608**, 750–756 (2022). 35948630 10.1038/s41586-022-05056-7 PMC10502800

[44] Hammond, T. R. et al. Single-cell RNA sequencing of microglia throughout the mouse lifespan and in the injured brain reveals complex cell-state changes. *Immunity* **50**, 253–271.e6 (2019). 30471926 10.1016/j.immuni.2018.11.004 PMC6655561

[45] Keren-Shaul, H. et al. A unique microglia type associated with restricting development of Alzheimer’s disease. *Cell* **169**, 1276–1290.e17 (2017). 28602351 10.1016/j.cell.2017.05.018

[46] Sood, D. et al. 3D extracellular matrix microenvironment in bioengineered tissue models of primary pediatric and adult brain tumors. *Nat. Commun.* **10**, 4529 (2019). 31586101 10.1038/s41467-019-12420-1 PMC6778192

[47] Peng, Z. et al. Dlg1 knockout inhibits microglial activation and alleviates lipopolysaccharide-induced depression-like behavior in mice. *Neurosci. Bull.* **37**, 1671–1682 (2021). 34490521 10.1007/s12264-021-00765-x PMC8643377

[48] Jin, S. et al. Inference and analysis of cell–cell communication using CellChat. *Nat. Commun.* **12**, 1088 (2021). 33597522 10.1038/s41467-021-21246-9 PMC7889871

[49] Moreno-Jiménez, E. P. et al. Adult hippocampal neurogenesis is abundant in neurologically healthy subjects and drops sharply in patients with Alzheimer’s disease. *Nat. Med.* **25**, 554–560 (2019). 30911133 10.1038/s41591-019-0375-9

[50] Tobin, M. K. et al. Human hippocampal neurogenesis persists in aged adults and Alzheimer’s disease patients. *Cell Stem Cell* **24**, 974–982.e3 (2019). 31130513 10.1016/j.stem.2019.05.003 PMC6608595

[51] Sorrells, S. F. et al. Human hippocampal neurogenesis drops sharply in children to undetectable levels in adults. *Nature* **555**, 377–381 (2018). 29513649 10.1038/nature25975 PMC6179355

[52] Zhang, D. et al. Spatial epigenome-transcriptome co-profiling of mammalian tissues. *Nature* **616**, 113–122 (2023). 36922587 10.1038/s41586-023-05795-1 PMC10076218

[53] Jessberger, S., Toni, N., Clemenson, G. D. Jr, Ray, J. & Gage, F. H. Directed differentiation of hippocampal stem/progenitor cells in the adult brain. *Nat. Neurosci.* **11**, 888–893 (2008). 18587391 10.1038/nn.2148 PMC2795354

[54] Krasemann, S. et al. The TREM2–APOE pathway drives the transcriptional phenotype of dysfunctional microglia in neurodegenerative diseases. *Immunity* **47**, 566–581.e9 (2017). 28930663 10.1016/j.immuni.2017.08.008 PMC5719893

[55] Wang, S. et al. TREM2 drives microglia response to amyloid-β via SYK-dependent and -independent pathways. *Cell* **185**, 4153–4169.e19 (2022). 36306735 10.1016/j.cell.2022.09.033 PMC9625082

[56] Safaiyan, S. et al. White matter aging drives microglial diversity. *Neuron* **109**, 1100–1117.e10 (2021). 33606969 10.1016/j.neuron.2021.01.027

[57] Wlodarczyk, A. et al. A novel microglial subset plays a key role in myelinogenesis in developing brain. *EMBO J.* **36**, 3292–3308 (2017). 28963396 10.15252/embj.201696056 PMC5686552

[58] Squair, J. W. et al. Confronting false discoveries in single-cell differential expression. *Nat. Commun.* **12**, 5692 (2021). 34584091 10.1038/s41467-021-25960-2 PMC8479118

[59] Zimmerman, K. D., Espeland, M. A. & Langefeld, C. D. A practical solution to pseudoreplication bias in single-cell studies. *Nat. Commun.* **12**, 738 (2021). 33531494 10.1038/s41467-021-21038-1 PMC7854630

[60] Wolf, F. A., Angerer, P. & Theis, F. J. SCANPY: large-scale single-cell gene expression data analysis. *Genome Biol.* **19**, 15 (2018). 29409532 10.1186/s13059-017-1382-0 PMC5802054

[61] Butler, A., Hoffman, P., Smibert, P., Papalexi, E. & Satija, R. Integrating single-cell transcriptomic data across different conditions, technologies, and species. *Nat. Biotechnol.* **36**, 411–420 (2018). 29608179 10.1038/nbt.4096 PMC6700744

[62] Vasilevsky, N. A. et al. Mondo: unifying diseases for the world, by the world. Preprint at *medRxiv*10.1101/2022.04.13.22273750. (2022).

[63] Wolock, S. L., Lopez, R. & Klein, A. M. Scrublet: computational identification of cell doublets in single-cell transcriptomic data. *Cell Syst.* **8**, 281–291.e9 (2019). 30954476 10.1016/j.cels.2018.11.005 PMC6625319

[64] Ma, F. & Pellegrini, M. ACTINN: automated identification of cell types in single cell RNA sequencing. *Bioinformatics* **36**, 533–538 (2020). 31359028 10.1093/bioinformatics/btz592

[65] Lotfollahi, M. et al. Mapping single-cell data to reference atlases by transfer learning. *Nat. Biotechnol.* **40**, 121–130 (2022). 34462589 10.1038/s41587-021-01001-7 PMC8763644

[66] de Kanter, J. K., Lijnzaad, P., Candelli, T., Margaritis, T. & Holstege, F. C. P. CHETAH: a selective, hierarchical cell type identification method for single-cell RNA sequencing. *Nucleic Acids Res.* **47**, e95 (2019). 31226206 10.1093/nar/gkz543 PMC6895264

[67] Kiselev, V. Y., Yiu, A. & Hemberg, M. scmap: projection of single-cell RNA-seq data across data sets. *Nat. Methods* **15**, 359–362 (2018). 29608555 10.1038/nmeth.4644

[68] Tan, Y. & Cahan, P. SingleCellNet: a computational tool to classify single cell RNA-seq data across platforms and across species. *Cell Syst.* **9**, 207–213.e2 (2019). 31377170 10.1016/j.cels.2019.06.004 PMC6715530

[69] Aran, D. et al. Reference-based analysis of lung single-cell sequencing reveals a transitional profibrotic macrophage. *Nat. Immunol.* **20**, 163–172 (2019). 30643263 10.1038/s41590-018-0276-y PMC6340744

[70] Alquicira-Hernandez, J., Sathe, A., Ji, H. P., Nguyen, Q. & Powell, J. E. scPred: accurate supervised method for cell-type classification from single-cell RNA-seq data. *Genome Biol.* **20**, 264 (2019). 31829268 10.1186/s13059-019-1862-5 PMC6907144

[71] Lopez, R., Regier, J., Cole, M. B., Jordan, M. I. & Yosef, N. Deep generative modeling for single-cell transcriptomics. *Nat. Methods* **15**, 1053–1058 (2018). 30504886 10.1038/s41592-018-0229-2 PMC6289068

[72] La Manno, G. et al. RNA velocity of single cells. *Nature* **560**, 494–498 (2018). 30089906 10.1038/s41586-018-0414-6 PMC6130801

[73] Robinson, M. D., McCarthy, D. J. & Smyth, G. K. edgeR: a Bioconductor package for differential expression analysis of digital gene expression data. *Bioinformatics* **26**, 139–140 (2010). 19910308 10.1093/bioinformatics/btp616 PMC2796818

[74] Büttner, M., Miao, Z., Wolf, F. A., Teichmann, S. A. & Theis, F. J. A test metric for assessing single-cell RNA-seq batch correction. *Nat. Methods* **16**, 43–49 (2019). 30573817 10.1038/s41592-018-0254-1

[75] Blighe, K., Rana, S. & Lewis, M. EnhancedVolcano: publication-ready volcano plots with enhanced colouring and labeling. *Bioconductor* https://www.bioconductor.org/packages/devel/bioc/vignettes/EnhancedVolcano/inst/doc/EnhancedVolcano.html (2023).

[76] Yu, G., Wang, L.-G., Han, Y. & He, Q.-Y. clusterProfiler: an R package for comparing biological themes among gene clusters. *OMICS* **16**, 284–287 (2012). 22455463 10.1089/omi.2011.0118 PMC3339379

[77] Kuleshov, M. V. et al. Enrichr: a comprehensive gene set enrichment analysis web server 2016 update. *Nucleic Acids Res.* **44**, W90–W97 (2016). 27141961 10.1093/nar/gkw377 PMC4987924

[78] Gibbs, D. L., Strasser, M. K. & Huang, S. Single-cell gene set scoring with nearest neighbor graph smoothed data (gssnng). *Bioinform. Adv.* **3**, vbad150 (2023). 37886712 10.1093/bioadv/vbad150 PMC10599965

[79] Borm, L. EEL Mouse 440 genes single cell data. *figshare*10.6084/m9.figshare.20310771.v3 (2022).

