# Supplementary material for: A brain cell atlas integrating single-cell transcriptomes across human brain regions
Source: Nat Med. 2024 Aug 2;30(9):2679–91. doi: 10.1038/s41591-024-03150-z (PMC11405287; doi:10.1038/s41591-024-03150-z)
Supplement: Supplementary file 1 — Supplementary Figs. 1–11 and Tables 1–8 captions. [file 41591_2024_3150_MOESM1_ESM.pdf]

nature medicine

Supplementary information https://doi.org/10.1038/s41591-024-03150-z

# A brain cell atlas integrating single-cell transcriptomes across human brain regions

In the format provided by the authors and unedited

# Supplementary Information

## Supplementary Figures

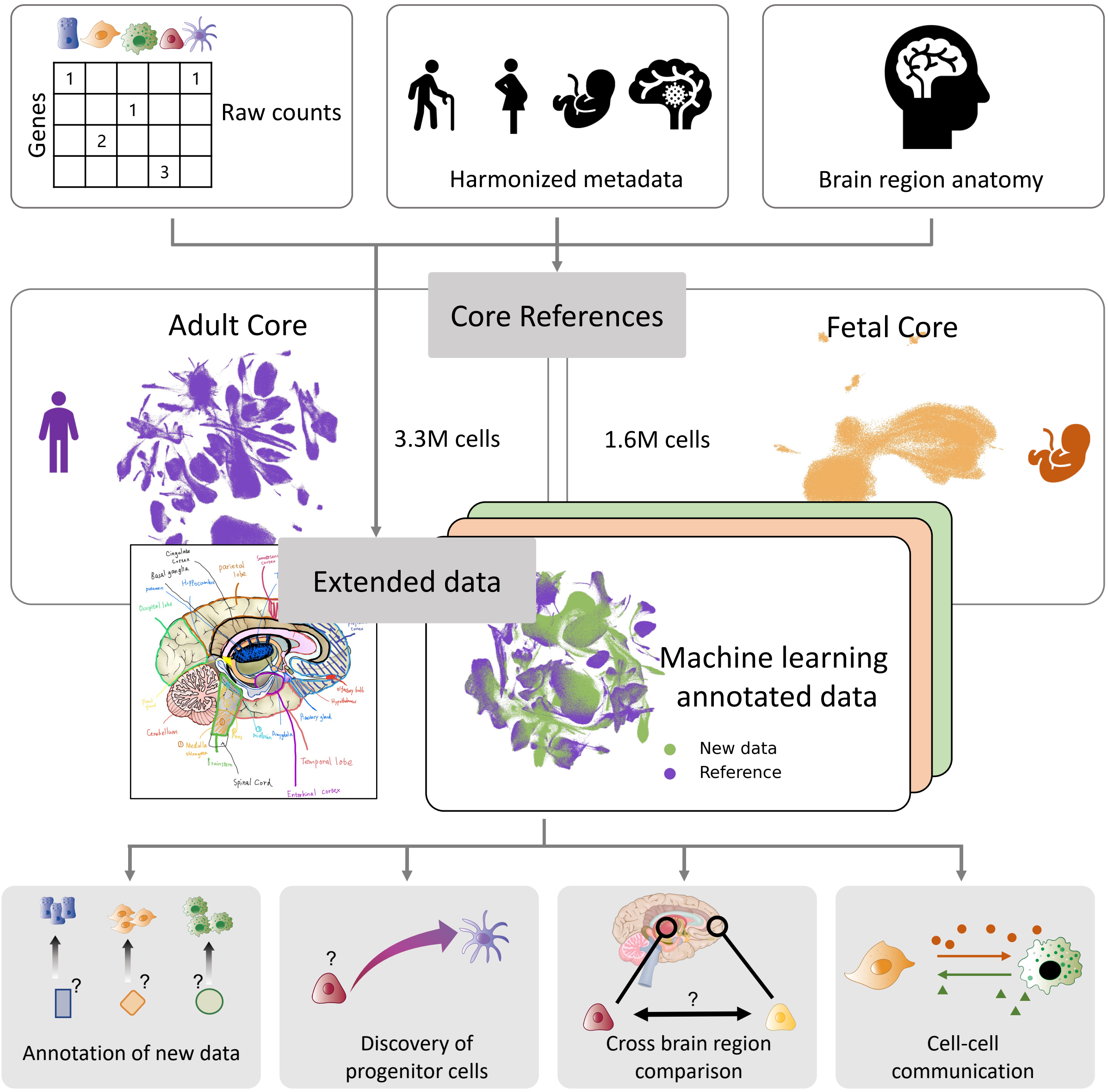


### Supplementary Fig. 1 Overview of the Brain Cell Atlas.

Raw data were collected from 70 human and 103 mouse brain studies, resulting in 11.3 million (M) cells/nuclei in humans (including fetal brains, adult brains, organoids and brain tumour) as well as 15M cells/nuclei in mice. Gene names in all the datasets were mapped to the same reference genome, while all the metadata were manually curated in a consistent manner. And the data were divided into 14 super regions in the brain, covering 30 brain subregions. Two reference cores, Siletti et al. Science 2023 for the adult brain and Braun et al. Science 2023

for the fetal brain, were prepared according to previous studies. Extended data of all the datasets were annotated using 7 reference-based machine learning methods as well as an in-house built hierarchical annotation tool (scAnnot). We showcase the applications of the data resource in annotating new data, identifying rare cell populations, performing cross-region analysis of microglia in different brain regions, and understanding the cell-cell communications.

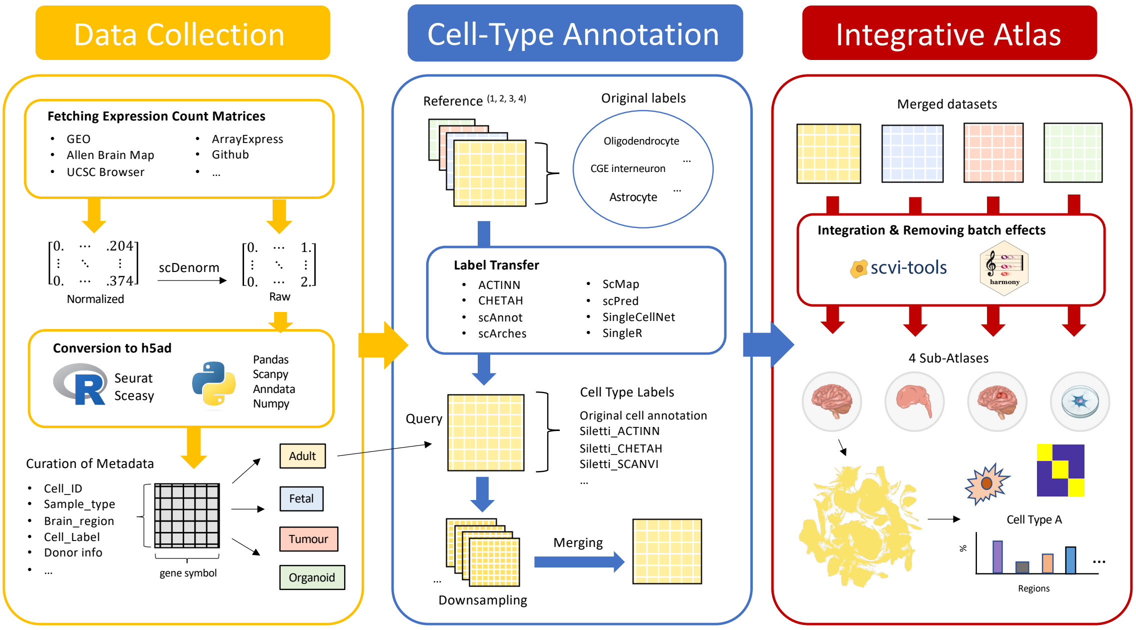


## Supplementary Fig. 2 The Scheme of Brain Cell Atlas.

The pipeline of the Brain Cell Atlas contains three stages (**Methods**). Firstly, collecting brain single-cell or single-nuclei raw count expressions matrices from public sources and sort them into one of the four sample types (Adult, Fetal, Organoid and Tumour) and manually curate for the attributes of the samples that generate the metadata, based on given information from the original studies. Secondly, annotate the cell types from the study via label transfer. Thirdly, Integrating the atlas by their sample types resulted in four sub-groups and are available for retrieval and exploration of different cell types, cross-region studies and differential analysis based on the metadata.

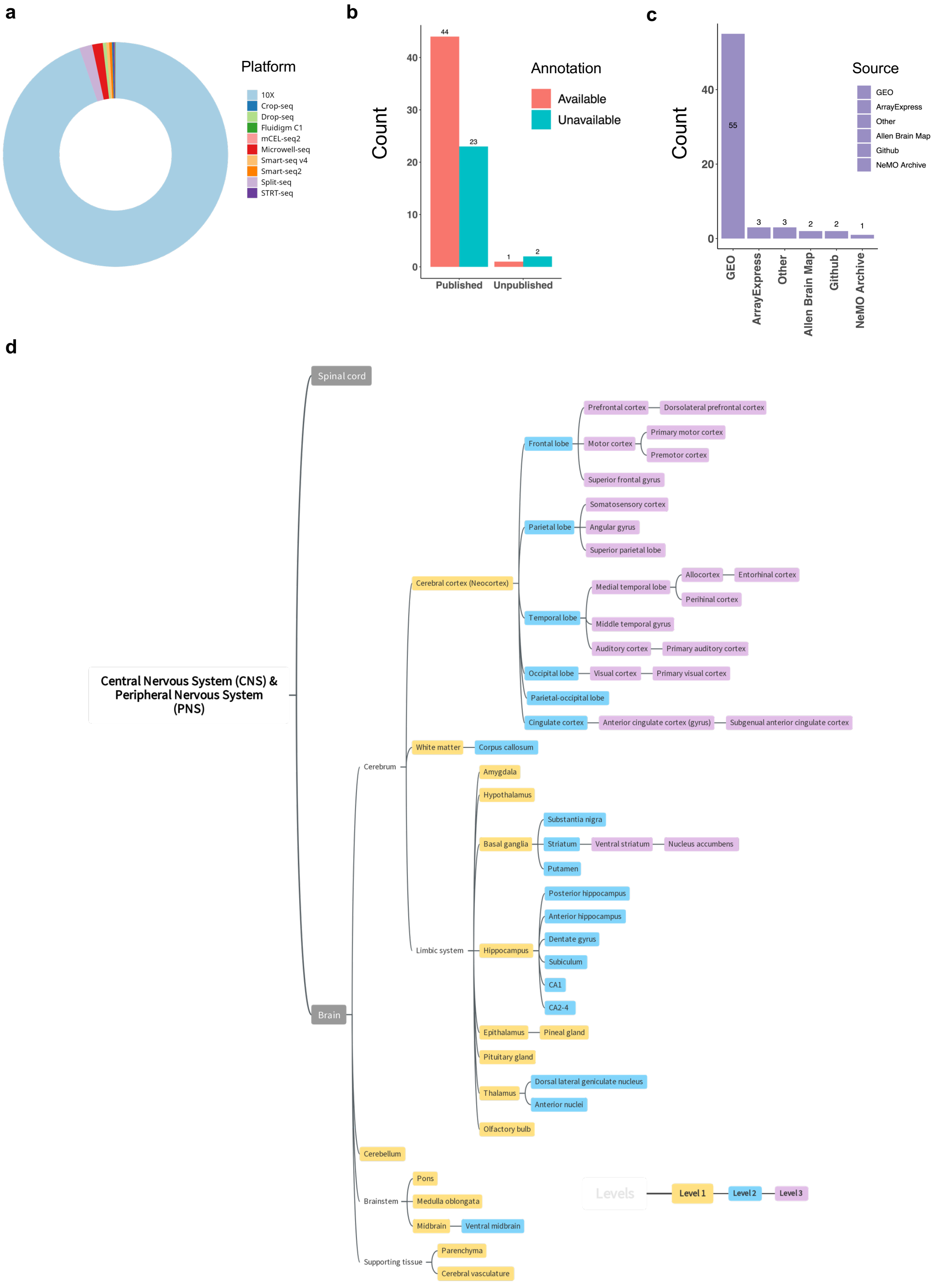


Supplementary Fig. 3 Details of the data source in Brain Cell Atlas.

**a**. Sequencing-platforms summary for all the cells (n = 11,362,024) in Brain Cell Atlas.

**b**. The total number of published data sources (n = 67) the unpublished data source (n = 3) and the cell type annotation availability in the original studies that (n = 45) or not (n = 25).
**c**. The source of the data in Brain Cell Atlas: Allen Brain Map (n = 2), ArrayExpress (n = 3), GEO (n = 55), Github (n = 2), NeMO Archive (n = 1), Other (n = 3).
**d**. Hierarchical levels of the anatomy regions where the samples are collected in Brain Cell Atlas.

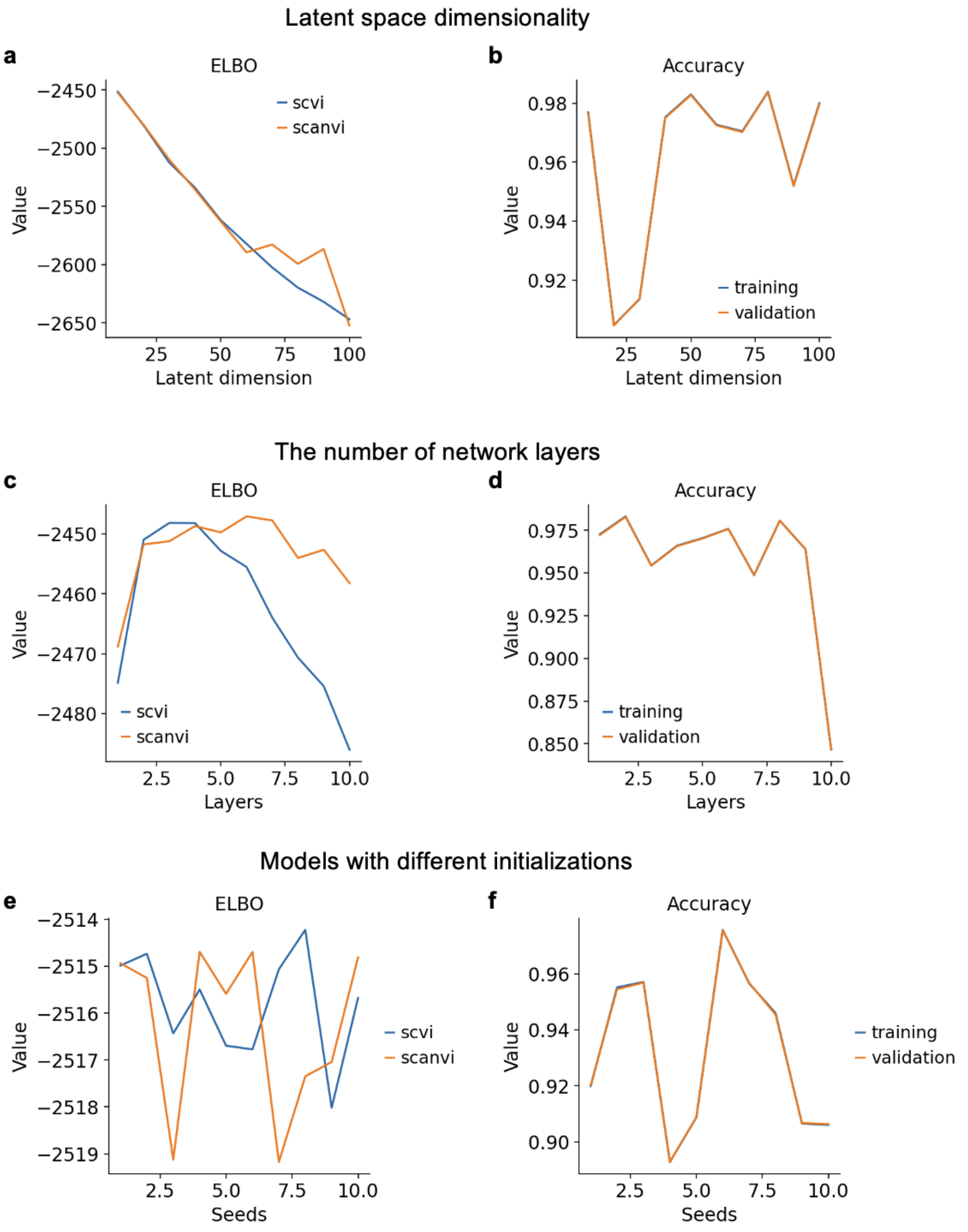


# Supplementary Fig. 4 Model selection by sweeping over different hyperparameters.

**a**. The Evidence Lower Bound (ELBO) of scANVI models with the latent space dimension from 10 to 100.

**b**. The accuracy on validation data of scANVI models training with the latent space dimension from 10 to 100.

**c**. The Evidence Lower Bound (ELBO) of scANVI models with the latent space dimension 32 and the network layers range from 1 to 10.

**d**. The accuracy on validation data of scANVI models with the latent space dimension 32 and the network layers range from 1 to 10.
**e**. The Evidence Lower Bound (ELBO) of scANVI models with the latent space dimension 32 initialising with 10 different random seeds.
**f**. The accuracy on validation data of scANVI models with the latent space dimension 32 initialising with 10 different random seeds.

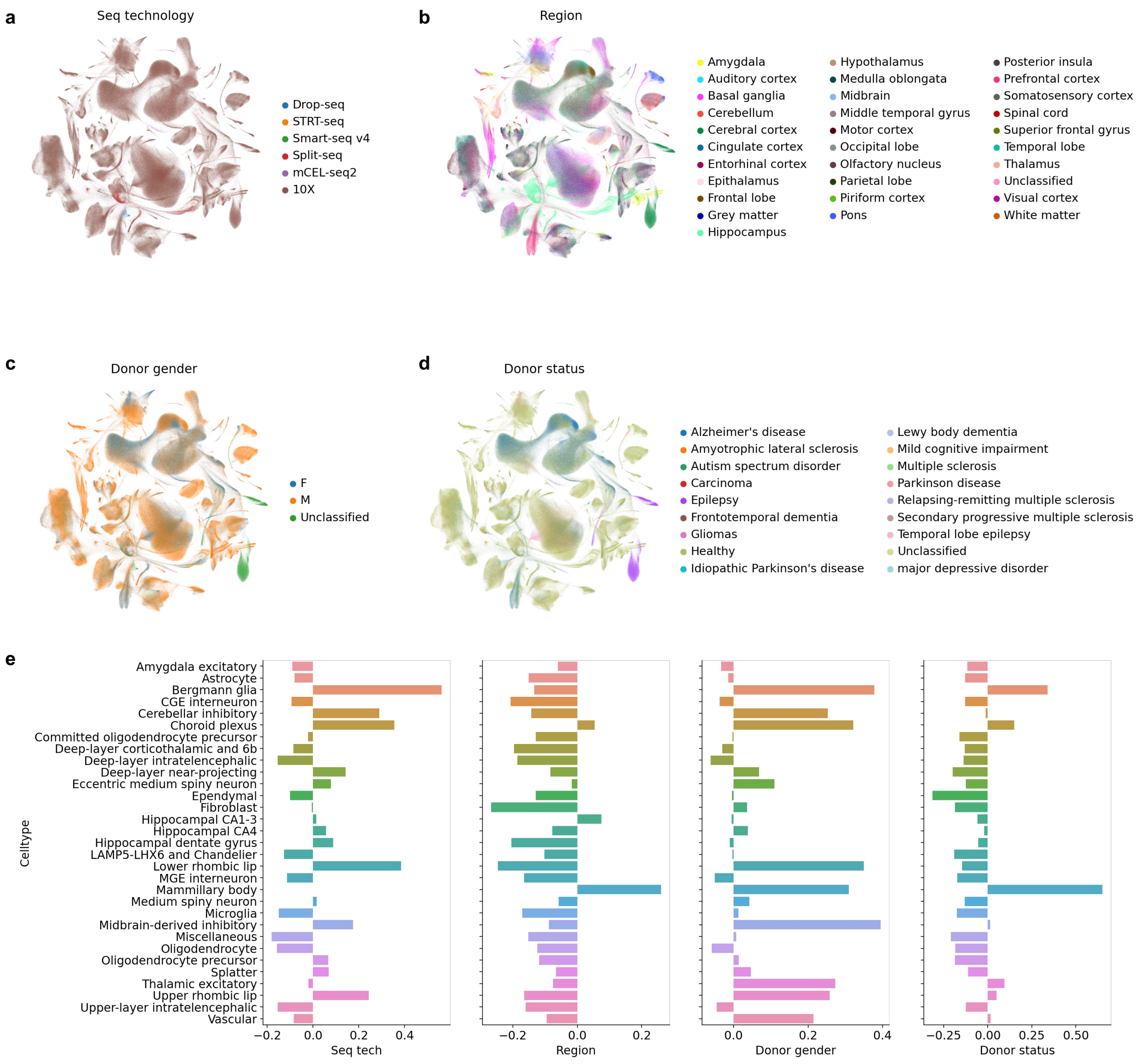


# Supplementary Fig. 5 Evaluating batch effects in Brain Cell Atlas on scANVI latent space.

**a-d**. UMAP plots of the adult brain data coloured with sequencing technology, region, donor sex and donor status, respectively.

**e**. The distribution plot of the Silhouette score measures the discrimination of sequencing technology, region, donor sex and donor status in each cell type, respectively.

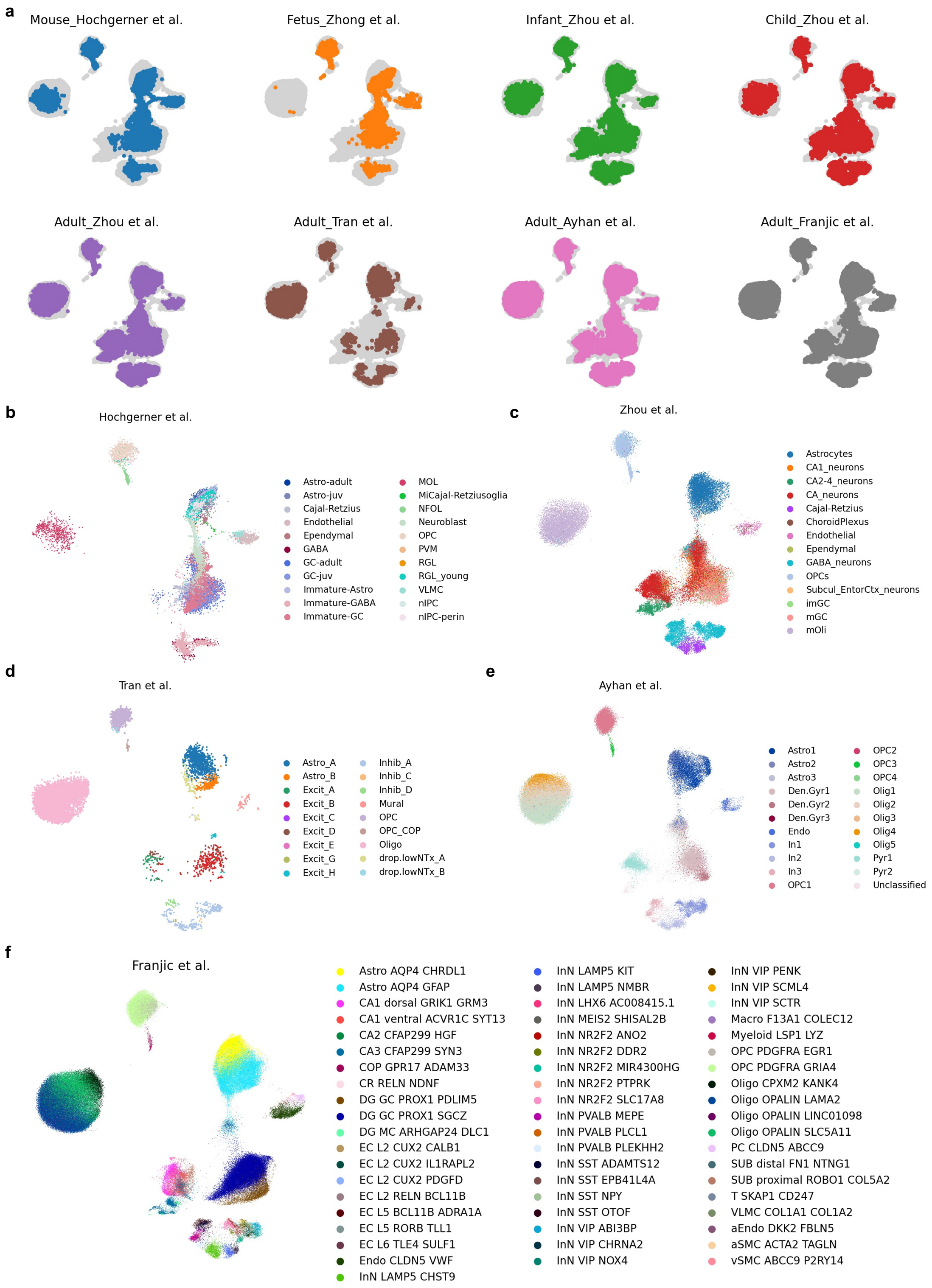


Supplementary Fig. 6 Coordinates of similar cell types were aligned across the datasets after integration.

**a**. UMAP plots separated by different datasets and age groups.

**b-f**. UMAP plots coloured with original cell type labels from studies.

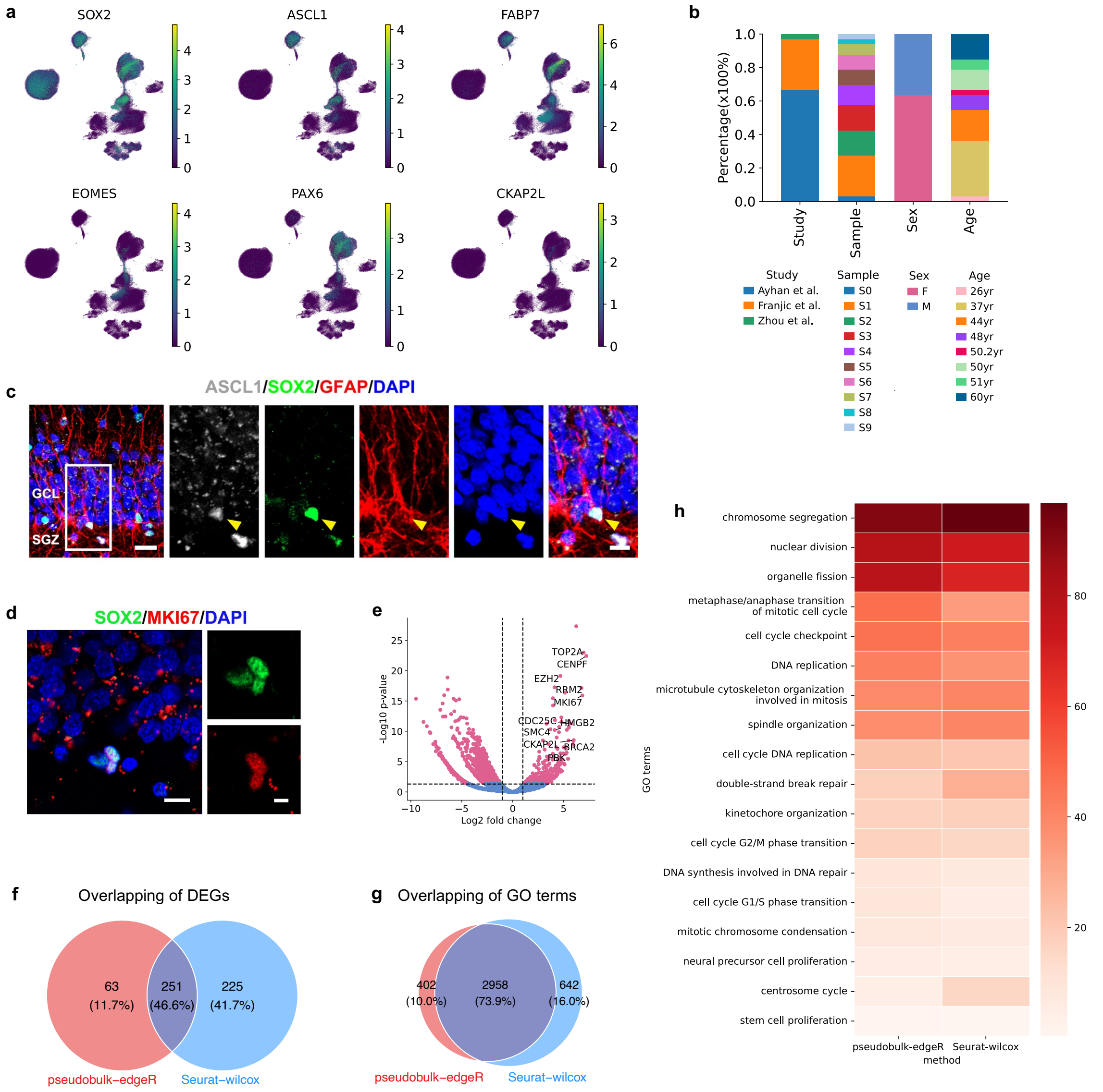


# Supplementary Fig. 7 The differential expression analysis of putative NPCs.

**a**. The UMAP plots show the expression levels of additional NPC marker genes. The intensity of the colour indicates the expression level.

**b**. Metadata with study, sample, sex, and age in putative NPCs of adult humans.

**c**. Immunolabeling for ASCL1, SOX2 and GFAP in the subgranular zone (SGZ) of the dentate gyrus (DG) of adult macaques (n = 3 specimens). Yellow arrowheads indicate ASCL1$^{+}$SOX2$^{+}$GFAP$^{+}$ NPCs. Scale bars, 20 µm (low magnification), and 10 µm (high magnification).

**d**. Immunostaining validates the existence of SOX2$^{+}$MKI67$^{+}$ NPC in the SGZ of adult macaques (n = 6 specimens). Scale bars, 10 µm (low magnification) and 5 µm (high magnification).

**e**. Volcano plot displays the edgeR likelihood ratio test for DEGs in putative NPCs compared to all other brain cell types by edgeR with pseudobulk, see **Methods**. The x-axis indicates the log2 fold change in gene expression, while the y-axis represents the negative logarithm (base 10) of the adjusted p value. Red dots represent genes that exhibit a statistically significant differential expression (adjusted p-value < 0.05 and |LogFC| > 0.5), while blue dots represent non-significant genes.

**f**. Venn diagram of the overlapping DEGs derived from Wilcoxon test and edgeR (pseudobulk).

**g**. Venn diagram of the overlapping GO terms from DEGs derived from Wilcoxon test and edgeR (pseudobulk).

**h**. Comparison among enriched GO terms of differential expressed genes derived from Wilcoxon test, and edgeR (pseudobulk).

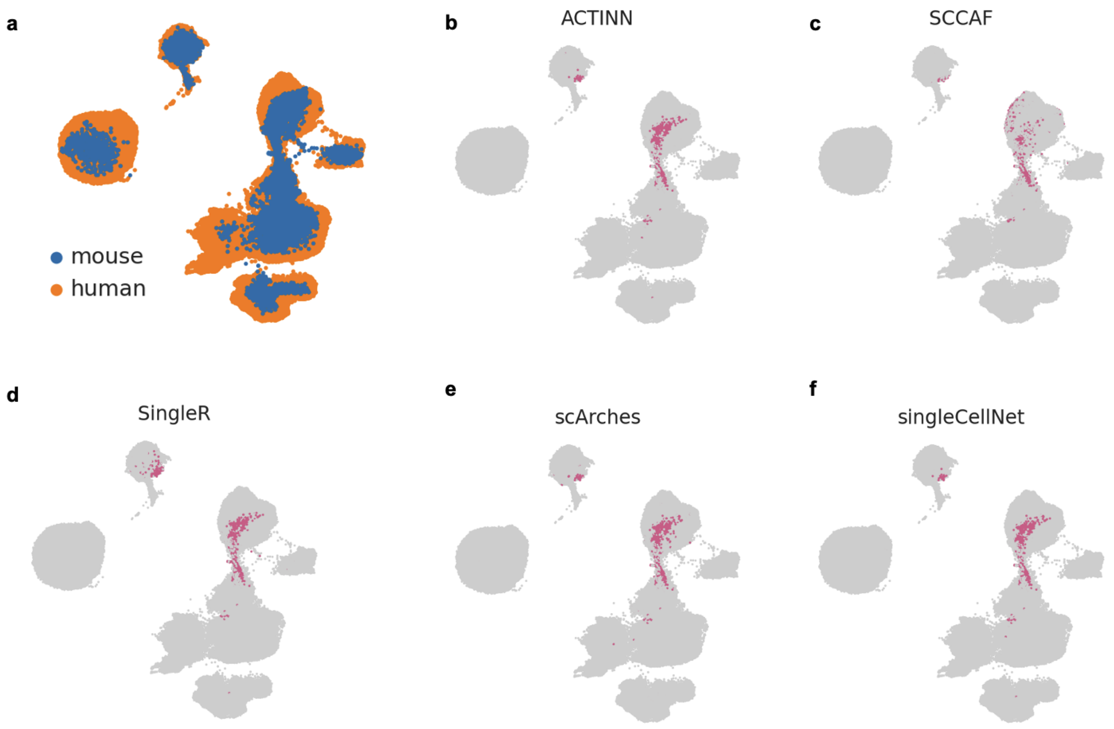


## Supplementary Fig. 8 Machine learning methods to annotate potential NPCs in adult humans.

**a**. The UMAP plot of mouse hippocampus data and human hippocampus data, coloured by species.

**b-f**. The UMAP plot of annotated potential NPCs using well-annotated developmental mouse brain data as reference by machine learning methods, ACTINN, SCCAF, SingleR, scArches, and singleCellNet.

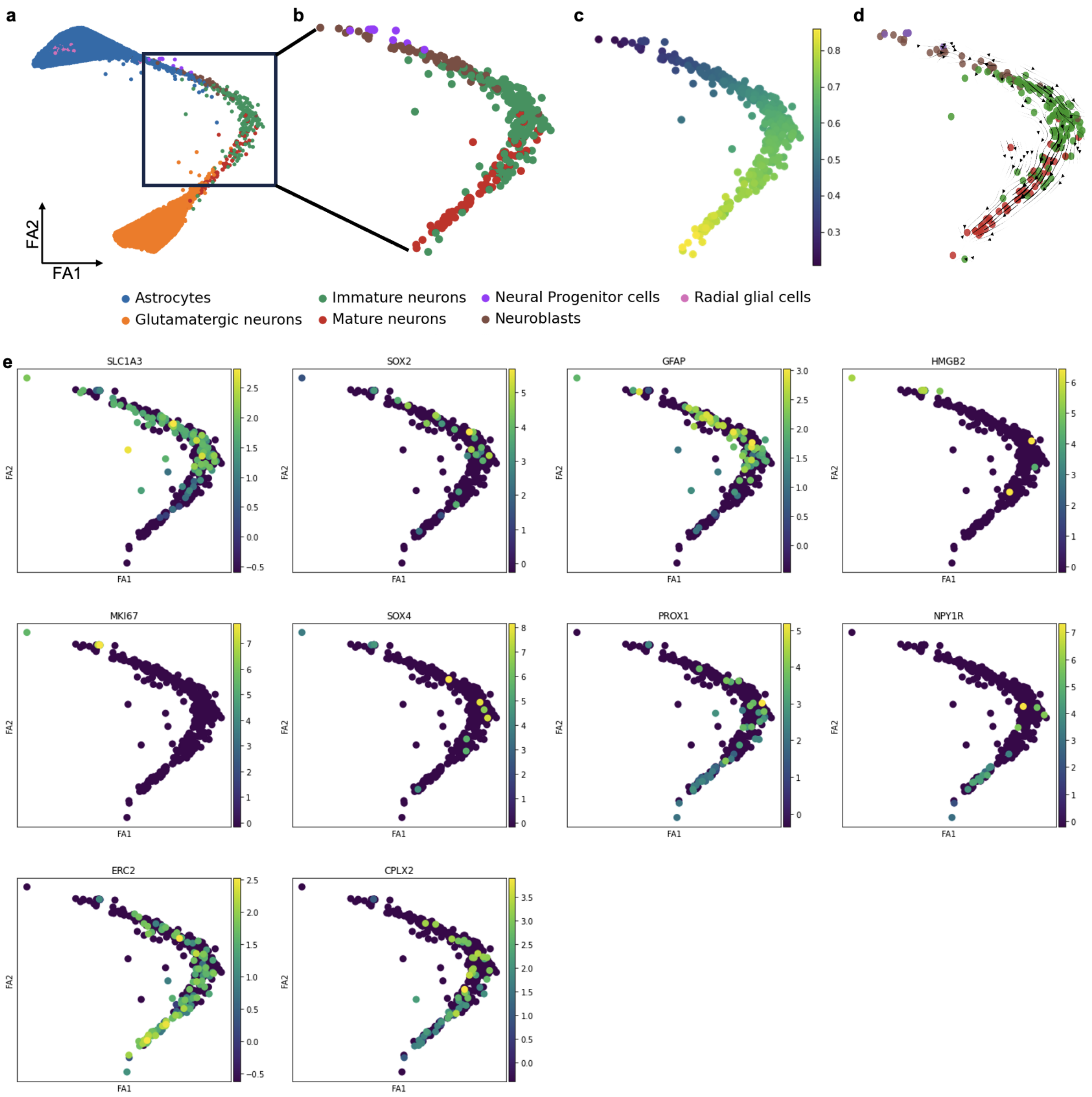


# Supplementary Fig. 9 The sub-trajectory between astrocytes and mature neurons from adult humans.

**a**. The trajectory bifurcating to astrocytes and to mature neurons on the diffusion map space, coloured by the annotated cell types.

**b**. the subplot of **a**. shows the sub-trajectory between astrocytes and mature neurons from adult humans, coloured with annotated cell types.

**c**. the pseudotime of the subplot calculated by dpt, with the start point at one of the putative NPCs. The intensity of the colour indicates the pseudotime.

**d**. RNA velocity stream plot shows the inferred developmental trajectory on the plot of the diffusion map.

**e**. The expression profiles are the distribution of normalised expression for these marker genes of astrocytes, neural progenitors, and mature neurons. The intensity of the colour indicates the expression level.

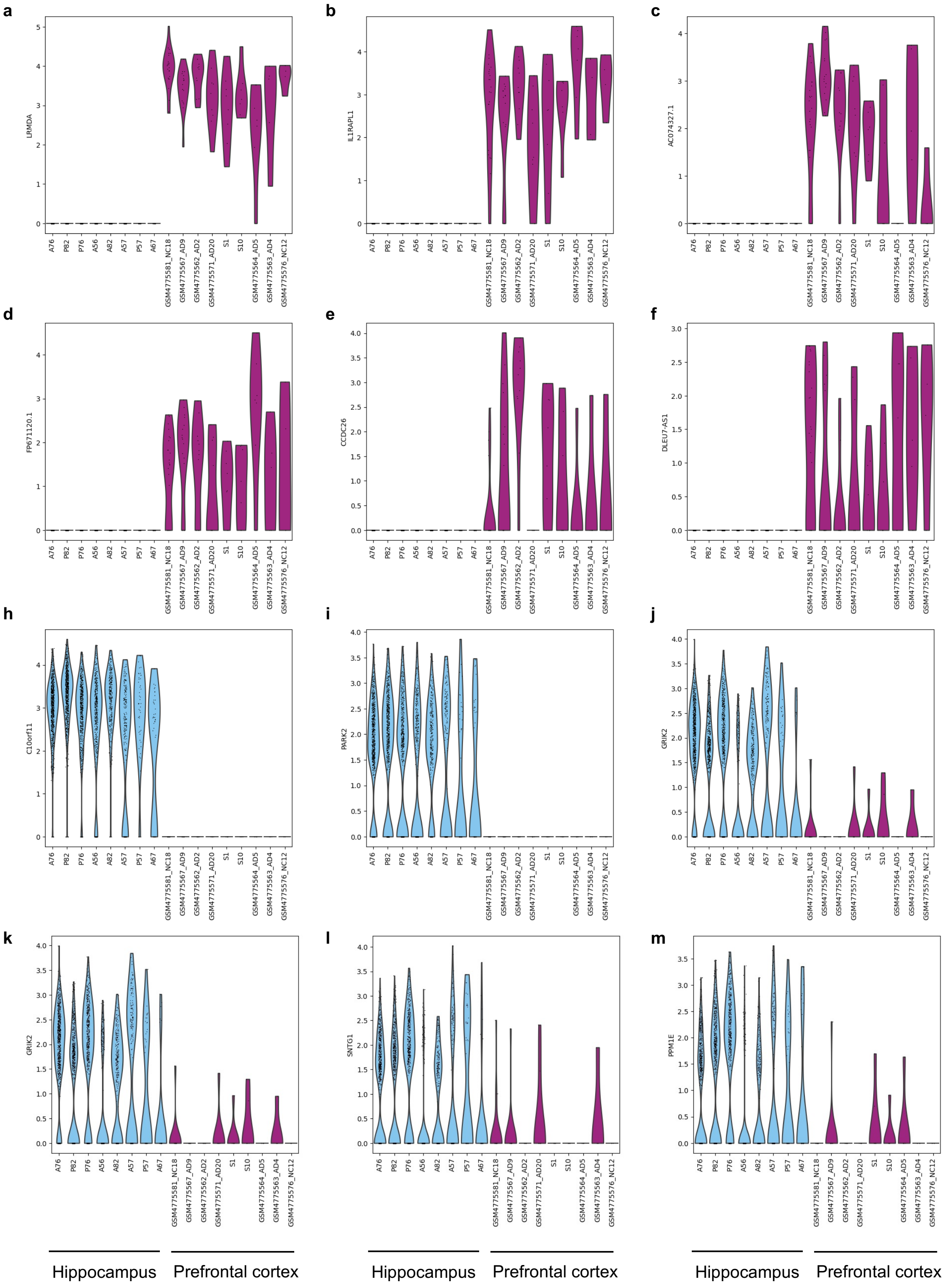


# Supplementary Fig. 10 The expression profiles of region-specific genes in microglia (*PCDH9*$^{high}$).

**a-f**. The expression profiles of genes that are highly expressed in hippocampus microglia (*PCDH9*$^{high}$).

**h-m**. The expression profiles of genes that are highly expressed in prefrontal cortex microglia (*PCDH9*$^{high}$).

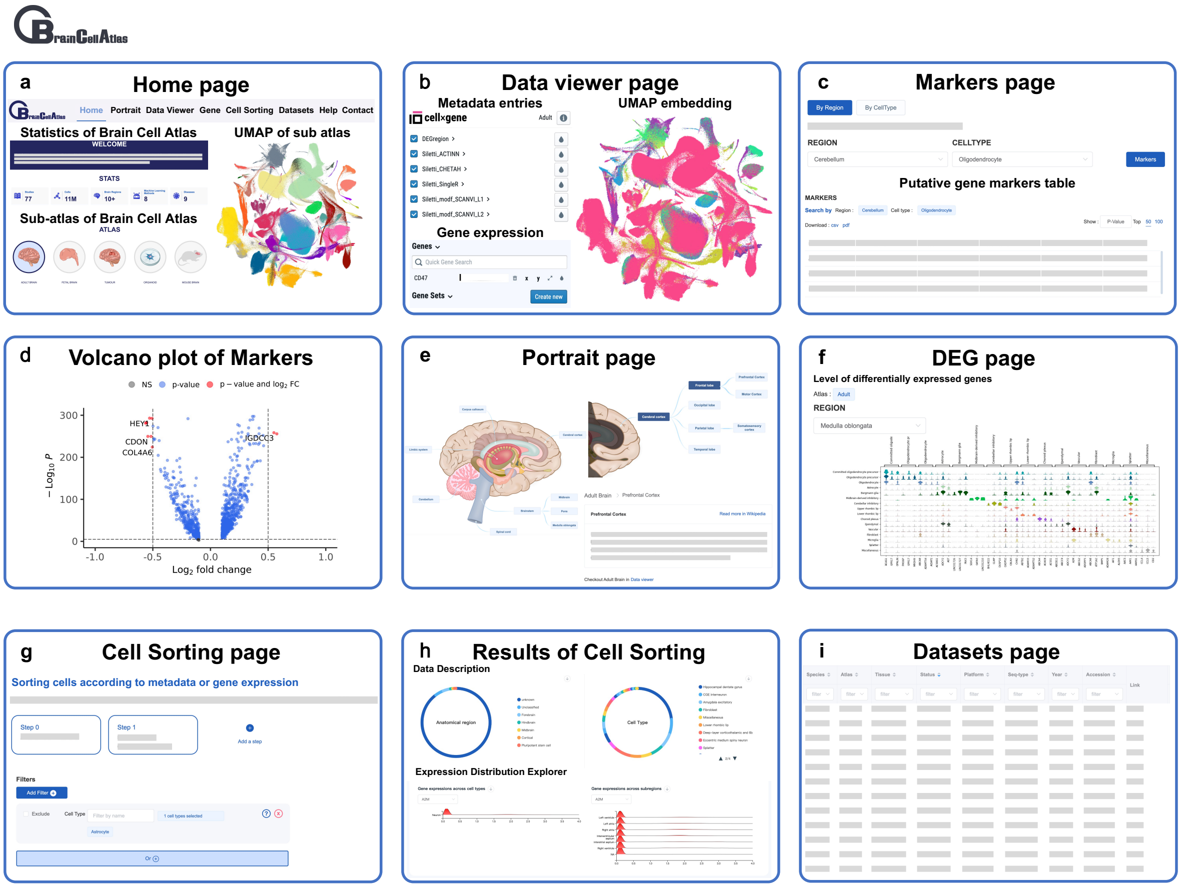


# Supplementary Fig. 11 The web portal of Brain Cell Atlas.

**a**. Homepage of the Brain Cell Atlas database. This page provides an introduction to the Brain Cell Atlas and displays a UMAP plot of the sub-atlas.

**b**. Overview of the data viewer page. This page is derived from the CELLxGENE Explorer. The left section displays categorical and numerical sample metadata, as well as features such as genes and gene sets. The right section shows the embedding, with each cell represented as a point.

**c**. Markers subpage of the gene page. It includes a module for selecting regions or cell types and an information table for putative gene markers.

**d**. A volcano plot module.

**e**. Screenshot of the Portrait page, featuring an adult human brain that encompasses all major regions found in the Brain Cell Atlas data, along with brief region descriptions sourced from Wikipedia.

**f**. Region DEG (Differentially Expressed Genes) subpage of the gene page. By selecting the sub-atlas and region of interest, a corresponding stack violin plot of gene expression is displayed, where the y-axis represents different cell types and the x-axis represents the differentially expressed genes.

**g**. Cell sorting module of the Brain Cell Atlas database, which allows the sorting of cells based on metadata or gene expression.

**h**. A screenshot showing the results of cell sorting, including a circular graph illustrating the composition of regions and cell types, as well as a gene expression distribution explorer module.

**i**. Dataset page, containing all the datasets used in the Brain Cell Atlas, along with download links for both raw and processed data.

# Supplementary Tables

## Supplementary Table 1. Description of the metadata fields in the Brain Cell Atlas.

## Supplementary Table 2. The table of the differentially expressed genes between NPCs and other cell types.

Differential expression analysis of two approaches was based on data from the adult human hippocampus. The pseudobulk-edgeR with LRT approach models batch effects along with the differential expression of biology. The Seurat-Wilcoxon test approach uses a two-sided Wilcoxon test, which does not model batch effects, and provides an easy statistical test. Benjamini-Hochberg correction was used for multiple comparisons.

## Supplementary Table 3. The number of cells of different NPC gene module scores.

## Supplementary Table 4. The differentially expressed genes associated with microglia (*PCDH9*$^{high}$).

The differential expression analysis results include two parts, (1) the DEGs between microglia (*PCDH9*$^{high}$) and other microglia cells, (2) the DEGs between microglia (*PCDH9*$^{high}$) cells in hippocampus and in prefrontal cortex. The differential expression uses the pseudobulk-edgeR with LRT approach, which models batch effects along with the differential expression of biology. Benjamini-Hochberg correction was used for multiple comparisons.

## Supplementary Table 5. Gene function enrichment pathway list for microglia and microglia (*PCDH9*$^{high}$) clusters.

This table includes gene function enrichment analysis results related to six figure panels:
(1) Fig. 4d, enriched Gene Ontology (GO) terms for the differentially expressed genes between microglia and microglia (*PCDH9*$^{high}$). The analysis was based on Enrichr, using two-sided Fisher's exact test with Benjamini-Hochberg correction for multiple comparisons.
(2) Fig. 4e, enriched KEGG pathways for the differentially expressed genes between microglia (*PCDH9*$^{high}$) and microglia. The analysis was based on clusterProfiler, using a two-sided hypergeometric test with Benjamini-Hochberg correction for multiple comparisons.
(3) Fig. 5c, enriched Gene Ontology (GO) terms for the differentially expressed genes between microglia (*PCDH9*$^{high}$) cells in prefrontal cortex (PFC) and in hippocampus (Hip). The analysis was based on clusterProfiler, using a two-sided hypergeometric test with Benjamini-Hochberg correction for multiple comparisons.
(4) Fig. 5d, enriched KEGG pathways for the differentially expressed genes between microglia (*PCDH9*$^{high}$) cells in prefrontal cortex (PFC) and in hippocampus (Hip). The analysis was based on clusterProfiler, using a two-sided hypergeometric test with Benjamini-Hochberg correction for multiple comparisons.

(5) Fig. 5f, enriched KEGG pathways identified by GSEA for the differentially expressed genes between microglia (*PCDH9*$^{high}$) cells in hippocampus (Hip) and in prefrontal cortex (PFC).
(6) Fig. 5g, enriched KEGG pathways identified by GSEA for the differentially expressed genes between microglia (*PCDH9*$^{high}$) cells in prefrontal cortex (PFC) and in hippocampus (Hip).

## Supplementary Table 6. Cell-cell communications analysis results between the prefrontal cortex and hippocampus.

The table shows standard output results from CellChat, including interaction information, cell-cell communications of ligand-receptor pairs, and information of CellChat scatter plot. P-values are computed from a one-sided permutation test from CellChat.

## Supplementary Table 7. Metadata of all datasets in the Brain Cell Atlas.

## Supplementary Table 8. Donor information of all datasets in the Brain Cell Atlas.
