# Supplementary material for: A brain cell atlas integrating single-cell transcriptomes across human brain regions
Source: Nat Med. 2024 Aug 2;30(9):2679–91. doi: 10.1038/s41591-024-03150-z (PMC11405287; doi:10.1038/s41591-024-03150-z)
Supplement: Supplementary file 2 — Reporting Summary [file 41591_2024_3150_MOESM2_ESM.pdf]

nature portfolio

Corresponding author(s): Zhichao Miao
Last updated by author(s): Jun 11, 2024

Reporting Summary

Nature Portfolio wishes to improve the reproducibility of the work that we publish. This form provides structure for consistency and transparency in reporting. For further information on Nature Portfolio policies, see our Editorial Policies and the Editorial Policy Checklist.

Statistics

For all statistical analyses, confirm that the following items are present in the figure legend, table legend, main text, or Methods section.

n/a	Confirmed	
☐	☒	The exact sample size (*n*) for each experimental group/condition, given as a discrete number and unit of measurement
☒	☐	A statement on whether measurements were taken from distinct samples or whether the same sample was measured repeatedly
☐	☒	The statistical test(s) used AND whether they are one- or two-sided<br>*Only common tests should be described solely by name; describe more complex techniques in the Methods section.*
☐	☒	A description of all covariates tested
☐	☒	A description of any assumptions or corrections, such as tests of normality and adjustment for multiple comparisons
☐	☒	A full description of the statistical parameters including central tendency (e.g. means) or other basic estimates (e.g. regression coefficient) AND variation (e.g. standard deviation) or associated estimates of uncertainty (e.g. confidence intervals)
☐	☒	For null hypothesis testing, the test statistic (e.g. *F*, *t*, *r*) with confidence intervals, effect sizes, degrees of freedom and *P* value noted<br>*Give P values as exact values whenever suitable.*
☒	☐	For Bayesian analysis, information on the choice of priors and Markov chain Monte Carlo settings
☒	☐	For hierarchical and complex designs, identification of the appropriate level for tests and full reporting of outcomes
☐	☒	Estimates of effect sizes (e.g. Cohen's *d*, Pearson's *r*), indicating how they were calculated

Our web collection on statistics for biologists contains articles on many of the points above.

Software and code

Policy information about availability of computer code

Data collection	No software was used for data collection.
Data analysis	All code is made public on GitHub, as also specified in the manuscript. GitHub - rnacentre/BrainCellAtlas-reproducibility: Notebooks to reproduce the results of Brain Cell .<br>Software versions used:<br>Scanpy (v1.9.1),<br>Anndata (v0.8.0),<br>Python (v3.10.6),<br>Seurat (v4.1.1),<br>Sceasy (v0.0.7),<br>R (v4.0.2),<br>scDenorm (v0.0.9),<br>Scrublet (v0.2.3),<br>ACTINN (v1.0.0, https://github.com/mafeiyang/ACTINN),<br>scArches (v0.5.5),<br>CHETAH (v1.9.0),<br>scmap (v1.16.0),<br>SingleCellNet (v0.4.1),<br>SingleR (v1.8.1),<br>scPred (v1.9.2),<br>scvi-tools (v0.20.3),

RNA velocity (v0.17.17),
scVelo (v0.2.5),
edgeR （v4.0.1),
Libra (v1.0.0),
EnhancedVolcano (v1.12.0),
ClusterProfiler (v3.18.1),
gssnng toolkit (v0.4.2)
MSigDB (v2023.2.Hs)
CellChat (v1.6.1)
Nginx (v1.18.0)
VueJS (v2.0)
SpringBoot web framework (v2.1.6)
CELLxGENE Explorer (v1.1.1)

For manuscripts utilizing custom algorithms or software that are central to the research but not yet described in published literature, software must be made available to editors and reviewers. We strongly encourage code deposition in a community repository (e.g. GitHub). See the Nature Portfolio guidelines for submitting code & software for further information.

Data

Policy information about availability of data

All manuscripts must include a data availability statement. This statement should provide the following information, where applicable:

- Accession codes, unique identifiers, or web links for publicly available datasets
- A description of any restrictions on data availability
- For clinical datasets or third party data, please ensure that the statement adheres to our policy

There are no restrictions on data availability. The Brain Cell Atlas is fully public.
Data Availability Statement
The web portal of Brain Cell Atlas together with all the datasets are publicly available at https://www.braincellatlas.org. Data can be downloaded at https://www.braincellatlas.org/dataSet. All the originally published data are also available from the GEO repository with codes: GSE100394, GSE101601, GSE101901, GSE102130, GSE102827, GSE103224, GSE103723, GSE103976, GSE104158, GSE104276, GSE104323, GSE106678, GSE107122, GSE108761, GSE109447, GSE109796, GSE110823, GSE111527, GSE113576, GSE114000, GSE115600, GSE115622, GSE115746, GSE116470, GSE117295, GSE117891, GSE118020, GSE118068, GSE118257, GSE118403, GSE118918, GSE118948, GSE118953, GSE120372, GSE121654, GSE121891, GSE122012, GSE122357, GSE123022, GSE123024, GSE123025, GSE123335, GSE124952, GSE125065, GSE126480, GSE126836, GSE128855, GSE129114, GSE129150, GSE129308, GSE129788, GSE130105, GSE130597, GSE130708, GSE131258, GSE131928, GSE132044, GSE132355, GSE132608, GSE132672, GSE132730, GSE134285, GSE134918, GSE135326, GSE135437, GSE135827, GSE136455, GSE138852, GSE138903, GSE139448, GSE140231, GSE140817, GSE140883, GSE141044, GSE141856, GSE141862, GSE142245, GSE142267, GSE142653, GSE143758, GSE143949, GSE144136, GSE144462, GSE145708, GSE146298, GSE146639, GSE147247, GSE147528, GSE148127, GSE148611, GSE148822, GSE148842, GSE149897, GSE153164, GSE154048, GSE155622, GSE157783, GSE157827, GSE157977, GSE158450, GSE160189, GSE160486, GSE160936, GSE161936, GSE162170, GSE163018, GSE163122, GSE163480, GSE164401, GSE165233, GSE165371, GSE165388, GSE167494, GSE168323, GSE168704, GSE173278, GSE173279, GSE174332, GSE174574, GSE176063, GSE178217, GSE178265, GSE178957, GSE179590, GSE180345, GSE181363, GSE182211, GSE185277, GSE185553, GSE186538, GSE187875, GSE188528, GSE190815, GSE193884, GSE198323, GSE199243, GSE200642, GSE212199, GSE231790, GSE70630, GSE71585, GSE75330, GSE76381, GSE84465, GSE87544, GSE89567, GSE93374, GSE93421, GSE95133, GSE95315, GSE95753, GSE97930, GSE98816, GSE98969, PRJNA434002, PRJNA544731, PRJNA637987; from the EBI E-MTAB-12001, E-MTAB-8230, E-MTAB-10974; Single-Cell Portal SCP354, SCP815, SCR_015820, SCR_016152, SRP135960, Allen Brain Map (https://portal.brain-map.org/atlases-and-data/rnaseq#Human_Cortex), and from URLs http://bit.ly/cortexSingleCell, http://compbio.mit.edu/scADbbb/, http://development.psychencode.org/#, https://cells.ucsc.edu/?ds=adult-brain-vasc, https://figshare.com/articles/dataset/EEL_Mouse_440_genes_single_cell_data/20310771?file=37550806, https://github.com/linnarsson-lab/developing-human-brain/, https://portal.brain-map.org/atlases-and-data/rnaseq/mouse-whole-cortex-and-hippocampus-10x, https://portal.brain-map.org/explore/classes/multimodal-characterization, https://www.covid19cellatlas.org/aldinger20/, https://prod-dcd-datasets-cache-zipfiles.s3.eu-west-1.amazonaws.com/ypx3sw2f7c-1.zi. Gene sets are downloaded from MSigDB (https://www.gsea-msigdb.org/gsea/msigdb/).

Research involving human participants, their data, or biological material

Policy information about studies with human participants or human data. See also policy information about sex, gender (identity/presentation), and sexual orientation and race, ethnicity and racism.

Reporting on sex and gender	The gender/sex was identified either by self-report or by medics determination and is listed on the basis of each study in Supplementary Table 7.<br>The gender information of the consequent analyses:<br>（1） For adult human neuronal progenitor cell explorations: the human samples involved 12 females and 20 males, whose sexes were either assigned by the medics or by self-report (Supplementary Table 7).<br>(2) The PCDH9+ microglia analysis involved samples of 18 females and 25 males (Supplementary Table 7).<br>For immunostaining, we used 3 female and 1 male donor.
Reporting on race, ethnicity, or other socially relevant groupings	These datasets include samples from diverse ethnicities.
Population characteristics	The gender, age and ethnicity of the donors are listed in Supplementary Table 8. The proportion of gender and age group of all the single-cells/single nuclei in the Brain Cell Atlas is also described in the main text and Fig1b-c: "The samples were obtained from donors aged from 6 Gestational Weeks (GW) to over 80 years old (Fig. 2b). In fetal data, ~65% of the samples came from embryonic brain tissue in the first trimesters (0 – 12 GW), while samples from donors from age 40 to 80 years constitute ~78% of the postnatal samples. ~9% of the samples were curated without known age. In fetal data, ~65% of the samples came from embryonic brain tissue in the first trimesters (0 – 12 GW), while samples from donors from age 40 to 80

years constitute ~78% of the postnatal samples. ~9% of the samples were curated without known age. In adult data, only ~25% of the samples were from female donors, while ~71% were from male donors, and < 5% were unknown in gender. Whereas, up to 92% of the samples had undetermined gender in fetal, and the female-to-male ratio is 1.3:1 in the rest (Fig. 1c)"
Across all the donors, around 92% have unknown ancestry, around 6% have European ancestry, 1.3% are East Asian and 0.4% are African American.
The gender information of the consequent analyses:
（1） For adult human neuronal progenitor cell explorations: the human samples involved 12 females and 20 males, whose sexes were either assigned by the medics or by self-report (Supplementary Table 7).
（2) The PCDH9+ microglia analysis involved samples of 18 females and 25 males (Supplementary Table 7).
For immunostaining, we used 3 female (37, 47, 65 years) and 1 male (72 years) donor.

Recruitment

Recruitment was done in individual studies as published.

Ethics oversight

All public datasets analysed in this manuscript have published ethics approvals available. The ethics approval information per study has been collected and summarised in Supplementary Table 7. The de-identified human tissue collection and protocols for the immunostaining assays were approved by the Ethics Committee of the Sun Yat-sen University Cancer Center. As for macaques, ethical compliance was ensured and all experimental procedures were approved by the Animal Care and Use Committee of Zhongshan Ophthalmic Center at Sun Yat-sen University. The study was performed in accordance with the Principles for the Ethical Treatment of Non-Human Primates. Adult macaques were obtained from Blooming-Spring Biotechnology Co., Ltd., in Guangdong, China, or were generously gifted from nearby laboratories at Sun Yat-sen University for terminal experiments.

human_brain_Microglia_Schirmer_2019_10x : All tissue included in this study was provided by the UK Multiple Sclerosis Tissue Bank at Imperial College, London and the University of Maryland Brain Bank through the NIH NeuroBioBank. Human MS and control tissues were obtained via a prospective donor scheme following ethical approval by the National Research Ethics Committee in the UK (08/MRE09/31). We have complied with all relevant ethical regulations regarding the use of human postmortem tissue samples. We examined a total of 35 (19 MS and 16 controls) snap-frozen brain tissue blocks obtained at autopsies from 17 MS patients and 16 controls.

human_brain_motor_cortex_Pineda_2021_10x : Human tissue analysis was conducted as exempt human research, considering frozen post-mortem brain samples obtained from the Neuropathology Laboratory at the Mayo Clinic (Jacksonville, FL USA) were not specifically collected for this study.

human_brain_source_Velmeshev_2019 : The tissues were obtained from the NIH NeuroBioBank, the University of Maryland School of Medicine Brain and Tissue Bank, which stated that they "adhere to the highest ethical standards when acquiring specimens for their collections, and protection of donor identity is assured. All brain tissue is procured, stored, and distributed according to applicable state and federal guidelines and regulations involving consent, protection of human subjects and donor anonymity.

human_brain_WhiteMatter_Jakel_2019_10x : Post-mortem unfixed fresh-frozen tissue and formalin-fixed paraffin-embedded (FFPE) tissue were obtained from the UK Multiple Sclerosis Tissue Bank via a UK prospective donor scheme with full pre-mortem consent and with full ethical approval by MREC/02/2/39 (UK Ethics Committee) and 2016/589-31 (Regionala Etiskprövningsnämnden, Stockholm).

human_brain_Microglia_Tsartsalis_2021_10x : Local tissue access and data generation was carried out in accordance with the Regional Ethics Committee and Imperial College Use of Human Tissue guidelines. Tissues and processing were described previously63. Cases were selected from the London Neurodegeneration (King's College London) and Parkinson's UK (Imperial College London) Brain Banks.

human_brain_FC_Agarwal_2020_10x : Informed consent had been collected from all cases fulfilling the requirements of the Human Tissue Act 2004.

human_brain_Ganglionic_eminences_Shi_2021_10x : Human fetal ganglionic eminence (GE) samples across gestational weeks (GW) 9 to 18 were collected from Beijing Anzhen Hospital with approval from the Reproductive Study Ethics Committee of Beijing Anzhen Hospital and the institutional review board (ethics committee) of the Institute of Biophysics.

human_brain_GBMs_LeBlanc_2021_10x : The use of tumor tissues and peripheral blood samples was coordinated through the Clark H. Smith Tumour and Related Tissue Bank. Tissues and blood samples were collected at the Foothills Medical Centre in Calgary, AB, Canada after informed patient consent under a protocol approved by the Health Research Ethics Board of Alberta Cancer Committee (HREBA.CC-16-0762). Additional work was done under HREBA.CC-16-0716 and a protocol approved by the UBC BC Cancer Research Ethics Board (H19-03010).

human_brain_glioma_Yu_2020_STRTseq : The samples and sequencing data were generated by Peking University Xiao-dong Su Lab. The work was approved by the Peking University Biomedical Ethics Committee.

human_mouse_brain_Wheeler_2020_DropSeq_10x : Use of the tissues was approved by the Montreal Neurological Institute and Hospital (MNI/H) Neurosciences Research Ethics Board under REB approval ANTJ 1988/3.

human_brain_CV_SunN_2022_10x : Human brain tissues in this study were obtained from the Religious Orders Study and Rush Memory and Aging Project (ROSMAP, each approved by an Institutional Review Board (IRB) of Rush University Medical Center) with informed consent, an Anatomic Gift Act for organ donation, and a repository consent to allow the data to be shared114. Quantitative clinical and pathologic phenotypes of AD were used to assess disease severity. These included global cognition proximate to death, and a measure of global AD pathology as well as the molecularly specific beta-amyloid and PHFtau tangles. Controls were defined as individuals with little to no AD pathology, whereas cases included a spectrum of AD

pathology. Thus, case status was based solely based on AD pathology and other variables were allowed to freely associate, as previously reported115–119.

human_brain_wholebrain_Siletti_2022_10x : De-identified postmortem adult human brain tissue was obtained after receiving permission from the deceased's next-of-kin. Tissue collection was performed in accordance with the provisions of the United States Uniform Anatomical Gift Act of 2006 described in the California Health and Safety Code section 7150 (effective 1/1/2008) and other applicable state and federal laws and regulations. The Western Institutional Review Board reviewed tissue collection procedures and determined that they did not constitute human subjects research requiring institutional review board (IRB) review. The collection and processing of human tissue was approved under Swedish law by the Swedish Ethical Review Authority (2019-03054

human_brain_source_Tran_2021 : Post-mortem human brain tissue from eight neurotypical donors of European ancestry from age 40 to 69 (Table S1) was obtained by autopsy from the Office of the Chief Medical Examiner for the State of Maryland under State of Maryland Department of Health and Mental Hygiene Protocol 12-24

human_brain_MultipleCorticalAreas_AllenBrain_2019_SMART-Seq : Tissue was provided to researchers under the supervision and authority of the Internal Review Board (IRB) of each participating hospital. All patients included in the population met with a hospital-appointed surgical case coordinator to review the option of tissue donation and voluntarily signed an IRB-approved Informed Consent Form. Patients who did not wish to participate in tissue donation or who were otherwise unable to provide written consent were excluded from the study.

human_brain_motor cortex_AllenBrain_2020_10x : Tissue was provided to researchers under the supervision and authority of the Internal Review Board (IRB) of each participating hospital. All patients included in the population met with a hospital-appointed surgical case coordinator to review the option of tissue donation and voluntarily signed an IRB-approved Informed Consent Form. Patients who did not wish to participate in tissue donation or who were otherwise unable to provide written consent were excluded from the study.

human_brain_PFC_Kihara_2022_10x : Handling of the human samples were followed with the guidelines of the Cleveland Clinic Human Research Ethics Committee. The animal study was reviewd and approved by the Institutional Animal Care and Use Committee of the Sanford Burnham Prebys Medical Discovery Institute.

human_brain_CorticalOrganoids_Ziffra_2021_ATAC-seq_10x : De-identified tissue samples were collected with previous patient consent in strict observance of the legal and institutional ethical regulations. Protocols were approved by the Human Gamete, Embryo, and Stem Cell Research Committee (institutional review board) at the University of California, San Francisco.

human_brain_FetalCerebellum_Aldinger_2021_SPLiT-seq : Acquisition of human tissue samples was approved by the Seattle Children's Hospital (SCH) Institutional Review Board. Experiments were performed in accordance with SCH ethical and legal guidelines. Specimens from fetal (9–21 PCW) human cerebellum were obtained from the Birth Defects Research Laboratory at the University of Washington or the Joint MRC/Wellcome (MR/R006237/1) Human Developmental Biology Resource51 (https://www.hdbr.org/) with ethics board approval and maternal written consent obtained before specimen collection.

human_brain_fetal_Yu_2021_10x : This study was approved by the Ethical Committee of the Quanzhou First Hospital (20162016). Human fetal brain tissues were collected from elective pregnancy termination specimens at the Quanzhou First Hospital, Fujian Province, in China. Discarded human tissues were examined only from patients who had given informed consent with no compensation. All the protocols complied with the 'Interim Measures for the Administration of Human Genetic Resources'.

human_brain_Lau_2020_10x : The SWDBB that provided the samples is part of the Brains for Dementia Research program, which is jointly funded by Alzheimer's Research UK and the Alzheimer's Society. The relevant ethics statements are as followed: "Brains for Dementia Research makes sure that the donated tissue in its care is used ethically in good quality research. Researchers submit a detailed application that is reviewed by an ethics committee so that the brain tissue, information about the donor and the wishes of their family are all respected."

human_brain_microglia_midbrain_Smajic_2022_10x : Patients and control subjects gave written informed consent with the brain banks, which, together with the ethics review panel of the University of Luxembourg, approved the study.

human_brain_Otero-Garcia_2022_10x : Human tissue was obtained from UCLA-Easton Center, NIH Neurobiobank (Sepulveda repository, Los Angeles, CA, and Mount Sinai, New York, NY), and the Stanford Alzheimer Disease Research Center (NIH/NIA P30 AG066515). Tissue samples stored in the NIH neurobiobank are "procured, stored, and distributed according to applicable state and federal guidelines and regulations involving consent, protection of human subjects and donor anonymity."

human_brain_Microglia_Kracht_2020_Smartseq2 : The collection and use of fetal material have been approved by the Medical Ethical Committee of the Leiden University Medical Centre (P08.087) and the Groningen University Medical Centre (2017/040). Tissue from human fetuses was obtained from elective abortion procedures (without medical indication) and was donated for research purposes with informed consent. T

human_brain_organoids_Fiorenzano_2021_10x : All procedures were in accordance with the European Union directive and approved by the local ethical committee at Lund University. Human fetal tissue from legally terminated embryos was collected in accordance with existing guidelines with approval of the Swedish National Board of Health and Welfare and informed consent from women seeking elective abortions.

human_brain_organoids_Revah_2022_10x : All experiments involving human cells complied with all relevant guidelines and regulations. Human donors in this study consented to the use of their cells to generate hiPS cells and derived cells. The source of the cells and their institutional approvals are listed in Supplementary Table 1. This study also benefitted from a

consultation with the Stanford Center for Law and the Biosciences on the ethical aspects of the work as part of the Stanford Big Idea Project on Brain Organogenesis (Stanford Wu Tsai Neurosciences Institute). Approval for transplantation of hCO into rats was obtained from the Stanford Laboratory Animal Care (APLAC) Research Compliance Office. No discernible locomotor or memory deficits were detected in transplanted animals and their wellbeing was monitored throughout. Surgical neural tissue samples were obtained with approval from the Stanford University Institutional Review Board.

human_brain_source_Nagy_2020 : This study was approved by the Douglas Hospital Research Ethics Board, and written informed consent from next of kin was obtained for each individual. Postmortem brain samples were provided by the Douglas–Bell Canada Brain Bank (www.douglasbrainbank.ca).

human_brain_source_Fatma_2020 : All hippocampal tissues in this study were obtained from patients with temporal lobe epilepsy with the informed consent of patients and approval of the UTSW Institutional Review Board (IRB)

human_brain_radialglia_WangR_2020_10x : Surgical specimens were collected from the surgical suite at Memorial Sloan Kettering Cancer Center, following diagnostic confirmation by a neuropathologist. Tissues were obtained in accordance with international review board guidelines

human_brain_Sadick_2022_10x : All tissues were donated with pre-mortem informed consent as regulated by Institute Review Boards at each respective Institution.

human_brain_FC_Cerebellum_Lake_2017_snDrop-Seq : All human tissue protocols were approved by the Office for Human Research Protection at Sanford Burnham Prebys Medical Discovery Institute and conformed to National Institutes of Health guidelines.

human_brain_GBM_Neftel_2019_Smartseq2_10x : Adult patients at Massachusetts General Hospital (MGH) and pediatric patients and their parents at Boston Children's hospital provided preoperative informed consent to take part in the study in all cases after the Institutional Review Board Protocols DF/HCC 10-417 and DF/HCC 15-370B.

human_brain_glioma_Yuan_2018_Microwell : Tissue was procured from de-identified patients who provided written informed consent to participate in these studies through a protocol approved by the Columbia Institutional Review Board (IRB-AAAJ6163). Research was conducted in accordance with the principles of the Declaration of Helsinki.

human_brain_microglia_Gerrits_2021_10x : Brain tissue for snRNAseq was obtained from the NeuroBiobank of the Institute Born-Bunge (NBB-IBB), Wilrijk (Antwerp), Belgium (ID: BB190113) and donors gave informed consent to donate their brain to the NBB-IBB. Ethical approval was granted by the medical ethics committee of the Hospital Network Antwerp (ZNA, approval numbers 2805 and 2806). The study was compliant with the World Medical Association Declaration of Helsinki on Ethical Principles for Medical Research Involving Human Subjects.

human_brain_Frontaltemporal_Gerrits_2022_10x : Brain autopsy was carried out according to the Legal and Ethical Code of Conduct of the Netherlands Brain Bank (NBB)、 From the NeuroCEB brain bank in Paris, brain tissue of the frontal cortex of five FTD-GRN donors was obtained and used for snRNA-seq. The brain donation program is supported by patient associations, and autopsies are performed in accordance with the current French regulation

human_brain_glial_Venteicher_Smartseq2 : Patients at Massachusetts General Hospital gave consent preoperatively in all cases according to Institutional Review Board Protocol 1999P008145.

human_brain_GBM_Sankowski_2019_mCEL-seq2 : All experiments were conducted in accordance with the institutional review board of the University of Freiburg Medical Center.

human_brain_entorhinalcortex_Grubman_2019_DroNc-seq : Entorhinal cortex tissue from post-mortem Alzheimer's disease and non-diseased age-matched individuals was obtained from the Victorian Brain Bank (ethics approval for patient tissue banking and consent, University of Melbourne HREC Approval No.: 1545740; approval for transcriptomic analysis using banked tissue, Monash University MUHREC 2016–0554.

human_brain_Leng_2021_10x : This study was approved by the University of Sao Paulo institutional review board and deemed nonhuman participant research by UCSF

human_brain_Alsema_2020_10x : Autopsy brain specimens from the superior parietal lobe (LPS) and the superior frontal gyrus (GFS) were obtained from 25 donors of the Netherlands Brain Bank (NBB)1 and two donors of the NeuroBiobank of the Institute Born-Bunge (NBB-IBB, Wilrijk, Antwerp, Belgium, ID: BB190113). All donors have given informed consent for autopsy and use of their brain tissue for research purposes. The performed procedures and research protocols were approved by the corresponding ethical committees of the NBB

human_brain_glial_Fu_2021_PCR : The human fetal brain collection and research analysis was approved by the Reproductive Study Ethics Committee of Peking University Third Hospital (2012SZ-013). All human fetal brain samples used in this study were determined by clinicians with no obvious brain disease and were obtained with the informed consent from the donors. De-identified tissue samples were transported to the laboratory within several hours. Experimental protocols were in compliance with the International Review Board.

human_brain_source_Fan_2018 : Human embryonic brains were obtained from the Third Hospital of Peking University with agreement of the donors.

human_mouse_brain_Welch_2019_10x : Nuclei suspensions for the human substantia nigra experiments were generated from seven tissue donors (five males, three females, age range 18-75), supplied by the University of Maryland Brain Bank, through the NIH NeuroBioBank. Although all seven donors were coded as neurotypical controls, information provided with the tissue revealed that donor 5828 suffered a traumatic brain injury at the time of death, while donor 5840 was diagnosed

at autopsy with cerebral amyloid angiopathy. This work was determined by the Office of Research Subjects Protection at the Broad Institute not to meet the definition of human subjects research (project ID NHSR-4235).

human_brain_Trevino_cortex_2021_10x : De-identified tissue samples were obtained at Stanford University School of Medicine from elective pregnancy terminations under a protocol approved by the Research Compliance Office at Stanford University

human_brain_hippocampus_Zhong_2019_ATAC-seq : The de-identified human tissue collection and research protocols were approved by the Reproductive Study Ethics Committee of Beijing Anzhen Hospital and the institutional review board (ethics committee) of the Institute of Biophysics. The informed consent was designed as recommended by the ISSCR guidelines for fetal tissue donation and fetal tissue samples were collected after the donor patients signing an informed consent document that was in strict observance of the legal and institutional ethical regulations for samples from elective pregnancy terminations at Beijing Anzhen Hospital, Capital Medical University. All samples used in these studies had not been involved in any other procedures. All the protocols were in compliance with the Interim Measures for the Administration of Human Genetic Resources, administered by the Ministry of Science and Technology of China.

human_brain_RadialGlia_Nowakowski_2017_FluidigmC1 : De-identified tissue samples were collected with previous patient consent in strict observance of the legal and institutional ethical regulations. Protocols were approved by the Human Gamete, Embryo, and Stem Cell Research Committee (institutional review board) at the University of California, San Francisco.

human_brain_GBM_Zhao_2020_Microwell : This work was approved by the Columbia University Irving Medical Center Institutional Review Board before commencing the study.

human_brain_vascular_winkler_2022_10x : Human brain tissue specimens and clinical data were obtained from the University of California San Francisco with protocols approved from the institutional review board and ethics committee (IRB 10-01318 and 10-02012). All tissues were acquired from patients undergoing neurosurgical operations and written informed consent was obtained prior to the procedure permitting collection of tissue specimens for the purposes of research.

human_brain_glioma_Filbin_2018_Smartseq2 : Patients and their parents at Boston Children's hospital and the Medical University of Vienna were consented preoperatively in all cases according to Institutional Review Boards. F

human_brain_Glioblastoma_Darmanis_2017_SmartSeq2 : Informed consent was obtained from all subjects.

human_brain_Oligodendrogliomas_Tirosh_2016_Smartseq2_STRT-seq : Human tissue was obtained from the Massachusetts General Hospital according to an Institutional Review Board-approved protocol (1999P008145) and informed consent was obtained from all patients.

human_brain_cerebral_organoids_He_2021_10x_Visium_iTracer_perturb : Permission for this work with human iPSC lines was obtained through the Sachsisches Staatsministerium fur Umwelt und Landwirtschaft (Az. 55-8811.72/26, Az. 55-8811.72/26/382, Az. 55-8811.72/26/393 and 54-8452/26/7), the Swiss Federal Office for the Environment (A120821-08, A192559-01), the Ethics Committee of Northwest and Central Switzerland (2019-01016) and the Swiss Federal Office of Public Health.

human_brain_cerebral_organoids_Fleck_2022_10x_ATAC : The use of human ES cells for the generation of brain organoids was approved by the ethics committee of northwest and central Switzerland (2019-01016) and the Swiss federal office of public health.

human_chimp_macaque_PFC_Ma_2022_ATAC-seq_10x : Human tissues were collected following the guidelines provided by the Yale Human Investigation Committee for the Sestan or Girgenti laboratory collection. Human tissues were collected and handled in accordance with ethical guidelines and regulations for the research use of human brain tissue set forth by the NIH (http://bioethics.od.nih.gov/humantissue.html) and the WMA Declaration of Helsinki (http://www.wma.net/en/30publications/10policies/b3/index.html). Appropriate informed consent was obtained, and all available non-identifying information was recorded for each specimen.

human_chimpanzee_bonobo_macaque_brain_source_Kanton_2019 : the use of human ESCs for the generation of cerebral organoids was approved by the ethics committee of the Robert Koch Institut (https://www.rki.de/DE/Content/Gesund/Stammzellen/Register/reg-20161027-Paeaebo.html) as well as by the Ethics committee of northwest and central Switzerland (2019-01016) and the Swiss federal office of public health.

human_mouse_brain_SomatosensoryCortex_Hochgarner_2017_STRT-seq : Postmortem human brain tissue was provided to the Allen Institute for Brain Science by the San Diego Medical Examiner's (SDME) office after obtaining permission for tissue collection from decedent next-of-kin. Tissue specimens were de-identified and assigned a numerical ID, and the Allen Institute for Brain Science obtained the tissue under a legal agreement that prevents SDME from sharing the key to the code or any identifying information about tissue donors. The collection and use of postmortem human brain tissue for research purposes was reviewed by the Western Institutional Review Board (WIRB). WIRB determined that, in accordance with federal regulation 45 CFR 46 and associated guidance, the use of and generation of data from de-identified specimens from deceased individuals does not constitute human subjects research requiring IRB review.

human_mouse_brain_source_Gaublomme_2019 : The studies were conducted under Rush University IRB approvals L91020181 and L86121802. We have complied with all relevant ethical regulations and informed consent was obtained. We used frozen brain tissue banked by two prospective studies of aging: the Religious Order Study (ROS) and the Memory and Aging Project (MAP), which recruit non-demented older individuals (age > 65

human_brain_frontoparietal_Li_2018 : Tissue was collected after obtaining parental or next of kin consent and with approval by the institutional review boards at the Yale University School of Medicine, the National Institutes of Health, and at

each institution from which tissue specimens were obtained. Tissue was handled in accordance with ethical guidelines andregulations for the research use of human brain tissue set forth by the NIH (http://bioethics.od.nih.gov/humantissue.html) and the WMA Declaration of Helsinki (http://www.wma.net/en/30publications/10policies/b3/index.html).

human_brain_source_Zhong_2018 : The human embryo collection and research analysis was approved by the Reproductive Study Ethics Committee of Peking University Third Hospital (2012SZ-013 and 2017SZ-043) and Beijing Anzhen Hospital (2014012x). The informed consent was designed as recommended by the ISSCR guidelines for fetal tissue donation. Informed consent for fetal tissue procurement and research was obtained from the patient after her decision to legally terminate her pregnancy but before the abortive procedure. Fetal cortical tissue samples were collected after the donor patients signed an informed consent document that was in strict observance of the legal and institutional ethical regulations for elective pregnancy termination specimens at Peking University Third Hospital and Beijing Anzhen Hospital, Capital Medical University. All samples used in these studies had not been involved in any other procedures. All the protocols were in compliance with the 'Interim Measures for the Administration of Human Genetic Resources' administered by the Chinese Ministry of Health.

brain_Bhaduri2020_Human_10x : All primary tissue was obtained and processed as approved by the UCSF Human Gamete, Embryo and Stem Cell Research Committee (GESCR, approval 10-05113). All experiments were performed in accordance with protocol guidelines. Informed consent was obtained before sample collection for the use of all tissue samples within this study. First and second trimester human cortex tissue was collected from elective pregnancy termination specimens from San Francisco General Hospital and the Human Developmental Biology Resource (HDBR). Tissue was collected only with previous patient consent for research and in strict observation of legal and institutional ethical regulations.

human_brain_cerebralcortex_Delgato_2022 : Deidentified tissue samples were collected with previous patient consent in strict observance of the legal and institutional ethical regulations. Protocols were approved by the Human Gamete, Embryo, and Stem Cell Research Committee (Institutional Review Board) at the University of California, San Francisco

human_brain_Hippocampus_Su_2022_10x : De-identified human hippocampal tissue specimens were collected and processed under protocols approved by the Institutional Review Boards of the University of Pennsylvania and the Children's Hospital of Philadelphia. A total of 75 human hippocampal specimens between the ages of 0.1 to 95 years old were used in this study, including 40 post-mortem specimens from subjects free from neurological disorders and 8 post-mortem specimens from AD patients (Braak stage ranging from III to VI) for snRNA-seq, and 25 post-mortem specimens from subjects free from neurological disorders and 2 surgical specimens from epilepsy patients for immunohistological and in situ analyses (Table S1). Samples were from tissue banks at the Children's Hospital of Philadelphia, the Johns Hopkins University Pathology Archive, the Lieber Institute for Brain Development, the NIH NeuroBioBank at the University of Pittsburgh Brain Tissue Donation Program, the University of Maryland Brain and Tissue Bank, the University of Miami Brain Endowment Bank, the Harvard Brain Tissue Resource Center, the Human Brain and Spinal Fluid Resource Center at the VA West Los Angeles Healthcare Center, and the Mount Sinai School of Medicine (Table S1). Informed consent for each specimen was obtained by its corresponding institution prior to tissue collection.

human_brain_hippocampus_YiZhou_2022_10x : De-identified human tissue specimens were collected and processed under protocols approved by the Institutional Review Boards of the University of Pennsylvania and the Children's Hospital of Philadelphia. A total of 62 human postmortem hippocampal specimens taken between GW20 and 92 years of age, including 54 specimens from individuals free from neurological disorders and 8 specimens from patients with Alzheimer's disease, were used for snRNA-seq and immunohistological analyses (Supplementary Table 1). Specimens were collected from tissue banks at the Children's Hospital of Philadelphia, the Johns Hopkins University Pathology Archive, the Lieber Institute for Brain Development, and the NIH NeuroBioBank at the following repositories: University of Pittsburgh Brain Tissue Donation Program, the University of Maryland Brain and Tissue Bank, the University of Miami Brain Endowment Bank, the Harvard Brain Tissue Resource Center, the Human Brain and Spinal Fluid Resource Center at the VA West Los Angeles Healthcare Center, and the Mount Sinai School of Medicine. All embryonic tissues were from diagnostic autopsies. As postmortem interval could affect results of snRNA-seq51 and immunohistology analysis (for example, of DCX52), we tried to collect specimens with as short postmortem intervals as possible (listed in Supplementary Table 1). In addition, fresh surgically resected human hippocampal tissue from 10 patients between the ages of 2 and 61 years were used for ex vivo slice culture, collected from the Children's Hospital of Philadelphia and the Hospital of the University of Pennsylvania (Supplementary Table 1). Informed consent for each specimen was obtained by its corresponding institution prior to tissue collection.

human_brain_Pituitary_gland_Zhang_2020_STRTseq : The donors in this study were pregnant women who could not continue pregnancy because of their own diseases (such as cervical insufficiency, inevitable abortion, infection, eclampsia, as examples). All patients voluntarily donated the fetal tissues and signed informed consents. This study was approved by the Reproductive Study Ethics Committee of Peking University Third Hospital (2017SZ-043).

human_brain_Smith_2022_10x : This study was carried out in accordance with the Regional Ethics Committee and Imperial College Use of Human Tissue guidelines. Cases were selected from the London Neurodegeneration (King's College London) and Parkinson's UK (Imperial College London) Brain Banks

human_brain_fetal_Braun_2022_10x : For tissue collected at Karolinska Institute, patients seeking abortion at the gynecology clinic were asked about their interest in donating the aborted tissue to research. Patients that agreed, signed a written consent after receiving information, both written and oral, given by a physician or midwife. The use of abortion material was approved by the Swedish Ethical Review Authority and the National Board of Health and Welfare

human_mouse_macque_brain_Bakken_2021_SMARTer : Postmortem adult human brain tissue from three donors was collected after obtaining permission from decedent next-of kin. Postmortem tissue collection was performed in accordance with the provisions of the United States Uniform Anatomical Gift Act of 2006 described in the California Health and Safety Code section 7150 (effective 1/1/2008) and other applicable state and federal laws and regulations. The Western Institutional Review Board reviewed tissue collection processes and determined that they did not constitute human subjects research requiring institutional review board (IRB) review

human_brain_hippocampus_Franjic_2021_10x : All human (Homo sapiens) brain specimens used for snRNA-seq

transcriptome and DCX immunostaining (Tables S1 and S3) were de-identified and collected from clinically unremarkable donors and one case that died in status epilepticus. Tissue was collected following the guidelines provided by the Yale Human Investigation Committee (HIC) for the Sestan and Rakic collection or by the European Union for DeFelipe's samples from Spain. Tissue was collected and handled in accordance with ethical guidelines and regulations for the research use of human brain tissue set forth by the NIH (http://bioethics.od.nih.gov/humantissue.html) and the WMA Declaration of Helsinki (https://www.wma.net/en/30publications/10policies/b3/index.html). Appropriate informed consent was obtained and

mouse_brain_dentate_gyrus_Hochgerner_2018_10x_C1 : All experimental procedures followed the guidelines and recommendations of Swedish animal protection legislation and were approved by the local ethical committee for experiments on laboratory animals (Stockholms Norra Djurförsöksetiska nämnd, Sweden).

human_mouse_brain_midbrain_LaManno_2016_STRT-seq : Human fetal tissues were collected from routine termination of pregnancies at Addenbrooke's Hospital (Cambridge) and dissected in HIBERNATE media. Samples for single cell analysis were screened for biohazards and then shipped overnight on ice (in HIBERNATE media) to Sweden. Ethical approval for the use of postmortem human fetal tissue was provided by the National Research Ethics Service Committee East of England - Cambridge Central (Local Research Ethics Committee, reference no. 96/085).

human_mouse_brain_neocortex_Berg_2021_SMART-Seq : All procedures were carried out in accordance with the Institutional Animal Care and Use Committee at the Allen Institute for Brain Science.

Note that full information on the approval of the study protocol must also be provided in the manuscript.

Field-specific reporting

Please select the one below that is the best fit for your research. If you are not sure, read the appropriate sections before making your selection.

☒ Life sciences ☐ Behavioural & social sciences ☐ Ecological, evolutionary & environmental sciences

For a reference copy of the document with all sections, see nature.com/documents/nr-reporting-summary-flat.pdf

Life sciences study design

All studies must disclose on these points even when the disclosure is negative.

Sample size	Complete sample size information of scRNA-seq has been included in Supplementary Table 8. All large, publicly available single-cell/single-nuclei brain datasets were included in the Brain Cell Atlas.<br>Sample size information of immunostaining experiments has been indicated in figure legends.
Data exclusions	No data were excluded from the analysis.
Replication	1 patient, 2 replicates in total were done for Fig. 3k immunostaining.<br>3 monkeys, 3 replicates in total were done for Supplementary Fig. 7c immunostaining.<br>4 monkeys, 6 replicates in total were done for Supplementary Fig. 7d immunostaining.<br><br>1 patient, 2 replicates in total were done for Fig. 4f immunostaining.<br>2 patients, 2 replicates in total were done for Fig. 4g immunostaining.<br>2 patients, 4 replicates in total were done for Extended Data Fig. 3a immunostaining.<br>1 patient, 3 replicates in total were done for Extended Data Fig. 3b immunostaining.<br>1 patient, 1 replicate in total was done for Extended Data Fig. 5b immunostaining.<br><br>All attempts at replication were successful.
Randomization	There were no different experimental groups, such as treated versus control, in the newly generated Brain Cell Atlas data.
Blinding	As there were no different experimental groups, there was also no blinding.

Reporting for specific materials, systems and methods

We require information from authors about some types of materials, experimental systems and methods used in many studies. Here, indicate whether each material, system or method listed is relevant to your study. If you are not sure if a list item applies to your research, read the appropriate section before selecting a response.

Materials & experimental systems

n/a	Involved in the study	
☐	☒	Antibodies
☒	☐	Eukaryotic cell lines
☒	☐	Palaeontology and archaeology
☐	☒	Animals and other organisms
☒	☐	Clinical data
☒	☐	Dual use research of concern
☒	☐	Plants

Methods

n/a	Involved in the study	
☒	☐	ChIP-seq
☒	☐	Flow cytometry
☒	☐	MRI-based neuroimaging

Antibodies

Antibodies used

Mouse anti-SOX-2, clone 10H9.1 -2523218, Millipore, Cat# MAB4423, 1:1000 dilution
Chicken anti-Glial Fibrillary Acidic Protein, Millipore, Cat# AB5541, 1:2000 dilution
Rabbit monoclonal anti-MASH1 (ASCL1), Abcam: clone EPR19840, Cat# ab211327, 1: 100 dilution
Rabbit anti-Ki67, clone SP6, Abcam, Cat# ab16667, 1: 200 dilution
Mouse monoclonal anti-Ki67, Leica Biosystems, Cat# NCL-L-Ki67-MM1
Goat anti-Osteopontin (spp1), R&D, Cat# AF808, 1:1000 dilution
Rabbit anti-PCDH9, Proteintech, Cat# 25090-1-AP, 1:500 dilution
Mouse Anti-Myelin Associated Glycoprotein Antibody, Millipore, clone 513, Cat# MAB1567, 1:500 dilution
Goat anti- AIF-1/Iba1, Novus Biologicals, Cat# NB100-1028, 1:200 dilution
Goat doublecortin (C-18), Santa Cruz, Cat# sc-8066, 1: 1000 dilution
Goat anti-Iba1,Novus, Cat# NB100-1028, 1: 100 dilution

Validation

SOX-2 Millipore: Validated by the vendor with western blotting in mouse embryonic stem cell lysate. https://www.sigmaaldrich.cn/CN/zh/product/mm/mab4423
Glial Fibrillary Acidic Protein Millipore: This Anti-Glial Fibrillary Acidic Protein Antibody is validated by the vendor for use in IC, IH, IH(P), WB for the detection of GFAP.
https://www.merckmillipore.com/HK/en/product/Anti-Glial-Fibrillary-Acidic-Protein-Antibody,MM_NF-AB5541
MASH1 (ASCL1) Abcam: Validated by immunohistochemistry staining in mouse lungs as a pulmonary neuroendocrine cell. DOI: 10.1002/stem.2744 https://www.abcam.com/mash1achaete-scute-homolog-1-antibody-epr19840-ab211327.html
Ki67 Abcam: Validated by the vendor with the detection of protein depletion in gene knockout cells. https://www.abcam.com/Ki67-antibody-SP6-ab16667.html
Ki67 Leica Biosystems: Validated in the references provided by the vendor. https://www.labome.com/product/Leica-Biosystems/NCL-L-Ki67-MM1.html
Osteopontin (spp1): Validated by the vendor with immunohistochemistry staining in mouse thymus. https://www.citeab.com/antibodies/694271-af808-mouse-osteopontin-opn-antibody?des=2802ad49dc501b71
PCDH9: Validated by the vendor with IHC staining in human gliomas and mouse brain.
https://www.ptglab.com/products/PCDH9-Antibody-25090-1-AP.htm
MAG: Validated by the vendor with immunohistochemistry staining in mouse sensory neurons and rat hippocampus.
https://www.sigmaaldrich.cn/CN/zh/product/mm/mab1567
Goat anti- AIF-1/Iba1, Novus Biologicals: This anti- AIF-1/Iba1 Antibody is validated by the vendor for use in IC, IH, IH(P), WB.
https://www.novusbio.com/products/aif-1-iba1-antibody_nb100-1028
Goat doublecortin (C-18) Santa Cruz: Validated in the references provided by the vendor.
DOI: 10.1002/glia.22906; DOI: 10.1007/s12035-016-0151-5; DOI: 10.1523/JNEUROSCI.0343-15.2015. https://www.scbt.com/p/doublecortin-antibody-c-18
Iba1 Novus :Validated by the vendor. IF: Human tumor microarrays. Mouse brain. IHC: Rat and mouse brain, human Spleen. https://www.novusbio.com/products/aif-1-iba1-antibody_nb100-1028

Animals and other research organisms

Policy information about studies involving animals; ARRIVE guidelines recommended for reporting animal research, and Sex and Gender in Research

Laboratory animals

We used adult macaque monkeys (Macaca fascicularis) of 2 males at the age of 6 YO, 1 male of 7 YO and 1 male of 15 YO.

Wild animals

The study did not involve wild animals.

Reporting on sex

Only males were used for the immunostaining experiment, based on the availability of the animals.

Field-collected samples

The study did not involve samples collected from the field.

Ethics oversight

All experimental procedures were approved by and in accordance with the Animal Care and Use Committee of Zhongshan Ophthalmic Center, Sun Yat-sen University. The study was performed in accordance with the Public Health Service Guide to the Care and Use of Laboratory Animals.

mouse_brain_dentate_gyrus_Hochgerner_2018_10x_C1 : All experimental procedures followed the guidelines and recommendations of Swedish animal protection legislation and were approved by the local ethical committee for experiments on

laboratory animals (Stockholms Norra Djurförsöksetiska nämnd, Sweden).

human_mouse_brain_midbrain_LaManno_2016_STRT-seq : Human fetal tissues were collected from routine termination of pregnancies at Addenbrooke's Hospital (Cambridge) and dissected in HIBERNATE media. Samples for single cell analysis were screened for biohazards and then shipped overnight on ice (in HIBERNATE media) to Sweden. Ethical approval for the use of postmortem human fetal tissue was provided by the National Research Ethics Service Committee East of England - Cambridge Central (Local Research Ethics Committee, reference no. 96/085).

mouse_brain_Cerebullum_Kozareva_2021_10x : All experiments were approved by and in accordance with Broad IACUC protocol number 012-09-16

mouse_brain_Striatum_Microglia_Badimon_2020_10x : All animal protocols were approved by the IACUC at Icahn School of Medicine at Mount Sinai and were performed in accordance with NIH guidelines

mouse_brain_cortex_Zheng_2017_10x : No mention about the animals. The Institutional Review Board at the Fred Hutchinson Cancer Research Center approved the study on transplant samples. The procedures followed were in accordance with the Declaration of Helsinki of 1975 and the Common Rule. Samples were obtained after patients had provided written informed consent on molecular analyses.

mouse_brain_CTX-HPF_AllenBrain_2020_10x : All procedures were carried out in accordance with Institutional Animal Care and Use Committee protocols at the Allen Institute for Brain Science.

mouse_brain_Saunders_2018_Drop-Seq : All experiments were approved by and in accordance with Harvard Medical School IACUC protocol number IS00000055-3.

mouse_brain_hypothalamus_Kim_2020_10x : All experimental animal procedures were approved by the Johns Hopkins University Institutional Animal Care and Use Committee.

mouse_brain_dorsal_raphe_nucleus_Huang_2020_InDrops : All procedures were performed following protocols approved by the Harvard Standing Committee on Animal Care following guidelines described in the U.S. National Institutes of Health Guide for the Care and Use of Laboratory Animals

mouse_brain_Hippocampus_Stickels_2021_Slide_seqV2 : All procedures involving animals at the Broad Institute were conducted in accordance with the US National Institutes of Health Guide for the Care and Use of Laboratory Animals under protocol number 0120-09-16. All procedures involving animals at Harvard University were handled according to protocols approved by the Institutional Animal Care and Use Committee of Harvard University (protocol number 11-03) and followed the guidelines set forth in the US National Institute of Health Guide for the Care and Use of Laboratory Animals.

mouse_brain_LaManno_2021_10x_HybISS : All mouse procedures were approved by the Stockholm ethics committee (N68/14; Stockholms djurförsöksetiska nämnd) and followed Directive 2010/63/EU of the European Parliament and of the Council, the Swedish Animal Welfare Act (Djurskyddslagen: SFS 1988:534), the Swedish Animal Welfare Ordinance (Djurskyddsförordningen: SFS 1988:539) and the provisions regarding the use of animals for scientific purposes (DFS 2004:15 and SJVFS 2012:26).

mouse_brain_Chu_2019_10x : All mouse experiments were approved by, and performed in accordance with, the Institutional Animal Care and Use Committee guidelines at Weill Cornell Medicine.

mouse_brain_VMH_Kim_2019_10x_smart_seqFISH : All procedures were performed in accordance with NIH guidelines and approved by the Institutional Animal Care and Use Committee (IACUC) at the California Institute of Technology (Caltech) and Allen Institute for Brain Science (AIBS)

mouse_brain_Brain_Fei_2022_Microwell : All experiments performed in this study were approved by the Animal Ethics Committee of 590 Zhejiang University. All experiments conformed to the relevant regulatory standards at 591 Zhejiang University Laboratory Animal Center.

mouse_brain_Striatum_Wertz_2020_10x : All mouse husbandry and experimental procedures were conducted with the approval of the Massachusetts Institute of Technology Animal Care and Use Committee.

mouse_brain_source_Amit_2018 : All experimental procedures followed the guidelines and recommendations of Swedish animal protection legislation and were approved by the local ethical committee for experiments on laboratory animals (Stockholms Norra Djurförsöksetiska nämnd, Sweden).

mouse_brain_spinalcord_Rosenberg_2018_SPLiT-seq : All animal procedures were done using protocols approved by the Institutional Animal Care and Use Committee at the University of Washington.

mouse_brain_LGN_Kalish_2018_InDrops : All experiments using animals were performed according to protocols approved by the Harvard Medical Area Institutional Animal Care and Use Committee.

mouse_brain_Borm_2022_EEL_FISH : Animal handling and tissue collection methods followed the guidelines and recommendations of local animal protection legislation and were approved by the local committee for ethical experiments on laboratory animals (Stockholms Norra Djurförsöksetiska nämnd, Sweden, N 68/14).

mouse_brain_Cortex_Jin_2020_Perturb : All animal experiments were performed according to protocols approved by the Institutional Animal Care and Use Committees (IACUC) of Harvard University and of the Broad Institute of MIT and Harvard.

mouse_brain_Astrocytes_Hasel_10x : All animal procedures were in accordance with the guidelines provided by the National Institute of Health as well as NYU Langone School of Medicine's Administrative Panel on Laboratory Animal Care.

mouse_brain_DRG_Wang_2021_10x_smart : C57BL/6J mice were used in the experiments, according to the guidelines of the Committee of Use of Laboratory Animals and Common Facilities, Institute of Neuroscience, Chinese Academy of Sciences, Shanghai, China.

mouse_brain_auditory_cortex_Kalish_2020_InDrops : All experiments using animals were performed according to protocols approved by the Harvard University Institutional Animal Care and Use Com-mittee. T

mouse_brain_microglia_Hammond_2018_10x : All experiments were reviewed and overseen by the institutional animal use and care committee at Boston Children's Hospital in accordance with all NIH guidelines for the humane treatment of animals.

mouse_brain_GABAergicNeuron_Bandler_2021_10x : All mouse colonies were maintained in accordance with protocols approved by the Bavarian government at the Max Planck Institute of Neurobiology or the IACUC at the NYU Grossman School of Medicine.

mouse_brain_choroid_plexus_Dani_2021_10x : All mouse work was performed in accordance with the Institutional Animal Care and Use Committees (IACUC) and relevant guidelines of Boston Children's Hospital and Masaryk University.

mouse_brain_barrel_somatosensory_cortex_Gunner_2019_InDrops : All experiments were performed in accordance with the animal care and use committees and under NIH guidelines for proper animal welfare.

mouse_brain_hippocampus_Habib_2020_10x : Animal handling complied with the regulations formulated by the Institutional Animal Care and Use Committee of the Weizmann Institute of Science

mouse_brain_NSCs_ZhangY_2020_10x : All experiments were performed according to protocols approved by the Institutional Animal Care and Use Committee at University of California at Santa Cruz, and were performed in accordance with institutional and federal guidelines. Experiments performed at Fudan University were in accordance with institutional guideline.

mouse_brain_suprachiasmatic_nucleus_Wen_2020_10x_Drop_LCM : All animal experiments conform with the agreement of the Committee on Laboratory Animals of the Institute of Neuroscience, Chinese Academy of Sciences (IACUC no. NA-023-2016).

mouse_brain_Guldner_2020_CITE : All animal studies were performed ethically and in compliance with IACUC protocol approved by the University of Notre Dame IACUC committee.

mouse_brain_VanHove_2019_10x : Mouse experiments were approved and performed in accordance with the Ethische Commissie Dierproeven at Vrije Universiteit Brussel.

mouse_brain_visual_cortex_Hrvatin_2017_InDrops : Animal experiments were approved by the National Institutes of Health and the Harvard Medical School Institutional Animal Care and Use Committee and followed the ethical guidelines described in the US National Institutes of Health Guide for the Care and Use of Laboratory Animals (https://grants.nih.gov/grants/olaw/guide-for-the-care-and-use-of-laboratory-animals.pdf).

mouse_brain_cortical_inhibitory_interneurons_Mayer_2018_10x_drop_smart : All mouse colonies were maintained in accordance with protocols approved by the Institutional Animal Care and Use Committee at the NYU School of Medicine.

mouse_brain_hindbrain_Vladoiu_2019_10x : All mouse breeding and procedures were approved by The Centre for Phenogenomics (Toronto).

mouse_brain_Zheng_2021_10x : All animal experimental procedures were conducted in accordance with the Guide for the Care and Use of Laboratory Animals of the National Institutes of Health and approved by the Animal Experiments Ethics Committee of Tianjin Medical University General Hospital, which were in compliance with the ARRIVE guidelines 2.0: Updated guidelines for reporting animal research.

mouse_brain_interneuron_Allaway_2021_10x : All mouse colonies were maintained in accordance with protocols approved by the Institutional Animal Care and Use Committees at Harvard Medical School and the Broad Institute of MIT and Harvard.

mouse_brain_OlfactoryBulb_Tepe_2018_10x : All animals used in this study were housed and handled according to US Department of Health and Human Services and Baylor College of Medicine IACUC guidelines.

mouse_brain_Hippothalamus_Romanov_2020_10x : Experiments on live animals conformed to the 2010/63/EU European Communities Council Directive and were approved by the Austrian Ministry of Science and Research (66.009/0145-WF/II/3b/2014, and 66.009/0277-WF/V/3b/2017). Particular effort was directed towards minimizing the number of animals used and their suffering during experiments.

mouse_brain_cortex_Hu_2017_sNucDrop : Experiments were conducted in accordance with the ethical guidelines of the National Institutes of Health and with the approval of the Institutional Animal Care and Use Committee of the University of Pennsylvania.

mouse_brain_ NAc_Chen_2021_10x : All experiments were conducted in accordance with the National Institutes of Health Guide for Care and Use of Laboratory Animals and were approved by the Institutional Animal Care and Use Committees of Boston Children's Hospital and Harvard Medical School.

mouse_brain_SubVentricularZone_Mizrak_2019_Microwell : All experiments were performed in accordance with institutional and national guidelines for animal use and approved by IACUC at Columbia University and the cantonal veterinary office of Basel-Stadt.

mouse_brain_Cortex_DiBella_2021_10x_scATAC : All animal experiments were conducted according to protocols approved by the Institutional Animal Care and Use Committee (IACUC) of Harvard University.

mouse_brain_Chung_2021_inCITE-seq : All mouse experiments were approved by and performed in accordance with the Institutional Animal Care and Use Committee guidelines at Weill Cornell Medicine.

mouse_brain_Keren-Shaul_2017_MARS : All experiments detailed herein complied with the regulations formulated by the Institutional Animal Care and Use Committee (IACUC) of the Weizmann Institute of Science.

mouse_bran_microglia_Ximerakis_2019_10x : All experimental procedures were approved in advance by the Animal Care and Use Committee of Harvard University (AEP no. 10–23) and are in compliance with federal and state laws.

mouse_brain_nucleus_accumbens_Avey_2018_drop : All animal care and experimental procedures were approved in advance by the National Institute of Health and Washington University School of Medicine Institutional Animal Care and Use Committee (Protocol #20170030).

mouse_brain_tumour_Quintana_2022_10x : All animal experiments were approved by the University of Glasgow Ethical Review Committee and performed in accordance with the home office guidelines, UK Animals (Scientific Procedures) Act, 1986 and EU directive 2010/63/EU.

mouse_brain_source_Bhattacherjee_2019 : The animal use and experiments were conducted in compliance with the institutional IACUC Committee (of the HCCM).

mouse_brain_preoptic_region_Moffitt_2018_10x : Animal care and experiments were carried out in accordance with NIH guidelines and were approved by the Harvard University Institutional Animal Care and Use Committee (IACUC).

mouse_brain_HPF_PFC_Joglekar_2021_10x_PacBio_Visium : All experiments were conducted in accordance with relevant NIH guidelines and regulations, related to the Care and Use of Laboratory Animals tissue. Animal procedures were performed according to protocols approved by the Research Animal Resource Center at Weill Cornell College of Medicine.

mouse_brain_Hypothalamus_Rossi_2019_Drop-Seq : All procedures were conducted in accordance the NIH guide for the care and use of laboratory animals and were approved by the Institutional Animal Care and Use Committee at the University of North Carolina.

mouse_brain_cortex_Tasic_2019_SMARTer : All procedures were carried out in accordance with Institutional Animal Care and Use Committee protocols 1508, 1510 and 1511 at the Allen Institute for Brain Science and Janelia Research Campus.

mouse_brain_hippocampus_Miguel_2021_10x : All of the experiments were performed in accordance with institutional guidelines approved by the VA Palo Alto Committee on Animal Research. Mice were randomly assigned to the experimental conditions.

mouse_brain_SubventricularZone_Zywitza_2018_Drop-seq : Experiments involving animals were performed according to institutional guidelines following approval by local authorities (X9017/12).

mouse_brain_osmosensory_Pool_2020_10x : All animal care and experimental procedures were carried out in accordance with the US NIH guidance for the care and use of laboratory animals and approved by the California Institute of Technology Animal Care and Use Committee (protocol no. 1694-14).

mouse_brain_neocortex_Wittmann_2021_10x : All experiments were carried out in accordance with the European Communities Council Directive (86/609/EEC) and were approved by the government of Middle-Franconia.

mouse_brain_Cerebellum_Peng_2018_10x : All animal usage and manipulations followed guidelines of Institutional Animal Care and Use Committees at the Institute of Neuroscience, Chinese Academy of Sciences.

mouse_brain_Arcuate_Nucleus_Median_Eminence_Campbell_2017_drop : All animal care and experimental procedures were approved in advance by the National Institute of Health and Beth Israel Deaconess Medical Center Institutional Animal Care and Use Committee.

mouse_brain_Amygdala_Wu_2017_Drop-seq : All animal care and experimental procedures followed National Institutes of Health guidelines, and were approved by the UCLA Institutional Animal Care and Use Committee.

mouse_brain_Microglia_Sierksma_2022_10x : The local Ethical Committee of Laboratory Animals of the KU Leuven (governmental license LA1210591, ECD project number P202-2013) approved all animal experiments, following governmental and EU guidelines.

mouse_brain_neocortex_Loo_2019_drop : All procedures used in this study were approved by the Institutional Animal Care and Use Committee at the University of North Carolina at Chapel Hill.

mouse_brain_SpinalCords_Skinnider_2021_10x : Not Mentioned

mouse_brain_source_Fazel_2022 : All procedures and animal care were approved and performed in accordance with the University of California San Francisco Laboratory Animal Research Center (LARC) guidelines.

mouse_brain_Hippocampal_Mirabella_2021_10x : All experiments were performed using mice C57BL/6N (Charles River Laboratories) according to the guidelines established by the European Community Council (Directive 2010/63/EU of September 22nd,2010) and were approved by the Institutional Animal Care and Use Committee (IACUC, permission number 467 and 565) of the Humanitas Research Hospital and by the Italian Ministry of Health.

mouse_brain_Ding_10x : All animal-related work was performed under the guidelines of the Division of Comparative Medicine, with the protocol (# 0416-050-1) approved by the Committee for Animal Care of the Massachusetts Institute of Technology, and was consistent with the Guide for Care and Use of Laboratory Animals (1996 edition).

mouse_brain_Hypothalamus_Chen_2017_drop : All animal experiments followed the guidelines of the Institutional Animal Care and Use Committee at Harvard Medical School.

mouse_brain_developing_ZhangX_2022_10x : Mouse experiments were performed under protocols approved by Mayo Clinic Institutional Animal Care and Use Committee (protocol #A00003875-18).

mouse_brain_Golomb_2020_CITE : All animal studies were performed ethically and in compliance with IACUC protocol approved by the University of Notre Dame IACUC committee.

mouse_brain_nucleus_accumbens_Kim_2020_10x : All protocols were approved by Stanford University's Institutional Animal Care and Use Committee.

mouse_brain_OB_Mizrak_2020_SCOPE-seq : All experiments were performed according to protocols approved by IACUC at the Columbia University and the Memorial Sloan Kettering Cancer Center.

mouse_brain_Cerebellum_Wizeman_2019_10x : All procedures involving animals were approved by the Animal Care Committee at the University of Connecticut Health Center (protocol #101849–0621) and were in compliance with national and state laws and policies.

mouse_brain_hippocampus_Arneson_2018_drop : All experiments were performed in accordance with the United States National Institutes of Health Guide for the Care and Use of Laboratory Animals and were approved by the University of California at Los Angeles Chancellor's Animal Research Committee.

mouse_brain_source_Lui_2020_10x : All procedures, husbandry conditions, and housing conditions followed standard animal care and biosafety guidelines approved by Stanford University's Administrative Panel on Laboratory Animal Care and Administrative Panel on Biosafety in accordance with NIH guidelines.

mouse_brain_diencephalon_Guo_2019_10x : All animal procedures described herein were approved by the Animal Care Committee at the University of Connecticut Health Center.

mouse_brain_lateral_hypothalamic_area_Mickelsen_2019_10x : All experiments were performed in accordance with the ethical guidelines described in the National Institutes of Health's Guide for the Care and Use of Laboratory Animals and were approved by the Institutional Animal Care and Use Committee of the University of Connecticut and of the Jackson Laboratory (JAX) for Genomic Medicine.

mouse_brain_Cortex_Süß_2020_10x : All experiments were conducted according to the National Institutes of Health and the European (2010/63/EU) guidelines for the humane treatment of animals and approved by the local government commission of animal health.

mouse_brain_hypothalamic_Huisman_2019_10x : All mouse works were performed under approved protocols by the Institutional Animal Care and Use Committee of the Oregon Health and Science University and King's College London.

mouse_brain_EmbryonicCortex_Yuzwa_2017_Drop-Seq : All animal use was approved by the Animal Care Committee of the Hospital for Sick Children in accordance with the Canadian Council of Animal Care policies.

mouse_brain_CD45_cells_Jordão_2019_mCEL : All animal experiments were approved by the local administration and were performed in accordance to the respective national, federal and institutional regulations.

mouse_brain_forebrain_Li_2020_10x : These procedures were reviewed and approved by the Institutional Animal Care and Use Committee at Boston University School of Medicine.

mouse_brain_oligodendrocyte_Marques_2016_STRT : All experimental procedures performed followed the guidelines and recommendations of local animal protection legislation and were approved by the local committee for ethical experiments on laboratory animals (Stockholms Norra Djurförsöksetiska nämnd in Sweden).

mouse_brain_CortexHPF_Rosen_2022_10x : All experiments followed guidelines approved by the Washington University School of Medicine Animals Safety Committee (IACUC Animal Welfare Assurance # A-3381-01, protocol no. 20180120).

mouse_brain_forebrain_Dickel_2018_drop : All animal work was reviewed and approved by the Lawrence Berkeley National Laboratory Animal Welfare and Research Committee.

mouse_brain_SVZ_Kalamakis_2019_10x_smart : All procedures were approved by the Regierungpräsidium Karlsruhe.

mouse_brain_vascular_Vanlandewijck_2018_Smart-seq2 : Animal experiment protocols were approved by the Uppsala Ethical Committee on Animal Research (permit numbers C224/12 and C115/15) and the Stockholm North Animal Ethics committee (Stockholms Norra Djurförsöksetiska Nämnd), permit N150/14. All animal experiments were carried out in accordance with their guidelines.

mouse_brain_BasalGanglia_Wallace_2017_Drop-seq : All experimental manipulations were performed in accordance with protocols approved by the Harvard Standing Committee on Animal Care following guidelines described in the US NIH Guide for the Care and Use of Laboratory Animals.

mouse_brain_ Neocortex_Telly_2019_SMARTer : All experiments were approved by the Geneva Cantonal Veterinary Authorities, Switzerland and the Austrian Federal Ministry of Science and Research in accordance with the Austrian and EU animal laws.

mouse_brain_Mi_2018_SMARTer : All mouse colonies were maintained in accordance with protocols approved by UK Home Office

project licenses and the Institutional Animal Care and Use Committee (IACUC) at Yale University. Experimental protocols were approved by King's College London and Yale University, School of Medicine welfare committees.

mouse_brain_mycroglia_myeloid_Li_2018_smart : All animal studies were performed with approval from the Stanford Administrative panel on Laboratory Animal Care in accordance with institutional and national regulations.

mouse_brain_Astrocytes_Batiuk_2020_smart : All experiments were approved by the Ethical Research Committee of the KU Leuven and were in accordance with the European Communities Council Directive of 22 September 2010 (2010/63/EU) and with the relevant Belgian legislation (KB of 29 May 2013).

mouse_brain_Ikonomou_2020_10x : All mouse studies involving mice carrying GFP, CreERT2, Cre, and nT/nG transgenes were approved by the Institutional Animal Care and Use Committee of Boston University School of Medicine. All mouse studies involving mouse foregut explants were approved by the Institutional Animal Care and Use Committee of Cincinnati Children's Hospital.

mouse_brain_source_Kevin_2020 : A combined hippocampus, cortex, and ventricular zone pair from an E18 C57BL/6 mouse was obtained from BrainBits LLC (Leicestershire, UK).

mouse_brain_Amygdala_Peters_2022_mFISH : The animal study was reviewed and approved by the Regierung von Oberbayern under the license 55.2-2532.Vet_02-20-49.

mouse_brain_Rodriques_2019_Slide-seq : All procedures involving animals at MIT were conducted in accordance with the US National Institutes of Health Guide for the Care and Use of Laboratory Animals under protocol number 1115-111-18 and approved by the Massachusetts Institute of Technology Committee on Animal Care. All procedures involving animals at the Broad Institute were conducted in accordance with the US National Institutes of Health Guide for the Care and Use of Laboratory Animals under protocol number 0120-09-16.

human_mouse_brain_neocortex_Berg_2021_SMART-Seq : All procedures were carried out in accordance with the Institutional Animal Care and Use Committee at the Allen Institute for Brain Science.

mouse_brain_neurons_Peng_2021_SMART-seq : All experimental procedures related to the use of mice were conducted with approved protocols in accordance with NIH guidelines, and were approved by the Institutional Animal Care and Use Committee (IACUC) of the Allen Institute for Brain Science.

mouse_brain_NeuralCrest_Soldatov_2019_Smart-seq2 : Day of plug detection was considered as E0.5. All animal work was permitted by the Ethical Committee on Animal Experiments (Stockholm North committee) and conducted according to The Swedish Animal Agency's Provisions and Guidelines for Animal Experimentation recommendations.

mouse_brain_Hippocampus_ZhongS_2020_STRT-Seq : All animal experiments were carried out in full compliance with regulations of Institutional Animal Care and Use Committee of University of Science and Technology of China.

human_mouse_macque_brain_Bakken_2021_SMARTer : Postmortem adult human brain tissue from three donors was collected after obtaining permission from decedent next-of kin. Postmortem tissue collection was performed in accordance with the provisions of the United States Uniform Anatomical Gift Act of 2006 described in the California Health and Safety Code section 7150 (effective 1/1/2008) and other applicable state and federal laws and regulations. The Western Institutional Review Board reviewed tissue collection processes and determined that they did not constitute human subjects research requiring institutional review board (IRB) review
human_mouse_brain_SomatosensoryCortex_Hochgarner_2017_STRT-seq : Postmortem human brain tissue was provided to the Allen Institute for Brain Science by the San Diego Medical Examiner's (SDME) office after obtaining permission for tissue collection from decedent next-of-kin. Tissue specimens were de-identified and assigned a numerical ID, and the Allen Institute for Brain Science obtained the tissue under a legal agreement that prevents SDME from sharing the key to the code or any identifying information about tissue donors. The collection and use of postmortem human brain tissue for research purposes was reviewed by the Western Institutional Review Board (WIRB). WIRB determined that, in accordance with federal regulation 45 CFR 46 and associated guidance, the use of and generation of data from de-identified specimens from deceased individuals does not constitute human subjects research requiring IRB review.

human_mouse_brain_source_Gaublomme_2019 : The studies were conducted under Rush University IRB approvals L91020181 and L86121802. We have complied with all relevant ethical regulations and informed consent was obtained. We used frozen brain tissue banked by two prospective studies of aging: the Religious Order Study (ROS) and the Memory and Aging Project (MAP), which recruit non-demented older individuals (age > 65

human_mouse_brain_Welch_2019_10x : Nuclei suspensions for the human substantia nigra experiments were generated from seven tissue donors (five males, three females, age range 18-75), supplied by the University of Maryland Brain Bank, through the NIH NeuroBioBank. Although all seven donors were coded as neurotypical controls, information provided with the tissue revealed that donor 5828 suffered a traumatic brain injury at the time of death, while donor 5840 was diagnosed at autopsy with cerebral amyloid angiopathy. This work was determined by the Office of Research Subjects Protection at the Broad Institute not to meet the definition of human subjects research (project ID NHSR-4235).

mouse_brain_SNpc_Kamath_2022_10x_Slide : All housing and procedures involving rodents were conducted in accordance with the US National Institutes of Health Guide for the Care and Use of Laboratory Animals, under protocol no. 0129-09-16, and were approved by the Broad Institute Committee on Animal Care (IACUC). All NHP tissue was processed in compliance with the Broad Institute IBC (IBC, no. 2016-00127). All human tissue falls under a 'Not Engage' designation determined by the Broad Institute IACUC (Federal-wide assurance, no. FWA00014055).

mouse_brain_source_Habib_2017 : All procedures were carried out in accordance with Institutional Animal Care and Use Committee protocols 0703 and 1208 at the Allen Institute for Brain Science. Animals were provided food and water ad libitum and were maintained on a regular 12-h day/night cycle at no more than five adult animals per cage.

human_mouse_brain_Wheeler_2020_DropSeq_10x : Use of the tissues was approved by the Montreal Neurological Institute and Hospital (MNI/H) Neurosciences Research Ethics Board under REB approval ANTJ 1988/3.

Note that full information on the approval of the study protocol must also be provided in the manuscript.

Plants

Seed stocks

Report on the source of all seed stocks or other plant material used. If applicable, state the seed stock centre and catalogue number. If plant specimens were collected from the field, describe the collection location, date and sampling procedures.

Novel plant genotypes

Describe the methods by which all novel plant genotypes were produced. This includes those generated by transgenic approaches, gene editing, chemical/radiation-based mutagenesis and hybridization. For transgenic lines, describe the transformation method, the number of independent lines analyzed and the generation upon which experiments were performed. For gene-edited lines, describe the editor used, the endogenous sequence targeted for editing, the targeting guide RNA sequence (if applicable) and how the editor was applied.

Authentication

Describe any authentication procedures for each seed stock used or novel genotype generated. Describe any experiments used to assess the effect of a mutation and, where applicable, how potential secondary effects (e.g. second site T-DNA insertions, mosiacism, off-target gene editing) were examined.
